# Barriers and facilitators to clinical behaviour change by primary care practitioners: a theory-informed systematic review of reviews using the Theoretical Domains Framework and Behaviour Change Wheel

**DOI:** 10.1186/s13643-022-02030-2

**Published:** 2022-08-30

**Authors:** Melissa Mather, Luisa M. Pettigrew, Stefan Navaratnam

**Affiliations:** 1 Maidstone and Tunbridge Wells NHS Trust, Tunbridge Wells Hospital, Tonbridge Road, Pembury, Tunbridge Wells, Kent, TN2 4QJ UK; 2 Department of Health Services Research and Policy, London School of Hygiene and Tropical Medicine, 15-17 Tavistock Pl, London, WC1H 9SH UK; 3 UCL Department of Primary Care and Population Health, UCL Medical School, Upper Third Floor, Rowland Hill Street, London, NW3 2PF UK; 4 Northern Devon Healthcare NHS Trust, North Devon District Hospital, Raleigh Heights, Barnstaple, EX31 4JB UK

**Keywords:** Primary care, Family medicine, General practice, General practitioner, Family doctor, Family physician, Behaviour Change Wheel, Theoretical domains framework, COM-B, Behaviour change, Quality improvement

## Abstract

**Background:**

Understanding the barriers and facilitators to behaviour change by primary care practitioners (PCPs) is vital to inform the design and implementation of successful Behaviour Change Interventions (BCIs), embed evidence-based medicine into routine clinical practice, and improve quality of care and population health outcomes.

**Methods:**

A theory-led systematic review of reviews examining barriers and facilitators to clinical behaviour change by PCPs in high-income primary care contexts using PRISMA. Embase, MEDLINE, PsychInfo, HMIC and Cochrane Library were searched. Content and framework analysis was used to map reported barriers and facilitators to the Theoretical Domains Framework (TDF) and describe emergent themes. Intervention functions and policy categories to change behaviour associated with these domains were identified using the COM-B Model and Behaviour Change Wheel (BCW).

**Results:**

Four thousand three hundred eighty-eight reviews were identified. Nineteen were included. The average quality score was 7.5/11. Reviews infrequently used theory to structure their methods or interpret their findings. Barriers and facilitators most frequently identified as important were principally related to ‘*Knowledge*’, ‘*Environmental context and resources*’ and ‘*Social influences*’ TDF domains. These fall under the ‘Capability’ and ‘Opportunity’ domains of COM-B, and are linked with interventions related to education, training, restriction, environmental restructuring and enablement. From this, three key areas for policy change include guidelines, regulation and legislation. Factors least frequently identified as important were related to ‘Motivation’ and other psychological aspects of ‘Capability’ of COM-B. Based on this, BCW intervention functions of persuasion, incentivisation, coercion and modelling may be perceived as less relevant by PCPs to change behaviour.

**Conclusions:**

PCPs commonly perceive barriers and facilitators to behaviour change related to the ‘Capability’ and ‘Opportunity’ domains of COM-B. PCPs may lack insight into the role that ‘Motivation’ and aspects of psychological ‘Capability’ have in behaviour change and/or that research methods have been inadequate to capture their function. Future research should apply theory-based frameworks and appropriate design methods to explore these factors. With no ‘one size fits all’ intervention, these findings provide general, transferable insights into how to approach changing clinical behaviour by PCPs, based on their own views on the barriers and facilitators to behaviour change.

**Systematic review registration:**

A protocol was submitted to the London School of Hygiene and Tropical Medicine via the Ethics and CARE form submission on 16.4.2020, ref number 21478 (available on request). The project was not registered on PROSPERO.

**Supplementary Information:**

The online version contains supplementary material available at 10.1186/s13643-022-02030-2.

## Background

Known as the “second translational gap” [1], a gap in translation between evidence-based interventions and everyday clinical practice has been shown across different clinical areas and international settings [2–4], with numerous organisational and individual factors influencing clinical behaviour. Existing literature has shown that there is particularly wide variation in clinical behaviour in the primary care setting, which cannot be explained by case mix and clinical factors alone [5, 6]. This variation is of particular concern, as it is widely accepted that primary care is the cornerstone of a strong healthcare system [7], and stronger primary care systems are generally associated with better and more equitable population health outcomes [8–11]. With an ageing population and unique evolving challenges faced in primary care, understanding the contextual barriers and facilitators to successful behaviour change by primary care practitioners (PCPs) is vital to inform the design and implementation of successful behaviour change interventions (BCIs), and is likely to offer the greatest potential improvement in quality of care and population health outcomes.

### Behaviour change interventions

Changing behaviour of healthcare professionals is not easy, but has been shown to be easier when evidence-based theory informs intervention development [12]. BCIs aimed at healthcare professionals have traditionally been related to incentivisation schemes, guidelines, educational outreach, audit and feedback, printed materials and reminders [13, 14]. These have often emerged from approaches to understanding behaviour change, focused on individual attitude-intention processes [15] and theories emphasising self-interest [16, 17]. However, the impact of these interventions on changing clinicians’ behaviour has been found to variable [18]. Within the context of primary care, attitude-intention processes may not fully explain (lack of) behaviour change, where PCPs face competing pressures, such as caring for multiple patients with limited time, identifying pathology among undifferentiated symptoms, coping with emotional situations, managing uncertainty and keeping up-to-date with substantial volumes of new evidence. Similarly, theories of self-interest may not fully translate to PCPs. BCIs are often implemented through collective action across teams or based on financial levers [19–21]; however, the organisational context where PCPs work can vary from a single or group community-based practices with variable payment systems [22]. Therefore, while other healthcare professionals, patients and carers are likely to offer valuable insights, understanding PCPs’ own perspectives on the barriers and facilitators to behaviour change by PCPs is a vital starting point.

### Theoretical Domains Framework and Behaviour Change Wheel

**Fig. 1:**
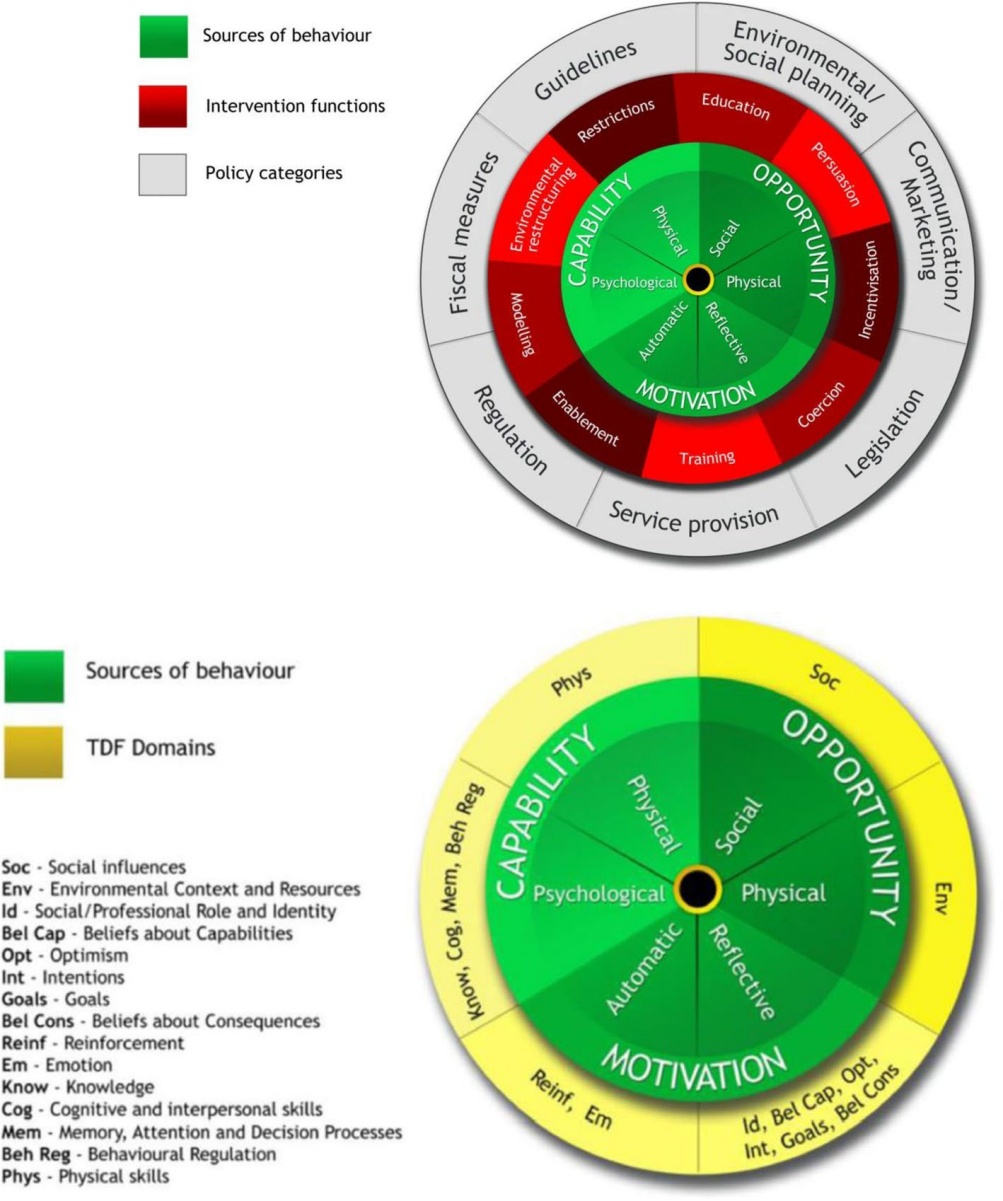
The Behaviour Change Wheel (BCW) [26] (above) and the relationship with the Theoretical Domains Framework (TDF) [25] (below)

The Theoretical Domains Framework (TDF) of behaviour change [23] simplifies and integrates 33 theories and 128 key theoretical constructs related to behaviour change into a single framework for use across multiple disciplines. Theoretical constructs are grouped into 14 domains in the final paper by Michie et al. [24], encompassing individual, social and environmental factors, with the majority relating to individual motivation and capability factors [25] (Fig. 1). Skills can be subcategorised into cognitive and interpersonal, and physical, although cognitive and inter-personal skills are more relevant to primary care (Table 1).

**Table 1:** Theoretical Domains Framework (TDF) of behaviour change domains and definitions [24]

COM-B		TDF domain	Definition
**Capability**	**Psychological**	**Knowledge**	An awareness of the existence of something.
		**Skills: cognitive and interpersonal**	An ability or proficiency acquired through practice.
		**Memory, attention and decision processes**	The ability to retain information, focus selectively on aspects of the environment and choose between two or more alternatives.
		**Behavioural regulation**	Anything aimed at managing or changing objectively observed or measured actions.
	**Physical**	**Skills: physical**	An ability or proficiency acquired through practice.
**Opportunity**	**Social**	**Social influences**	Those interpersonal processes that can cause individuals to change their thoughts, feelings or behaviours.
	**Physical**	**Environmental context and resources**	Any circumstance of a person’s situation or environment that discourages or encourages the development of skills and abilities, independence, social competence and adaptive behaviour.
**Motivation**	**Reflective**	**Social/professional role and identity**	A coherent set of behaviours and displayed personal qualities of an individual in a social or work setting.
		**Beliefs about capabilities**	Acceptance of the truth, reality or validity about an ability, talent or facility that a person can put to constructive use.
		**Optimism**	The confidence that things will happen for the best or that desired goals will be attained.
		**Intentions**	A conscious decision to perform a behaviour or a resolve to act in a certain way.
		**Goals**	Mental representations of outcomes or end states that an individual wants to achieve.
		**Beliefs about consequences**	Acceptance of the truth, reality, or validity about outcomes of a behaviour in a given situation.
	**Automatic**	**Reinforcement**	Increasing the probability of a response by arranging a dependent relationship, or contingency, between the response and a given stimulus.
		**Emotion**	A complex reaction pattern, involving experiential, behavioural, and physiological elements, by which the individual attempts to deal with a personally significant matter or event.

The TDF has been widely used to examine clinical behaviour change in healthcare settings [25, 27–34]. Key advantages of the TDF include a comprehensive range of domains useful for synthesising large amounts of data [24] and the domains can be used to identify the types of interventions and policy strategies necessary to change those mechanisms of behaviour, using the Behaviour Change Wheel (BCW) [26]. Developed by Michie et al., the BCW can be used to characterise interventions by their “functions” and link these to behavioural targets, categorised in terms of capability (individual capacity to engage in the activity concerned), opportunity (all the factors that lie outside the individual that make the behaviour possible or prompt it) and motivation (brain processes that energize and direct behaviour), known as the COM-B System. Capability encompasses not only individual physical capability, but also psychological capability, defined as the capacity to engage in the necessary thought processes using comprehension, reasoning etc. Strategies to modify behaviour can be identified based on salient TDF and COM-B domains [35].

### The evidence gap

Never having been done before, the aims of this systematic review of reviews were to:

1. Identify barriers and facilitators to clinical behaviour change by PCPs through the theoretical lenses of the TDF and BCW, from the perspective of PCPs.
2. Help inform the future development and implementation of theory-led BCIs, to embed EBM into routine clinical practice, improve quality of care and population health outcomes.

A systematic review of reviews was deemed an appropriate method to address these aims, as the literature is substantial and heterogeneous. Existing reviews of reviews have looked at different types of effective BCIs, both in primary care [36, 37] and in healthcare in general [18], however none have looked at barriers and facilitators to PCPs’ behaviour change, using both the TDF and BCW models as a theoretical basis.

We aimed to answer the following questions:

1. Which TDF domains are most frequently identified as important by PCPs when barriers and facilitators to clinical behaviour change by PCPs are mapped to the TDF framework?
2. What important themes emerge within these TDF domains?
3. What intervention functions and policy strategies from the COM-B Model and BCW link to these TDF domains, and what are the implication of this?

## Methods

Guidance presented in the Joanna Briggs Institute (JBI) Manual for Evidence Synthesis [38] was used as methodological guidance to conduct the review, which provides guidance for umbrella reviews synthesising qualitative and quantitative data on topics other than intervention effectiveness. This guidance, alongside a modified version of the Preferred Reporting Items for Systematic Reviews and Meta-Analyses (PRISMA) guidelines [39], were used for reporting (Additional file 1).

### Searches

A comprehensive database search strategy was devised by MM with assistance from a librarian from LSHTM. The search was conducted by MM on April 16th 2020 without date restriction, using the following databases: Embase (1947 to 2020 April 14), MEDLINE (1946 to April week 1 2020), PsychInfo (1806 to April week 1 2020), Health Management Information Consortium (HMIC) (1979 to March 2020) and Cochrane Library (inception to April 2020). The full search strategies are shown in Additional file 2.

In addition, grey literature was hand-searched by MM on the following websites: Public Health England [40], the University College London (UCL) Centre for Behaviour Change [41] and the National Institute for Health and Care Excellence (NICE) Evidence Search [42]. After screening and selection, reference lists of the included reviews were screened for additional relevant reviews.

### Inclusion and exclusion criteria

To be included, articles had to be reviews of qualitative, quantitative or mixed methods empirical studies examining barriers and facilitators to clinical behaviour change by PCPs. Inclusion and exclusion criteria were defined using the PICo framework (Population, phenomena of Interest, Context) [43], to enable transparency and reproducibility. The element of ‘types of studies’ was added to specify types of evidence included (Table 2).

**Table 2:** Inclusion and exclusion criteria using an adapted version of the PICo framework

	Inclusion criteria	Exclusion criteria
**Population**	PCPs (general practitioners/family doctors, physicians in community paediatrics, community obstetrics and gynaecology or general internal medicine)	• Participant roles unclear • PCP data not reported separately from data regarding non-PCPs, students, patients or carers
**Phenomena of interest**	Barriers and facilitators (directly reported by PCPs or extracted from views, perceptions, beliefs, attitudes and experiences of PCPs) to clinical behaviour change (any behaviour in relation to patient care, including diagnosis, management, communication with patients and shared-decision-making, and inter-professional collaboration). Barriers were defined as factors which obstruct or prevent clinical behaviour change. Facilitators were defined as factors which support or promote behaviour change.	Barriers and facilitators to: • patient or carer behaviour change • change in PCP knowledge or attitudes to a patient sub-group
**Context**	Majority high-income primary healthcare settings (as defined by The World Bank country classification [43])	• Non-primary healthcare settings • Majority low-middle-income settings
**Types of studies**	Any type of review (including but not limited to systematic, narrative, realist, meta-aggregation, meta-ethnography) examining qualitative, quantitative or mixed empirical studies in English	• Reviews in a language other than English • Reviews of reviews, abstracts, protocols, errata, editorials and conference reports

*PCPs* primary care practitioners

#### Rationale

##### Population

In most HIC settings, general practitioners/family doctors are the main providers of primary care, however often included a mix of PCPs (healthcare professionals working in primary care).

##### Context

PCPs usually provide the mainstay of care in high-income settings. Common barriers and facilitators across a wide range of high-income settings provides stronger evidence for context-specific recommendations.

Types of studies:

- The inclusion of all types of reviews (including but not limited to narrative and realist reviews, meta-ethnography and meta-aggregation) allows for a broader review of available literature and they are not bound by the specificity of systematic reviews [44, 45].
- Only reviews published in English were included.

### Screening and selection

Results from database searches were exported to EndNote X9 software and deduplicated. Titles and abstracts were screened independently by two reviewers (MM and SN). If the abstract contained insufficient information to determine eligibility, a copy of the full text was obtained. The full texts of articles meeting the inclusion criteria were obtained and reviewed. A standardised form including elements of the PICo framework was used at the full text review stage to identify relevant articles in a consistent way. Articles which could not be accessed online were obtained by contacting authors. Authors were also contacted to obtain clarification where eligibility was unclear. Reference lists of included articles were hand searched by MM and SN to identify additional relevant articles, subject to the same screening and selection processes described.

### Quality appraisal

The Joanna Briggs Institute (JBI) Critical Appraisal Checklist for Systematic Reviews and Research Syntheses [38] was used for quality appraisal, conducted independently by MM and SN. This tool was applicable to reviews of observational studies, which constituted the majority of the included articles; therefore, all reviews, regardless of their type, were subject to quality appraisal using the JBI checklist. This also allowed for consistency in scoring and easier comparison between the reviews. A scoring system was pre-defined using a small sample of five articles and guidance in the JBI Manual for Evidence Synthesis [38]. Some articles fulfilled some but not all of the criteria for each question, which was believed to be reasonable, therefore an additional ‘partial yes’ response was added to reflect this (Additional file 3). With a maximum score of 11, scores were used to indicate low (≤ 4 points), moderate (> 4 and < 8 points) and high (≥ 8 points) quality. As outlined by Pope and Mays [46], the value of specific pieces of qualitative research may only emerge during the synthesis process and may still offer valuable insights despite low quality. No articles were therefore excluded on the basis of low quality scores.

### Data extraction

The JBI Data Extraction Form for Reviews of Systematic Reviews and Research Syntheses [38] was adapted to extract relevant data from included reviews. A citation matrix was created to map the included empirical studies of each review and identify duplicate references.

### Data analysis and synthesis

Data analysis was conducted independently by MM and SN. Previously reported analysis methods [25, 47] were used to guide data analysis and synthesis methods. A combination of content and framework analysis was used, described in five steps:

1. Data extraction: full-text versions of the included articles were imported into NVivo software and data were extracted from results and discussion sections and supplementary files. Data included barriers, facilitators and factors which could be both barriers and facilitators.
2. Deductive analysis: extracted barriers, facilitators and factors were mapped to relevant TDF domains using component constructs of each domain, outlined by Cane et al. [24]. Almost all reported barriers and facilitators related to skills were cognitive and interpersonal, therefore the TDF domain ‘skills: physical’ was removed.
3. Counts were used to identify the most frequently-reported TDF domains. Owing to the vast amount of information across the included reviews, counts were also used to identify the TDF domains most frequently reported as important by authors. This was done in three ways: where authors explicitly stated they were important or salient, where they were most frequently reported where authors used frequency counts, and where authors highlighted or focused on them in the discussion section to draw main conclusions.
4. Inductive analysis: thematic analysis was conducted to identify emergent themes within the TDF domains most frequently identified as important to provide context to the role each barrier, facilitator and factor plays in hindering or facilitating clinical behaviour change. Owing to the vast amount of information across the included reviews, themes reported as important or salient by five or more reviews were labelled as important overall.
5. TDF domains most frequently identified as important were mapped to the COM-B model of the BCW to identify the associated intervention functions and policy categories.

Discrepancies between reviewers at the screening, selection, quality appraisal and analysis stages were discussed until a consensus was reached.

## Results

### Search results and selection

**Fig. 2:**
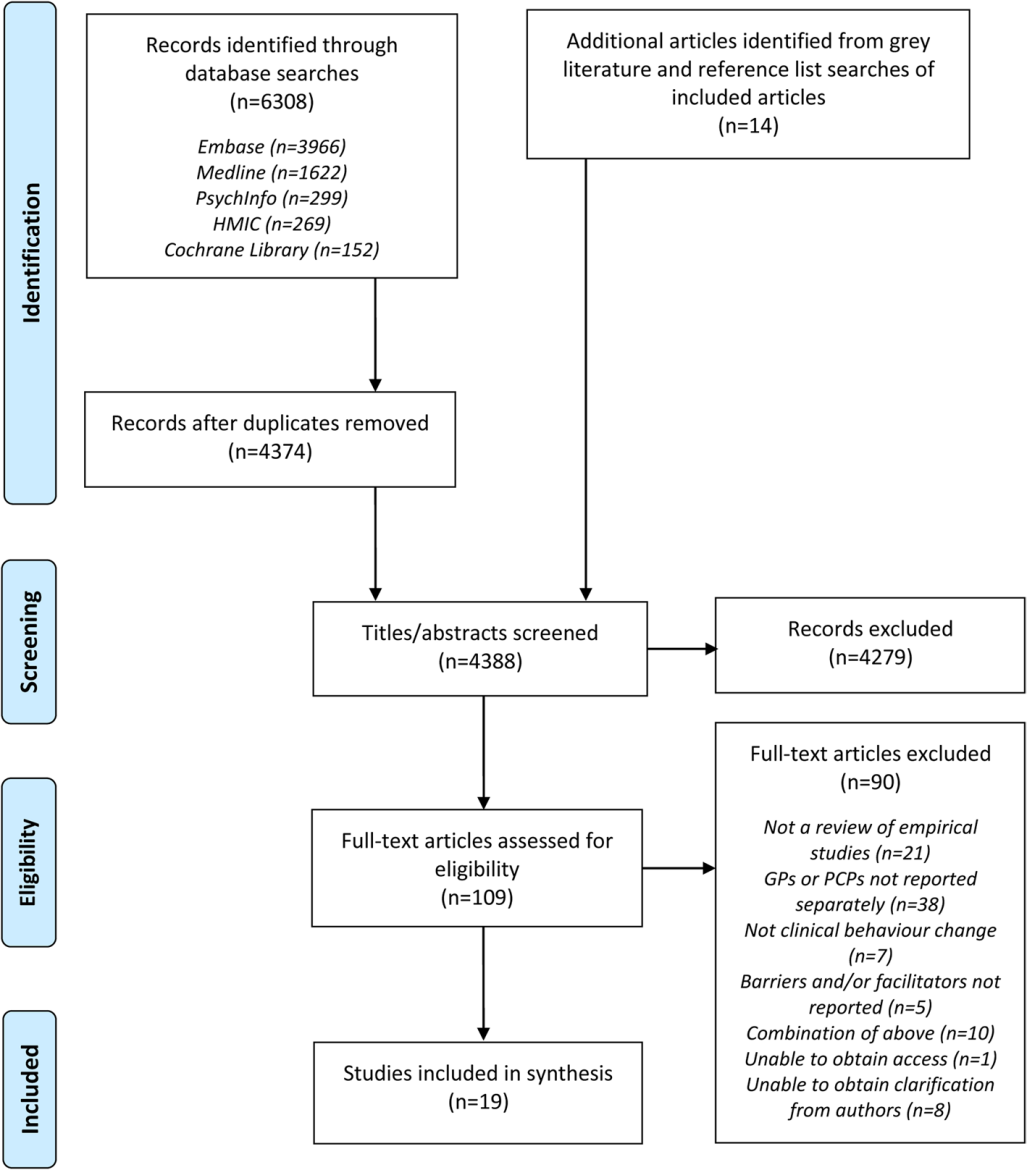
Flow chart [66] of review process

Database searches identified 6308 records. After duplicates were removed, there were 4374 records remaining. An additional 14 articles were identified from grey literature and reference list searches. The vast majority of these articles were either not a review of empirical studies, or they did not focus on behaviour change. Where they did focus on behaviour change, they focused on patient behaviour change, rather than that of PCPs. One hundred and nine full-text articles were assessed for eligibility. Clarification was sought from 19 authors on participant roles, search strategies and synthesis methods, and was obtained from 11 authors. Nineteen reviews [33, 48–65] were included in the data synthesis (Fig. 2).

### Characteristics of included reviews

Of the 19 included reviews, 17 [48–62, 64, 65] were systematic reviews and 2 [33, 63] were narrative reviews. The reviews were all published between 2005 and 2020. Four hundred and one empirical studies were included in total across a wide range of settings and healthcare systems. Almost all studies were conducted in high-income countries, the majority of which were conducted in Europe, USA, Canada, Australia and New Zealand. Five studies (1%) were conducted in upper-middle-income countries, including Jordan, Turkey, South Africa and Bosnia and Herzegovina. Seven reviews [48–53, 65] only included qualitative studies, two [54, 55] only included quantitative studies, and 10 [33, 56–64] included qualitative, quantitative and/or mixed methods studies. Most studies were observational, utilising qualitative interviews and/or focus groups, or cross-sectional surveys. A minority of observational studies from six [55, 58, 59, 62–64] reviews were part of larger intervention studies.

More than 72,000 PCPs were included in total. Seven [33, 48, 49, 52, 55, 63, 64] reviews only reported general practitioner (GP) or family physician (FP) data and 12 [50, 51, 53, 54, 56–62, 65] reported a mix of PCP data, the majority of which were GPs, FPs, and community paediatrics and obstetrics and gynaecology physicians. Of these, five reviews [51, 56, 60, 62, 65] included primary care non-physicians, including nurse practitioners (NPs) and physician assistants. Seven reviews [50, 52, 53, 56, 58, 61, 65] examined behaviour related to clinical management of a range of topics, of which three specifically examined prescribing behaviour; four reviews [33, 51, 59, 63] examined diagnostic processes; two [49, 54] examined prevention; two [55, 57] examined communication and engagement with patients; two [48, 64] examined the practice of EBM in general; one [62] examined collaborative practice; and one [60] examined service provision. Eighteen studies were referenced by two reviews each due to overlapping phenomena of interest. The most common data synthesis methods were thematic/narrative synthesis and meta-ethnography, used by 13 reviews [48–53, 55, 56, 58, 60–62, 65]. Six reviews [33, 54, 57, 59, 63, 64] used framework synthesis; only three reviews [33, 59, 64] used existing theoretical frameworks or models, such as the TDF and COM-B. A summary of review characteristics is shown in Table 3. Additional information is shown in Additional file 4.

**Table 3:** Characteristics of included reviews

First author (year)	Type of review	Phenomena of interest	Participants	Settings	Included studies	Synthesis methods	Quality score (0–11)
**Clinical management**							
^**a**^**Sinnott (2013)** [52]	SR	GPs’ perceptions on the clinical management of multimorbidity.	275 GPs	UK (3), Netherlands (2), USA (2), Belgium (1), Germany (1), Ireland (1)	**10 studies:** Qualitative	Meta-ethnography	**10.5**^**high**^
^**a**^**O’Brien (2016)** [61]	SR	PCPs’ perceptions of the barriers that prevent effective management of child and adolescent mental health problems.	9624 PCPs (FPs, GPs, paeds)	USA (22), UK (9), Australia (4), Canada (4), Ireland (2), South Africa (2), Malta (1), Puerto Rico (1)	**43 studies:** Qualitative and quantitative	Thematic synthesis	**9.5**^**high**^
**Barley (2011)** [56]	SR	Barriers to and facilitators for good depression management.	GPs (2738) PNs (476)	UK (17)	**17 studies:** Qualitative and quantitative	Meta-ethnography	**9**^**high**^
**Lucas (2015)** [50]	SR	Views, beliefs and attitudes that influence prescribing for acute childhood infection.	207 PCPs (FPs, GPs, paeds)	UK (7), Iceland (3), France (2), USA (2), Belgium (1), Norway (1), New Zealand (1), Poland (1), Spain (1)	**15 studies:** Qualitative	Meta-ethnography	**9**^**high**^
**Lawrence (2016)** [58]	SR	PCPs’ perspectives of their role within cancer care.	10941 PCPs (FPs, GPs, obs-gynae, paeds)	USA (10), Canada (9), Netherlands (4), Australia (3), Norway (3), France (2), UK (2), Germany (1), Italy (1), Ireland (1), Israel (1)	**35 studies:** Qualitative and quantitative	Thematic synthesis	**8.5**^**high**^
**Sirdifield (2013)** [53]	SR	Clinicians’ experiences and perceptions of benzodiazepine prescribing.	187 PCPs (FPs, GPs, obs-gynae, paeds)	UK (2), Australia (1), Belgium (1), Canada (1), Norway (1), Slovenia (1), USA (1)	**8 studies:** Qualitative	Thematic synthesis	**8.5**^**high**^
**Tonkin-Crine (2011)** [65]	SR	GPs’ attitudes and experiences of antibiotic prescribing for acute respiratory tract infections.	321 PCPs (GPs, nurse prescribers)	UK (5), Iceland (2), Norway (2), Belgium (1), France (1), New Zealand (1), Netherlands (1), Poland (1), Spain (1), USA (1)	**12 studies:** Qualitative	Meta-ethnography	**5.5**^**mod**^
**Diagnosis**							
**Schumann (2012)** [51]	SR	Family physicians’ perceived barriers to diagnosing depression.	239 PCPs (FPs, GIs, NPs)	UK (5), USA (3), Australia (1), Canada (1), Germany (1), Netherlands (1), Sweden (1)	**15 articles reporting 13 studies:** Qualitative	Thematic synthesis	**10.5**^**high**^
^**a**^**McDonagh (2018)** [59]	SR	Barriers and facilitators to chlamydia testing for young people and PCPs.	> 1733 PCPs	UK (15), Australia (9), Estonia (1), France (1), New Zealand (1), Sweden (1)	**25 studies:** Qualitative and quantitative	Framework synthesis (COM-B Model)	**7.5**^**mod**^
^**a**^**Yeung (2015)** [63]	NR	Barriers and facilitators to chlamydia testing.	> 11993 GPs	Not reported	**37 studies:** Qualitative and quantitative	Framework synthesis (patient, GP and general practice-level)	**3.5**^**low**^
**Ogeil (2020)** [33]	NR	Barriers to the assessment and diagnosis of insomnia.	> 127 GPs	USA (4), Australia (2), Germany (2), UK (2)	**10 studies:** Qualitative and quantitative	Framework synthesis (TDF Framework)	**3**^**low**^
**Communication and engagement with patients**							
^**a**^**De Vleminck (2013)** [57]	SR	Perceived factors hindering or facilitating GPs in engaging in advance care planning (ACP).	> 2149 PCPs (FPs, GPs, obs-gynae, paeds)	UK (4), USA (3), Australia (2), Netherlands (2), Belgium (1), Canada (1), Israel (1), Singapore (1)	**15 studies:** Qualitative and quantitative	Framework synthesis (initial emergent themes)	**9.5**^**high**^
**Vogt (2005)** [55]	SR	Family physicians’ beliefs and attitudes towards discussing smoking cessation with patients.	GPs (5475) FPs (1143) Response rate 37-95%.	UK (5), USA (5), Italy (2), Australia (1), Bosnia and Herzegovina (1), Canada (1), Finland (1), France (1), New Zealand (1), Norway (1), Sweden (1)	**20 studies:** Quantitative	Thematic synthesis and pooled proportions	**5.5**^**mod**^
**Implementation of EBM in general**							
**Zwolsman (2012)** [64]	SR	Barriers encountered by GPs in the practice of EBM.	2390 GPs	UK (7), Australia (5), Bahrain (1), Belgium (1), Canada (1), Germany (1), Jordan (1), Norway (1), Singapore (1), Spain (1), Turkey (1), USA (1)	**22 studies:** Qualitative and quantitative	Framework synthesis (conceptual model	**9**^**high**^
**Carlsen (2007)** [48]	SR	GPs’ attitudes to and experiences with clinical practice guidelines.	GPs (172) FPs (147)	UK (6), Canada (3), Netherlands (2), USA (1)	**12 studies:** Qualitative	Thematic synthesis	**5.5**^**mod**^
**Prevention**							
**Vedel (2011)** [54]	SR	Barriers and facilitators to breast and colorectal cancer screening of older adults.	11022 PCPs (FPs, GPs, obs-gynae, paeds) Response rate 21–93%.	USA (12), Canada (3), Australia (2), France (2), Italy (1), Switzerland (1)	**21 studies:** Quantitative	Framework synthesis (own taxonomy)	**8**^**high**^
**Ju (2018)** [49]	SR	GPs’ perspectives on the prevention of cardiovascular disease.	> 1223 GPs	Not reported	**34 studies:** Qualitative	Thematic synthesis	**7.5**^**mod**^
**Service provision**							
^**a**^**Mikat-Stevens (2015)** [60]	SR	Primary care providers’ perceived barriers against provision of genetics services.	> 8494 PCPs (FPs, GPs, obs-gynae, paeds, advanced practice nurses, PAs)	USA (17), UK (6), Australia (4), Canada (4), Netherlands (3), Germany (1), New Zealand (1), Switzerland (1)	**38 studies:** Qualitative and quantitative	Thematic synthesis	**7.5**^**mod**^
**Collaborative practice**							
**Schadewaldt (2013)** [62]	SR	Views and experiences of nurse practitioners and medical practitioners with collaborative practice.	PCPs (1641 medical practitioners, 380 NPs)	USA (11), Canada (6), UK (6), Ireland (1), Netherlands (1), New Zealand (1), Sweden (1)	**30 articles reporting 27 studies:** Qualitative and quantitative	Thematic synthesis	**5.5**^**mod**^

*CVD* cardiovascular disease, *FP* family physician, *GI* general internist, *GP* general practitioner, *NP* nurse practitioner, *NR* narrative review, *obs-gynae* obstetrics and gynaecology physician, *PA* physician assistant, *paeds* paediatric physician, *PN* practice nurse, *RCT* randomised controlled trial, *SR* systematic review

^Low^Low quality

^mod^Moderate quality

^high^High quality

^a^Clarification obtained from authors on participants and synthesis methods

### Quality appraisal

#### Quality of empirical studies

Three reviews [33, 55, 63] did not conduct quality appraisal, two of which [33, 63] were narrative reviews. The remaining reviews used a wide range of appraisal tools to suit the type of data they included, which were primarily existing tools in their original or adapted forms. Five reviews [56, 57, 60, 62, 64] used more than one tool. The most common appraisal tools used were CASP (Critical Appraisal Skills Programme) Checklists [67] for qualitative and quantitative research, used by seven reviews [48, 52, 53, 56, 57, 59, 65]. There was large variation in how authors used the appraisal tools, therefore quality of studies could not be reliably compared between reviews. Detailed information is shown in Additional file 3.

#### Quality of reviews

Ten reviews were of high quality, seven were of moderate-quality and two were of low quality using the JBI checklist. The highest score was 10.5/11 and the lowest was 3/11. The average score was 7.5/11, which is considered moderate quality. Reviews generally included well-evidenced recommendations for policy and practice and appropriate directives for future research. On validity, reviews scored highest on appropriate inclusion criteria for the review question, and appropriate methods used to combine studies. Reviews scored poorly on using an appropriate search strategy and assessing for publication bias. Most reviews did not justify search limits and/or did not provide evidence of a search strategy. Scores for each of the criteria are shown in Additional file 3.

### Main findings

A large number of barriers, facilitators and factors were identified by authors, often interacting with each other in a complex way (Table 4). As a result, some barriers and facilitators were mapped to more than one TDF domain. All TDF domains were identified. All reviews identified ‘environmental context and resources’ as important, and all but two reviews identified ‘knowledge’ and ‘social influences’ as important. TDF domains identified least frequently as important were ‘goals’, ‘intentions’ and ‘optimism’. Although ‘social/professional role and identity’, ‘skills’ and ‘emotion’ TDF domains were frequently identified, they were less frequently highlighted as important by authors. Table 4 shows how each TDF domain and COM-B domains were mapped to each of the included reviews, as well as which domains were identified as important.

**Table 4:** Barriers, facilitators and factors mapped to the Theoretical Domains Framework (TDF)

COM-B	Capability				Opportunity		Motivation							
	Psychological				Social	Physical	Reflective						Automatic	
TDF domains	Knowledge	Memory, attention and decision processes	Behavioural regulation	Skills: cognitive and inter-personal	Social influences	Environmental context and resources	Social/professional role and identity	Beliefs about capabilities	Optimism	Intentions	Goals	Beliefs about consequences	Reinforcement	Emotion
**Clinical management**														
**Sinnott (2013)**^**high**^ [52]		✓		✓			✓				✓	✓		✓
**O’Brien (2016)**^**high**^ [61]				✓			✓	✓					✓	✓
**Barley (2011)**^**high**^ [56]		✓						✓	✓					✓
**Lucas (2015)**^**high**^ [50]			✓	✓									✓	✓
**Lawrence (2016)**^**high**^ [58]			✓											✓
**Sirdifield (2013)**^**high**^ [53]			✓					✓	✓			✓	✓	✓
**Tonkin-Crine (2011)**^**mod**^ [65]												✓	✓	✓
**Diagnosis**														
**Schumann (2012)**^**high**^ [51]				✓				✓				✓		✓
**McDonagh (2018)**^**mod**^ [59]							✓							✓
**Yeung (2015)**^**low**^ [63]		✓	✓				✓		✓					✓
**Ogeil (2020)**^**low**^ [33]					✓		✓	✓						
**Communication and engagement with patients**														
**De Vleminck (2013)**^**high**^ [57]	✓		✓									✓	✓	✓
**Vogt (2005)**^**mod**^ [55]							✓							✓
**Implementation of EBM in general**														
**Zwolsman (2012)**^**high**^ [64]			✓				✓	✓					✓	
**Carlsen (2007)**^**mod**^ [48]				✓						✓				✓
**Prevention**														
**Vedel (2011)**^**high**^ [54]			✓	✓			✓						✓	
**Ju (2018)**^**mod**^ [49]	✓	✓							✓	✓	✓		✓	✓
**Service provision**														
**Mikat-Stevens (2015)**^**mod**^ [60]			✓					✓				✓		
**Collaborative practice**														
**Schadewaldt (2013)**^**mod**^ [62]											✓		✓	
**Total**	**19**	**9**	**10**	**16**	**18**	**19**	**18**	**12**	**5**	**2**	**3**	**12**	**12**	**16**
**Important**	**17**	**5**	**2**	**10**	**17**	**19**	**10**	**5**	**1**	**0**	**0**	**6**	**3**	**2**

Barriers, facilitators and factors identified as important or salient by authors are indicated by an encircled tick

^low^Low quality

^mod^Moderate quality

^high^High quality

Forty-two themes were identified in total across all TDF domains, 12 of which were labelled as important overall. A theme was labelled as important overall if five or more reviews identified it as important or salient. Within the ‘Knowledge’, ‘Environmental context and resources’, and ‘Social influences’ TDF domains, nine important themes emerged, of which the most frequently cited as important were ‘knowledge, awareness and uncertainty’ and ‘time, workload and general resources’. Across the remaining TDF domains, other important themes included ‘skills and competence’, ‘roles and responsibilities’, and ‘confidence in own ability’. Additional file 5 shows how themes were mapped to each TDF domain and each review, with corresponding quotes.

#### Capability: psychological (COM-B domain)

##### Knowledge (TDF domain)

**Knowledge, awareness and uncertainty (theme)**

Identified as important by 13 reviews [33, 50–52, 54, 55, 58–63, 65] (average quality score 7.2/11).

Inadequate knowledge and awareness and uncertainty were identified as important barriers to depression diagnosis and management [51, 56], recognition of insomnia [33], antibiotic prescribing in childhood infections [50] and acute respiratory tract infections (ARTIs) [65], engagement in cancer care [58], integration of genetics services [60], discussing smoking cessation [55], collaborative practice [62], management of multimorbidity [52], breast and colorectal screening in older adults [54] and chlamydia testing [59, 63]. This varied from a lack of knowledge of the topic as a whole, to more specific skills or outcomes. For example, PCPs reported a lack of knowledge around the epidemiology and presentation of chlamydia, benefits of testing, how to take specimens, and treatment options [59]. When prescribing antibiotics for childhood infections and ARTIs, PCPs reported they tend to prescribe “just in case” when they are uncertain of the consequences of not prescribing, such as when the diagnosis is unclear, or where there is no established doctor-patient relationship [50, 65]. There was widespread lack of knowledge within the field of genetics, including uncertainty around cancer genetics, genetic testing, genetic discrimination legislation, and local genetics service provision [60]. As well as a lack of knowledge of national guidelines and strategy [60, 63], inadequate guidelines were reported to exacerbate a lack of knowledge. For example, a lack of attention in guidelines on how social problems affect response to depression management was reported to exacerbate PCPs’ uncertainty around their role in managing depression [56]. Lack of knowledge and uncertainty were frequently reported to cause discomfort, low confidence, and reluctance to fill certain roles.

#### Opportunity: physical (COM-B domain)

##### Environmental context and resources (TDF domain)

**Time, workload and general resources (theme)**

Identified as important by 13 reviews [33, 48, 50, 51, 53–55, 58–61, 63, 64] (average quality score 7.3/11).

A lack of time to implement a variety of different tasks and clinical behaviours was reported, compounded by a large and complex workload and lack of general resources. A prominent barrier was time-pressured consultations, where PCPs reported difficulty in ensuring the clinician and parents are satisfied with the outcome when treating childhood infections [50], offering alternative interventions [53], listening to patients with depression [56], discussing emotions in cancer care [58] or smoking cessation with patients [55], introducing chlamydia testing and addressing sexual health-related concerns [63], recognising, diagnosing and managing child and adolescent mental health problems [61], and negotiating with patients [48]. PCPs also reported a lack of time to read and assess evidence and guidelines and reflect on their own practice [48, 64].

**Guidelines, evidence and decision-making tools (theme)**

Identified as important by five reviews [48, 52, 58, 64, 65] (average quality score 7.8/11).

Guidelines were a common factor reported to affect clinical behaviour, including a lack of guidelines/guidance, questionable evidence-base, and a disjunction between guidelines and personal experience. For example, PCPs reported difficulty in adapting recommendations to individual patient circumstances and practical constraints of the consultation [48, 51, 52]. PCPs felt that some guidelines lack the necessary flexibility when taking patient preferences and multi-morbidity into account, which can add to complexity and even cause harm in some cases [52, 64]. PCPs questioned the evidence-base of the guidelines due to low generalisability and narrow inclusion criteria of trials [48, 64] and potential biased sources of research, such as pharmaceutical companies [64]. The validity of criteria used for depression diagnosis was also questioned, with national guideline criteria defining depressive disorders using symptom counts, as opposed to viewing it as a syndrome requiring aetiological and conceptual thinking [51]. For non-English PCPs, access to evidence and guidelines in their native language was reported as a major barrier to implementing EBM [64].

**Financial resources and insurance coverage (theme)**

Identified as important by 6 reviews [49, 54, 58, 61, 62, 64] (average quality score 8/11).

Poor remuneration and increasing costs were common barriers reported in areas such as PCP involvement in cancer care [58], child and adolescent mental health [61] and use of EBM [64]. A major barrier to recognition and management of child and adolescent mental health problems and cancer in older adults was inadequate insurance coverage, including inadequate coverage of screening tests [54], restrictions on the number of funded therapy visits, and lack of psychiatrists [61]. As a result, increased reimbursement was identified as a potential facilitator that could increase child and adolescent mental health diagnoses [61].

**Education and training (theme)**

Identified as important by five reviews [33, 59, 63–65] (average quality score 5.7/11).

A lack of education and training was highlighted as an important barrier to chlamydia testing [59, 63], recognition of insomnia [33], antibiotic prescribing [65], and use of EBM [64]. PCPs reported that undergraduate sexual health teaching is inadequate [63] and that they have a lack of appropriate training and skills to discuss sexual health, take a sexual history, offer a test, manage treatment and notify partners. This has led to a reduction in knowledge and confidence to offer testing and discuss sexual health [59]. More education and training for PCPs and undergraduate students was frequently cited as a facilitator, as PCPs felt this would increase knowledge and confidence to change behaviour. Older male PCPs were identified as potentially in need of specific education on sexual health due to cultural differences with some patients receiving chlamydia testing [59]. Some PCPs reported that trustworthy and knowledgeable educational sources are important for PCPs to feel added value, with peer-led educational meetings given as an example [65].

#### Opportunity: social (COM-B domain)

##### Social influences (TDF domain)

**PCP-patient relationship and patient-centred care (theme)**

Identified as important by nine reviews [48, 49, 51–53, 57–59, 65] (average quality score 8.2/11).

Some PCPs reported that preservation of the PCP-patient relationship is prioritised over adherence to guidelines, particularly if guidelines recommend rationing services, or if PCPs feel empathetic towards anxious patients [48]. This dilemma was described as unpleasant and against the principles of patient-centred medicine, but sometimes necessary to avoid the potential litigation that rationing might bring [48] and loss of patients to other practices [53]. Similarly, the desire to maintain a good relationship is sometimes in competition with the PCP’s rationing role, leading some PCPs to give patients a “quick fix” when prescribing benzodiazepines [53]. Although not always reported as important, sensitive and emotive areas of medicine appear to be particularly affected, with PCPs reporting a concern for depriving patients of hope and/or damaging the relationship if they engage in the process of ACP [57], cancer care [58], or offer chlamydia testing [63]. Specifically, PCPs worried about appearing discriminatory and judgemental towards patients by offering chlamydia testing [63], and being too intrusive and paternalistic in recommending behaviour change to patients to prevent CVD [49]. This appears to be compounded by different religious and cultural norms between the PCP and patient, particularly if patients are of non-heteronormative orientation [63].

Establishing a rapport with patients and developing a long-standing, trusting doctor-patient relationship was identified as a facilitator for information-sharing, depression diagnosis [51], multimorbidity management [52], changing prescribing behaviour of benzodiazepines [53] and PCP engagement in ACP [57].

**Patient/carer characteristics (theme)**

Identified as important by eight reviews [49, 50, 53, 54, 56, 59, 64, 65] (average quality score 8/11).

The majority of reviews identified perceptions of patient/carer perceived ideas, concerns, expectations and motivations as important barriers to preventing CVD [49], prescribing antibiotics [50, 65] and benzodiazepines [53], chlamydia testing [59], cancer screening in older adults [54], and implementing EBM [64]. Attitudes were often born from stigma towards patients with mental health problems, and cultural diversity between the PCP and patient. For example, PCPs were found to have ambivalent attitudes towards working with depressed people, with some PCPs describing them as “burdens” and “people who bore you” [56]. Ethnic minorities were also felt to somatise their depression, and patients with social problems were seen to be avoiding work or seeking to medicalise their problems. This was compounded by a perception that management of patients presenting with social problems is complex. These beliefs were considered alongside other complex external factors, such as perceived pressure from parents to prescribe antibiotics [50], patient expectations different from the evidence [64], and a reluctance to medicalise unhealthy lifestyles [49].

**Collaboration and communication with other health professionals (theme)**

Identified as important by seven reviews [49, 52, 58, 59, 61, 62, 65] (average quality score 7.8/11).

Poor communication and uncoordinated care between PCPs and specialists were reported to hinder medication overviews, creating a feeling of uncertainty around the role of the PCP [52]. This was compounded by the perception of hierarchy between doctors and nurses [62] and negative attitudes towards handing over power [59]. Co-management with specialists was identified as an important facilitator in CVD prevention, to reinforce specialist advice and strengthen cohesive care [49]. Specialist input was desired by some PCPs to improve the awareness of the complexity of multimorbidity among specialists and ensure all doctors ‘speak with one voice’ to avoid provoking distrust [52]. Discussion with peers and personal or local prescribing feedback were identified as important facilitators to changing antibiotic prescribing [65]. Multiple facilitators to collaboration between nurse and medical practitioners in primary care were also identified [62]. These ranged from knowing the practitioner and having a good working relationship, reciprocity without hierarchy and control, effective communication including the use of technology, mutual trust and respect, shared responsibility and support from medical practitioners.

**Norms, stigma and attitudes (theme)**

Identified as important by five reviews [56, 59, 60, 63, 65] (average quality score 6.6/11).

The belief that patients would feel stigmatised or embarrassed was identified as an important barrier to depression diagnosis [56], chlamydia testing [59, 63], discussing family history and genetics [60] and antibiotic prescribing for ARTIs [65]. Stigma towards depression was seen as an important barrier to addressing psychosocial aspects of depression and commencing treatment amongst patients from the Caribbean and South Asia [56]. Stigmatising attitudes towards depressed, obese and elderly people was also reported to impact clinical decision-making [49, 56] (see ‘Patient and carer characteristics’ section). A major facilitator to reduce stigma and raise awareness was the normalisation of chlamydia testing [63]. This may include formal policy, guidelines or government programmes, feedback on testing rates, different methods of testing such as urine samples, and the use of non-heteronormative terminology.

#### BCW intervention functions and policy categories

COM-B components and intervention functions linked to the three TDF domains most frequently identified as important are shown in Table 5. Based on this, five intervention functions from the BCW were identified as most likely to be successful in changing clinical behaviour by PCPs. Associated with improving ‘capability’ are education (increasing knowledge or understanding), training (imparting skills) and enablement (promoting collective action across networks to overcome barriers, such as behavioural support for smoking cessation) interventions. Associated with improving social and physical ‘Opportunity’ are restriction (using rules to engage in the target behaviour), environmental restructuring (changing the physical or social context) and enablement interventions. The TDF domains ‘intentions’, ‘goals’ and ‘optimism’, which all map to the ‘motivation’ domain of the COM-B, were perceived as the least influential on clinical behaviour change by PCPs. As a result, BCW intervention functions including persuasion, incentivisation, coercion and modelling may be perceived as less relevant by PCPs to change behaviour.

**Table 5:** Behaviour Change Wheel (BCW) intervention functions and policy categories linked to the Theoretical Domains Framework (TDF) domains identified as most important by PCPs [26]

TDF domains	BCW COM-B components	BCW intervention functions associated with COM-B components				
		Education	Training	Restriction	Environmental restructuring	Enablement
Knowledge	Capability: psychological	✓	✓			✓
Environmental context and resources	Opportunity: physical			✓	✓	✓
Social influences	Opportunity: social			✓	✓	✓
**BCW policy categories associated with intervention functions**						
Communication/ marketing		✓				
Guidelines		✓	✓	✓	✓	✓
Fiscal measures			✓		✓	✓
Regulation		✓	✓	✓	✓	✓
Legislation		✓	✓	✓	✓	✓
Environmental/ social planning					✓	✓
Service provision		✓	✓			✓

Using the BCW, the three policy categories most commonly associated with supporting the delivery of the five intervention functions identified include guidelines (creating documents that recommend or mandate practice, including all changes to service provision), regulation (establishing rules or principles of behaviour or practice, such as establishing voluntary agreements on advertising), and legislation (making or changing laws, such as prohibiting sale or use) (Table 5).

## Discussion

### Summary of main results

Evidence across all reviews was heterogeneous, examining 16 different clinical behaviours across a range of primary care settings and healthcare systems. Most reviews were of moderate-to-high quality. All themes identified from the included reviews could be mapped to at least one domain from the TDF. Barriers, facilitators and factors most commonly reported by PCPs were related to ‘knowledge’, ‘environmental context and resources’ and ‘social influences’. Within these domains, ‘knowledge, awareness and uncertainty’ and ‘time, workload and general resources’ were by far the most important themes. Not only did factors affect various clinical behaviours such as diagnosis, management, and communication and collaboration with patients and other healthcare professionals, factors were also linked to each other in a complex way, often exacerbating each other in specific contexts and circumstances. For example, a lack of knowledge and uncertainty amongst PCPs is exacerbated by a poor or unestablished PCP-patient relationship, lack of time and resources, as well as patient characteristics, such as comorbidities and social problems.

Five out of nine intervention functions from the BCW (education, training, restriction, environmental restructuring and enablement) can be linked to the three TDF domains reported as most important by PCPs to help change clinical behaviour. These can be delivered through all seven policy categories of the BCW, although those most frequently associated policy categories with all five intervention categories are guidelines, regulation and legislation.

The TDF domains ‘intentions’, ‘goals’ and ‘optimism’, which all map to the ‘motivation’ domain of the COM-B, were perceived as the least influential on clinical behaviour change by PCPs. The TDF domains ‘behavioural regulation’, ‘memory, attention and decision processes’, ‘emotion’, ‘beliefs about consequences’, ‘reinforcement’, and ‘beliefs about capabilities’ were also perceived by PCPs as less important barriers or facilitators to behaviour change. ‘Behavioural regulation’, and ‘memory, attention and decision processes’ relate to the psychological aspect of ‘capability’ and the others, again, relate to the ‘Motivation’ domain of COM-B. This is a surprising finding, as the central premise of the TDF model is that domains linked to all three areas of the COM-B (capability, opportunity and motivation) model should interact to produce behaviour [25].

Linked to the automatic and reflective ‘motivation’ domain of COM-B are BCW interventions related to incentivisation, persuasion, coercion and modelling. It is therefore also surprising to find that PCPs did not identify these as important barriers and/or facilitators as substantial evidence exists regarding the widespread use of interventions associated with incentives (e.g. financial pay for performance or reputational league tables—albeit with mixed effects, and those which may utilise persuasion, modelling and even coercion (e.g. peer-to-peer outreach or public reporting) to change aspects of PCP behaviour [14, 68–70].

The limited frequency and importance given to aspects of psychological ‘Capability’ and ‘Motivation’ raises questions as to whether PCPs may have less insight into these areas or less desire to identify them as barriers or facilitator. It is possible they may be neglecting the role of brain processes involved in developing psychological capabilities, i.e. the capacity to engage in the necessary thought processes using comprehension and reasoning, and those that energize or direct behaviour, such as habitual processes, emotional responding and automatic decision-making. With most studies using qualitative interviews or cross-sectional surveys, questions may also have focused on domains researchers and BCI designers believed to be relevant, such as external factors including time, guidelines and patients.

### Key policy implications

Based on our findings, three TDF domains were most commonly reported across the majority of reviews, regardless of the type of behaviour change and context. This suggests that addressing these common factors through associated BCW intervention functions of education, training, restriction, environmental restructuring and enablement, and applying associated policy categories—namely guidelines, regulation and legislation, if not already addressed, could be prioritised to encourage PCPs to change clinical practice where needed across most clinical behaviours and settings.

### Strengths and limitations

The robustness of our findings is supported by several features. A broad, sensitive search strategy maximised the number of eligible reviews identified. Although the extent to which findings are applicable to a specific healthcare system or clinical context is unclear, reviews meeting the inclusion criteria focused on 16 types of clinical behaviours across a breadth of healthcare systems and included over 72,000 PCPs, providing a good starting point to identify commonalities across PCPs from a variety of different primary care settings.

Large amounts of heterogeneous data was summarised in a clear way using two evidence-based frameworks, however precise mapping of barriers, facilitators and factors to the TDF proved challenging, owing to the complex interplay between factors and interpretation of the authors of where they fitted. The integration of the TDF and BCW means important barriers and facilitators can be linked to practical strategies to address them, which does, however, rely on the validity of the frameworks themselves.

### Future research

Only a minority of reviews utilised a theory-based framework to synthesise evidence. To maximise the likelihood of intervention success and encourage the use of common terminology and understanding, future research should synthesise evidence using theory-informed frameworks, such as the TDF, paying particular attention to barriers and facilitators to behaviour change associated with PCPs’ own automatic and reflective motivation, and other aspects of psychological capability related to behaviour change. Methods exploring PCP motivation and aspects of psychological capability, as well as methods less reliant on PCPs’ insight, such as direct observation, may provide more valid conclusions.

## Conclusions

To the best of our knowledge, this is the first theory-led systematic review of reviews examining barriers and facilitators to clinical behaviour change by PCPs across a variety of primary care settings using the TDF and BCW. From the evidence available, PCPs perceive that factors related to knowledge, environmental context and resources and social influences are influential across a variety of primary care contexts, often interacting with each other in a complex way. It is vital that future research utilises theory-based frameworks and appropriate design methods to explore factors relating to automatic and reflective motivation, such as habitual processes, emotional responding and automatic decision-making that energize or direct behaviour, as well as psychological capability of PCPs, including the capacity to engage in the necessary thought processes using comprehension, reasoning etc. With no ‘one size fits all’ intervention, these findings go some way to offering general, transferable lessons in how to approach changing clinical behaviour by PCPs and improve quality of care and population health outcomes.

## Supplementary Information

**Additional file 1.** PRISMA checklist.

**Additional file 2.** Search strategy. Search concepts, keywords and MeSH terms used to derive search strategies. Search strategy.

**Additional file 3.** Quality appraisal. Adapted scoring system for the Joanna Briggs Institute (JBI) Critical Appraisal Checklist for Systematic Reviews and Research Syntheses. Quality of empirical studies: appraisal instruments and quality scores. Quality appraisal criteria.

**Additional file 4.** Additional data. Characteristics of included reviews.

**Additional file 5.** Evidence mapping. Mapping of emergent themes to the Theoretical Domains Framework (TDF). Evidence table.

## Data Availability

All data analysed during this study are included in this published article and its additional information files.

## Abbreviations

ARTI: Acute respiratory tract infection
BCI: Behaviour Change Intervention
BCW: Behaviour Change Wheel
CASP: Critical Appraisal Skills Programme
COM-B: Capability Opportunity Motivation Behaviour
CVD: Cardiovascular disease
EBM: Evidence-based medicine
FP: Family physician
GP: General practitioner
HMIC: Health Management Information Consortium
JBI: Joanna Briggs Institute
NICE: National Institute for Health and Care Excellence
NP: Nurse practitioner
PCP: Primary care practitioner
PRISMA: Preferred Reporting Items for Systematic Reviews and Meta-Analyses
TDF: Theoretical Domains Framework
