# Supplementary material for: Barriers and facilitators to clinical behaviour change by primary care practitioners: a theory-informed systematic review of reviews using the Theoretical Domains Framework and Behaviour Change Wheel
Source: Syst Rev. 2022 Aug 30;11:180. doi: 10.1186/s13643-022-02030-2 (PMC9429279; doi:10.1186/s13643-022-02030-2)
Supplement: Supplementary file 2 — Additional file 2. Search strategy. Search concepts, keywords and MeSH terms used to derive search strategies. Search strategy. [file 13643_2022_2030_MOESM2_ESM.docx]

**Supplementary File 1**

**Search concepts, keywords and MeSH terms used to derive search strategies**

| **Search concepts** | | | |
| --- | --- | --- | --- |
| **Primary care practitioner** | | **Systematic review** | |
| Keywords | **MeSH terms** | **Keywords** | **MeSH terms** |
| family doctor | general practitioner(s) | systematic review | systematic review(s) |
| family physician | general practice | meta-analysis |  |
| family practitioner | family physicians | narrative review |  |
| general practitioner | primary care | narrative synthesis |  |
| primary care doctor | physicians | realist review |  |
| primary care physician |  | realist synthesis |  |

**Search strategy**

**Embase Classic+Embase <1947 to 2020 April 14>**

|  | **Search** | **Results** |
| --- | --- | --- |
| 1 | exp general practitioner/ | 101889 |
| 2 | exp general practice/ | 84448 |
| 3 | family doctor*.ab,dm,dv,fx,hw,kw,mf,ot,ti,tn,dq. | 7129 |
| 4 | family physician*.ab,dm,dv,fx,hw,kw,mf,ot,ti,tn,dq. | 18968 |
| 5 | family practi*.ab,dm,dv,fx,hw,kw,mf,ot,ti,tn,dq. | 12730 |
| 6 | general practi*.ab,dm,dv,fx,hw,kw,mf,ot,ti,tn,dq. | 207307 |
| 7 | primary-care doctor*.ab,dm,dv,fx,hw,kw,mf,ot,ti,tn,dq. | 1580 |
| 8 | primary-care physician*.ab,dm,dv,fx,hw,kw,mf,ot,ti,tn,dq. | 27047 |
| 9 | 1 or 2 or 3 or 4 or 6 or 7 or 8 | 231188 |
| 10 | exp systematic review/ | 240454 |
| 11 | systematic review.ab,dm,dv,fx,hw,kw,mf,ot,ti,tn,dq. | 308068 |
| 12 | meta-analysis.ab,dm,dv,fx,hw,kw,mf,ot,ti,tn,dq. | 276270 |
| 13 | narrative review.ab,dm,dv,fx,hw,kw,mf,ot,ti,tn,dq. | 10137 |
| 14 | narrative synthesis.ab,dm,dv,fx,hw,kw,mf,ot,ti,tn,dq. | 2978 |
| 15 | realist review.ab,dm,dv,fx,hw,kw,mf,ot,ti,tn,dq. | 340 |
| 16 | realist synthesis.ab,dm,dv,fx,hw,kw,mf,ot,ti,tn,dq. | 214 |
| 17 | 10 or 11 or 12 or 13 or 14 or 15 or 16 | 454822 |
| 18 | 9 and 17 | 3966 |

**Ovid MEDLINE(R) <1946 to April Week 1 2020>**

|  | **Search** | **Results** |
| --- | --- | --- |
| 1 | exp general practitioners/ or exp physicians, family/ or exp physicians, primary care/ | 27121 |
| 2 | exp General Practice/ | 74617 |
| 3 | family doctor*.ab,hw,kf,ot,sy,ti,fx,nm,ox,px,rx,ui. | 4157 |
| 4 | family physician*.ab,hw,kf,ot,sy,ti,fx,nm,ox,px,rx,ui. | 12468 |
| 5 | family practi*.ab,hw,kf,ot,sy,ti,fx,nm,ox,px,rx,ui. | 68642 |
| 6 | general practi*.ab,hw,kf,ot,sy,ti,fx,nm,ox,px,rx,ui. | 83222 |
| 7 | primary-care doctor*.ab,hw,kf,ot,sy,ti,fx,nm,ox,px,rx,ui. | 882 |
| 8 | primary-care physician*.ab,hw,kf,ot,sy,ti,fx,nm,ox,px,rx,ui. | 17166 |
| 9 | 1 or 2 or 3 or 4 or 6 or 7 or 8 | 153232 |
| 10 | exp systematic review/ | 124189 |
| 11 | systematic review.ab,hw,kf,ot,sy,ti,fx,nm,ox,px,rx,ui. | 137362 |
| 12 | meta-analysis.ab,hw,kf,ot,sy,ti,fx,nm,ox,px,rx,ui. | 152522 |
| 13 | narrative review.ab,hw,kf,ot,sy,ti,fx,nm,ox,px,rx,ui. | 6306 |
| 14 | narrative synthesis.ab,hw,kf,ot,sy,ti,fx,nm,ox,px,rx,ui. | 1910 |
| 15 | realist review.ab,hw,kf,ot,sy,ti,fx,nm,ox,px,rx,ui. | 234 |
| 16 | realist synthesis.ab,hw,kf,ot,sy,ti,fx,nm,ox,px,rx,ui. | 163 |
| 17 | 10 or 11 or 12 or 13 or 14 or 15 or 16 | 232945 |
| 18 | 9 and 17 | 1622 |

**PsycInfo <1806 to April Week 1 2020>**

|  | **Search** | **Results** |
| --- | --- | --- |
| 1 | exp general practitioners/ | 5826 |
| 2 | family doctor*.ab,hw,id,mh,ot,tc,ti,tm. | 746 |
| 3 | family physician*.ab,hw,id,mh,ot,tc,ti,tm. | 3077 |
| 4 | family practi*.ab,hw,id,mh,ot,tc,ti,tm. | 6552 |
| 5 | general practi*.ab,hw,id,mh,ot,tc,ti,tm. | 15115 |
| 6 | primary-care doctor*.ab,hw,id,mh,ot,tc,ti,tm. | 227 |
| 7 | primary-care physician*.ab,hw,id,mh,ot,tc,ti,tm. | 4552 |
| 8 | 1 or 2 or 3 or 4 or 6 or 7 | 22635 |
| 9 | exp systematic review/ | 298 |
| 10 | systematic review.ab,hw,id,mh,ot,tc,ti,tm. | 27034 |
| 11 | meta-analysis.ab,hw,id,mh,ot,tc,ti,tm. | 29893 |
| 12 | narrative review.ab,hw,id,mh,ot,tc,ti,tm. | 2057 |
| 13 | narrative synthesis.ab,hw,id,mh,ot,tc,ti,tm. | 781 |
| 14 | realist review.ab,hw,id,mh,ot,tc,ti,tm. | 76 |
| 15 | realist synthesis.ab,hw,id,mh,ot,tc,ti,tm. | 83 |
| 16 | 9 or 10 or 11 or 12 or 13 or 14 or 15 | 51875 |
| 17 | 8 and 16 | 299 |

**HMIC Health Management Information Consortium <1979 to March 2020>**

|  | **Search** | **Results** |
| --- | --- | --- |
| 1 | exp general practice/ | 9114 |
| 2 | exp general practitioners/ | 10243 |
| 3 | family doctor*.ab,ot,hw,ti. | 385 |
| 4 | family physician*.ab,ot,hw,ti. | 265 |
| 5 | family practi*.ab,ot,hw,ti. | 1146 |
| 6 | general practi*.ab,ot,hw,ti. | 26447 |
| 7 | primary-care doctor*.ab,ot,hw,ti. | 93 |
| 8 | primary-care physician*.ab,ot,hw,ti. | 483 |
| 9 | 1 or 2 or 3 or 4 or 6 or 7 or 8 | 27739 |
| 10 | exp systematic reviews/ | 3119 |
| 11 | systematic review.ab,ot,hw,ti. | 3495 |
| 12 | meta-analysis.ab,ot,hw,ti. | 1553 |
| 13 | narrative review.ab,ot,hw,ti. | 176 |
| 14 | narrative synthesis.ab,ot,hw,ti. | 153 |
| 15 | realist review.ab,ot,hw,ti. | 47 |
| 16 | realist synthesis.ab,ot,hw,ti. | 34 |
| 17 | 10 or 11 or 12 or 13 or 14 or 15 or 16 | 5135 |
| 18 | 9 and 17 | 269 |

**Cochrane Library**

|  | **Search** | **Results** |
| --- | --- | --- |
| 1 | MeSH descriptor: [Physicians, Family] explode all trees | 449 |
| 2 | MeSH descriptor: [General Practitioners] explode all trees | 259 |
| 3 | MeSH descriptor: [Physicians, Primary Care] explode all trees | 147 |
| 4 | family doctor*:ti,ab,kw (Word variations have been searched) | 337 |
| 5 | family physician*:ti,ab,kw (Word variations have been searched) | 1012 |
| 6 | family practitioner*:ti,ab,kw (Word variations have been searched) | 94 |
| 7 | general practitioner*:ti,ab,kw (Word variations have been searched) | 6753 |
| 8 | primary care doctor*:ti,ab,kw (Word variations have been searched) | 101 |
| 9 | primary care physician*:ti,ab,kw (Word variations have been searched) | 2044 |
| 10 | family practice"):ti,ab,kw (Word variations have been searched) | 2415 |
| 11 | general practice:ti,ab,kw (Word variations have been searched) | 6001 |
| 12 | #1 or #2 or #3 or #4 or #5 or #6 or #7 or #8 or #9 or #10 or #11 in Cochrane Reviews | 152 |
