# Supplementary material for: Barriers and facilitators to clinical behaviour change by primary care practitioners: a theory-informed systematic review of reviews using the Theoretical Domains Framework and Behaviour Change Wheel
Source: Syst Rev. 2022 Aug 30;11:180. doi: 10.1186/s13643-022-02030-2 (PMC9429279; doi:10.1186/s13643-022-02030-2)
Supplement: Supplementary file 3 — Additional file 3. Quality appraisal. Adapted scoring system for the Joanna Briggs Institute (JBI) Critical Appraisal Checklist for Systematic Reviews and Research Syntheses. Quality of empirical studies: appraisal instruments and quality scores. Quality appraisal criteria. [file 13643_2022_2030_MOESM3_ESM.docx]

**Additional File 3**

**Adapted scoring system for the Joanna Briggs Institute (JBI) Critical Appraisal Checklist for Systematic Reviews and Research Syntheses**

| **Criteria** | **Yes = 1 point** |
| --- | --- |
| Is the review question clearly and explicitly stated? | All of the following elements of the PICo Framework:   1. Population 2. Phenomena of interest 3. Context |
| Were the inclusion criteria appropriate for the review question? | All of the following:   1. The inclusion criteria are identifiable from and match the review question. 2. PICo elements are explicitly and clearly defined. 3. Included studies are clearly eligible when matched against the stated inclusion criteria. 4. The types of included studies are relevant to the review question. |
| Was the search strategy appropriate?  *Papers were given a ‘partial yes’ (0.5 points) if they fulfilled criteria 1. and 3.** | All of the following:   1. Evidence of the search strategy is provided, either in the methods section or as an appendix. 2. A clear search strategy addresses each of the PICo components of the review question. 3. Logical and relevant keywords and terms are provided, with evidence that Subject Headings and Indexing terms have been used in the conduct of the search. 4. Search limits are justified and their potential impact is considered e.g. if a date limit was used, was this appropriate and/or justified? If only English language studies were included, will such a language bias have an impact on the review? |
| Were the sources and resources used to search for studies adequate?  *Papers were given a ‘partial yes’ (0.5 points) if they fulfilled criterion 1. only*** | Both of the following:   1. At least one electronic database searched including major bibliographic citation databases such as MEDLINE and CINAHL. Ideally, other databases that are relevant to the review question are also searched. 2. Grey literature or “unpublished” studies search attempted; this may involve searching websites relevant to the review question, or thesis repositories. |
| Were the criteria for appraising studies appropriate? | All of the following:   1. A clear statement that critical appraisal was conducted is made. 2. Details of the items that were used to assess the included studies is provided in the methods, as an appendix, or as a reference to a source that can be located. 3. The tools or instruments used are appropriate for the review question asked and the type of research conducted. |
| Was critical appraisal conducted by two or more reviewers independently? | A clear statement is made that critical appraisal was conducted by at least two reviewers working independently from each other and conferring where necessary to reach a decision regarding study quality and eligibility on the basis of quality. |
| Were there methods to minimise errors in data extraction? | Either of the following:   1. Data extraction is conducted in duplicate and independently. 2. Use of specific tools or instruments to guide data extraction and some evidence of piloting or training around their use, where applicable. |
| Were the methods used to combine studies appropriate? | All of the following:   1. The synthesis is appropriate for the review question, the stated type of systematic review, and evidence it refers to. Often, where heterogeneous studies are included, narrative synthesis will be an appropriate synthesis method. 2. If a qualitative review, the synthesis methods are congruent with the stated methodology 3. Adequate descriptive and explanatory information is given to support the final synthesized findings. |
| Was the likelihood of publication bias assessed? | Any of the following: ***   1. A comprehensive search strategy is used, including grey literature searches. 2. Egger’s test or funnel plots of quantitative evidence are presented. 3. Discussion around publication bias of quantitative or qualitative evidence. |
| Were recommendations for policy and/or practice supported by the reported data?  *Papers were given a ‘partial yes’ (0.5 points) if they fulfilled criterion 1. only***** | Both of the following:   1. A clear link between policy and/or practice recommendations and the results of the review is given. 2. Evidence that the strength of the findings and the quality of the research have been considered in the recommendations is provided. |
| Were the directives for new research appropriate? | Discussion of appropriate future research is given, based on either of the following:   1. Identified gaps in the research or knowledge base around a topic. 2. Where evidence is scarce or sample sizes are small and effect estimates are imprecise. |

##

*** A ‘partial yes’ was awarded if authors provided evidence of the search strategy with logical components, yet the search strategy did not include all elements of the PICo framework.

*Rationale:* Some articles chose to use a broad search strategy including some but not all of the PICo components.

**** A ‘partial yes’ was awarded if authors searched at least one electronic database, but did not include a search of grey literature.

*Rationale:* Some articles did not search grey literature, which was felt to reduce the validity somewhat, but search strategies using multiple electronic databases were still appropriate.

*** JBI guidance states that this question should be answered ‘not applicable’ for qualitative reviews. However, publication bias may be introduced in qualitative research. Unlike quantitative research where Egger’s tests and funnel plots can be used, there is no current way to assess publication bias in qualitative research, therefore ‘yes’ was awarded if publication bias was mentioned by the authors.

***** A* ‘partial yes’ was awarded if authors made a clear link between policy and/or practice recommendations and the results of the review, but did not consider the strength of the findings and quality of research.

*Rationale:* The majority of articles translated the results of the review to recommendations, however did not explicitly link quality of research to these recommendations. These recommendations were often felt to be appropriate, despite no explicit link to quality being made in the reporting.

**Quality of empirical studies: appraisal instruments and quality scores**

| **First author (year)** | **Quality appraisal instrument(s) used by authors of the included reviews** | **Empirical study quality scores of the included reviews** |
| --- | --- | --- |
| Sinnott (2013)^53^ | CASP checklist for qualitative research | Scoring system not used. The overall quality was high. |
| O’Brien (2016)^62^ | Adapted checklist from Kmet et al. | •Qualitative: high-quality (6), medium quality (4), low quality (3)  •Quantitative: high quality (17), medium quality (10), low quality (3)  •No studies excluded due to low quality |
| Barley (2011)^57^ | •CASP checklist for qualitative research  •Adapted tool for quantitative research | •Qualitative: 5 studies scored ≥7/10. 2 studies scored 5/10  •Quantitative: most were low quality (≤4/7)  •Quality appraisal not used to exclude studies. |
| Lucas (2015)^51^ | Checklist from Popay et al. | Scoring system not used. No studies excluded due to low quality. |
| Lawrence (2016)^59^ | Checklist adapted from Atkins et al. | Scoring system not used.  No studies excluded due to low quality. |
| Sirdifield (2013)^54^ | CASP checklist for qualitative research | All studies scored 9 or 10/10. |
| Tonkin-Crine (2011)^66^ | Adapted CASP checklist for qualitative research | No scores provided. No studies excluded due to low quality. |
| Schumann (2012)^52^ | Devised from Boulton et al, Cobb and Hagemaster and Pope and Mays | Scoring system not used. Ten studies met at least 8 of the 10 criteria. No studies excluded due to poor quality. |
| McDonagh (2018)^60^ | CASP checklists (not specified) | Scoring system not used. All studies except 3 were methodologically sound. Quality appraisal not used to exclude studies. |
| Ogeil (2020)^33^ | Quality not assessed | N/A |
| Yeung (2015)^64^ | Quality not assessed | N/A |
| De Vleminck (2013)^58^ | •CASP checklist for qualitative research  •Crombie critical appraisal tool for surveys | •Qualitative: 4 high-quality (≥8/10), 4 medium-quality (6-8/10)  •Quantitative: 5 high-quality (≥8/10), 2 medium-quality (6-8/10) |
| Vogt (2005)^56^ | Quality not assessed. | N/A |
| Zwolsman (2012)^65^ | •Quantitative studies: adapted recommendations from the STROBE initiative  • Qualitative studies: adapted criteria  proposed by Giacomini and Cook  • RCTs: criteria recommended by the Cochrane  Collaboration | •Positive and negative scores applied.  •Qualitative: 5 low-risk of bias  •Quantitative: 2 positive scores, 10 had up to 6 items that were inadequate, 1 negative score  •Mixed methods: high-risk of bias  •No studies excluded due to low quality. |
| Carlsen (2007)^49^ | Adapted CASP checklist for qualitative research | Scores not reported.  5 studies excluded due to low quality. |
| Vedel (2011)^55^ | 15 criteria from SUMARI, STROBE and MOOSE | Scoring system not used. No studies excluded due to low quality |
| Ju (2018)^50^ | COREQ | Score range 6-19/24.  No studies excluded due to low quality. |
| Mikat-Stevens (2015)^61^ | •Qualitative research tool used by Tong et al.  •Own bias score for quantitative research | •Qualitative: range 9-23/28, average 13/28  •Quantitative: range 0-2/2, average 0.88/2  •No studies excluded due to low quality. |
| Schadewaldt (2013)^63^ | •Descriptive/cross-sectional studies: 11 Questions to help you  make sense of descriptive/cross-sectional studies  •Quantitative surveys: CEBMA Appraisal Questions for a Survey  •Qualitative: JBI QARI  •Mixed methods research: Scoring System for appraising mixed methods research | Scoring system not used. Overall, studies were of moderate quality. No studies excluded due to low quality. |

**Quality appraisal criteria**

| **JBI Critical**  **Appraisal Checklist** | **Validity** | | | | | | | | | **Quality** | | **Score (0-11)** |
| --- | --- | --- | --- | --- | --- | --- | --- | --- | --- | --- | --- | --- |
|  | *Is the review question clearly and explicitly stated?* | *Were the inclusion criteria appropriate for the review question?* | *Was the search strategy appropriate?* | *Were the sources and resources used to search for studies adequate?* | *Were the criteria for appraising studies appropriate?* | *Was critical appraisal conducted by two or more reviewers independently?* | *Were there methods to minimise errors in data extraction?* | *Were the methods used to combine studies appropriate?* | *Was the likelihood of publication bias assessed?* | *Were recommendations for policy and/or practice supported by the reported data?* | *Were the directives for new research appropriate?* |  |
| Schumann (2012)^52^ | Y | Y | Y | PY | Y | Y | Y | Y | Y | Y | Y | **10.5** |
| Sinnott (2013)^53^ | Y | Y | PY | Y | Y | Y | Y | Y | Y | Y | Y | **10.5** |
| De Vleminck (2013)^58^ | Y | Y | U | PY | Y | Y | Y | Y | Y | Y | Y | **9.5** |
| O’Brien (2016)^62^ | Y | Y | N | PY | Y | Y | Y | Y | Y | Y | Y | **9.5** |
| Barley (2011)^57^ | Y | Y | Y | PY | Y | Y | Y | Y | N | PY | Y | **9** |
| Lucas (2015)^51^ | Y | Y | PY | Y | Y | Y | Y | Y | Y | PY | NA | **9** |
| Zwolsman (2012)^65^ | Y | Y | Y | PY | Y | Y | Y | Y | N | PY | Y | **9** |
| Lawrence (2016)^59^ | Y | Y | PY | PY | U | Y | Y | Y | Y | PY | Y | **8.5** |
| Sirdifield (2013)^54^ | Y | Y | PY | PY | Y | Y | Y | Y | N | PY | Y | **8.5** |
| Vedel (2011)^55^ | Y | Y | U | PY | Y | Y | Y | Y | N | PY | Y | **8** |
| Ju (2018)^50^ | Y | U | Y | Y | Y | Y | N | Y | N | PY | Y | **7.5** |
| McDonagh (2018)^60^ | Y | Y | PY | PY | Y | Y | Y | Y | N | PY | NA | **7.5** |
| Mikat-Stevens (2015)^61^ | Y | Y | U | N | Y | Y | Y | Y | N | PY | Y | **7.5** |
| Carlsen (2007)^49^ | Y | Y | PY | PY | U | U | U | Y | N | PY | Y | **5.5** |
| Schadewaldt (2013)^63^ | Y | Y | N | Y | Y | N | N | N | N | PY | Y | **5.5** |
| Tonkin-Crine (2011)^66^ | Y | Y | PY | PY | Y | U | U | Y | N | PY | NA | **5.5** |
| Vogt (2005)^56^ | Y | Y | U | PY | NA | NA | U | Y | N | Y | Y | **5.5** |
| Yeung (2015)^64^ | N | U | U | PY | U | U | Y | Y | N | PY | NA | **3.5** |
| Ogeil (2020)^33^ | Y | N | U | PY | NA | NA | U | Y | N | PY | NA | **3** |
| Total | **18** | **16** | **7.5** | **11** | **14** | **13** | **13** | **18** | **6** | **12** | **14** |  |

*Y=Yes, N=No, PY=Partial yes, U=Unclear, NA=Not applicable. Yes=1 point, Partial yes=0.5 points, No/Unclear/Not applicable=0 points. Quality: low (≤4 points), moderate (>4 and <8 points), high (≥8 points)*
