# Supplementary material for: Barriers and facilitators to clinical behaviour change by primary care practitioners: a theory-informed systematic review of reviews using the Theoretical Domains Framework and Behaviour Change Wheel
Source: Syst Rev. 2022 Aug 30;11:180. doi: 10.1186/s13643-022-02030-2 (PMC9429279; doi:10.1186/s13643-022-02030-2)
Supplement: Supplementary file 4 — Additional file 4. Additional data. Characteristics of included reviews. [file 13643_2022_2030_MOESM4_ESM.docx]

**Additional File 4**

**Characteristics of included reviews: additional data**

| **First author (year)** | **Objective(s)** | **Sources searched (date limit)** | **Range (years) of included studies** | **Types of studies (number)** |
| --- | --- | --- | --- | --- |
| Sinnott (2013)^53^ | To synthesise the existing published literature on the perceptions of GPs or their equivalent on the clinical management of multimorbidity and determine targets for future research that aims to improve clinical care in multimorbidity. | •EMBASE, MEDLINE, CINAHL, PsycInfo, Academic Search Complete, SocIndex, Social Science  •Full Text and digital theses/online libraries (inception-September 2012) | 2009-2012 | 10 studies:  Qualitative interviews (5),  focus groups (5) |
| O’Brien (2016)^62^ | To ascertain PCPs’ perceptions of the barriers that prevent effective management of child and adolescent mental health problems. | PsycINFO, MEDLINE, Embase, and Web of Science (inception-October 2014) | 1984-2014 | 43 studies:  •Quantitative surveys/questionnaires (30)  •Qualitative interviews/focus groups/open-ended comments (13) |
| Barley (2011)^57^ | To identify potential barriers to and facilitators for good depression management in UK primary care. | Medline, Embase, Psychinfo,  British Nursing Index and Archives (2000-2008) | 2000-2007 | 17 studies:  •Quantitative surveys/questionnaires/ vignettes (10)  •Qualitative interviews (7) |
| *Lucas (2015)^51^ | To review the literature on the views, beliefs, and attitudes of parents, children, and prescribing clinicians that influenced prescribing decisions for acute childhood infection in primary care. | •MEDLINE, EMBASE, CINAHL, PsycINFO, SSCI, SIGLE,  Dissertation Express, NHS economic Evaluation (inception-October 2012).  •Hand searching in Social Science and Medicine, Sociology of Health and Illness, British Journal of  General Practice, Journal of Family Practice, Health Expectations | 1998-2011 | 15 studies:  Qualitative interviews (10), focus groups (4), recorded consultations (1) |
| Lawrence (2016)^59^ | To summarise the available evidence on PCPs’ perspectives of their role within cancer care. | MEDLINE, MEDLINE In-Process, EMBASE, PsycINFO and CINAHL (1993-2015) | 1994-2014 | 35 studies:  •Cross-sectional surveys/ questionnaires (22)  •Qualitative interviews/focus groups/questionnaires (8), methods unclear (4), qualitative part of RCT (1) |
| Sirdifield (2013)^54^ | To systematically review and meta-synthesise qualitative studies exploring clinicians’ experiences and perceptions of benzodiazepine prescribing to build an explanatory model of processes underlying current prescribing practices. | MEDLINE, CINAHL, Social Science Citation Index, Science Citation Index, PsycINFO, Sociological  Abstracts and AMED (January 1990-August 2011) | 1993-2010 | 8 studies:  Qualitative interviews (7),  focus groups (1) |
| Tonkin-Crine (2011)^66^ | To examine GPs’ attitudes and experiences of antibiotic prescribing and interventions aimed at more prudent prescribing for acute respiratory tract infections. | EMBASE, MEDLINE, Web  of Science, PsychInfo,  CINAHL (1950-May 2011) | 1998-2011 | 12 studies:  Qualitative interviews (10),  focus groups (2) |
| Schumann (2012)^52^ | To investigate how family physicians diagnose depression  and what their concepts of depression and the perceived barriers are when diagnosing depression. | Pubmed, PsychINFO and Embase  (inception-April 2010) | 1995-2009 | 15 articles reporting 13 studies:  Qualitative interviews (11), mixed (2) |
| McDonagh (2018)^60^ | To identify barriers and facilitators to chlamydia testing for young people and primary care practitioners in general practice. | MEDLINE, PubMed, Embase, Informit, Web of Science, PsycINFO, Scopus  (January 2000- April 2018) | 2003-2017 | 25 studies:  •Qualitative interviews/focus groups (12), qualitative evaluation of intervention (1), qualitative feasibility study for RCT (1)  •Quantitative questionnaires (5), questionnaires following pilot RCT (2), uncontrolled before-and-after trial (1)  •Mixed methods (3) |
| Ogeil (2020)^33^ | To examine barriers to the assessment and diagnosis of insomnia in family practice from both the clinician and patient perspective. | MEDLINE and Google Scholar | 1980-2015 | 10 studies:  •Quantitative surveys/ questionnaires (7)  •Qualitative focus groups/interviews (3) |
| Yeung (2015)^64^ | To identify the barriers and facilitators to chlamydia testing in the primary care setting. | Medline, PubMed, Informit Health Collection (Meditext), PsycInfo, Scopus and Web of Science (1997-November 2013) | 1998-2012 | 37 studies:  •Quantitative surveys/ questionnaires (17), RCTs (5), audits (2), retrospective analysis (1), evaluations (1), observational descriptive (1), screening programme (1), uncontrolled intervention study (1)  •Qualitative interviews/ focus groups (4)  •Mixed methods (4) |
| De Vleminck (2013)^58^ | To identify the perceived factors hindering or facilitating GPs in engaging in advance care planning (ACP) with their patients about care at the end of life | PubMed, CINAHL, EMBASE, PsycINFO | 1990-2011 | 15 studies:  •Qualitative interviews/focus groups (8)  •Cross-sectional questionnaires/analysis of death certificates (7) |
| *Vogt (2005)^56^ | To estimate the proportion of GPs and family physicians with negative beliefs and attitudes towards discussing smoking cessation with patients. | AMED, CANCERLIT CINAHL, EMBASE, MEDLINE DAILY  UPDATE, MEDLINE, PsycLIT HMIC, PreMedline (inception-January 2005) | 1988-2004 | 20 studies:  Surveys (18), surveys part of intervention studies (2) |
| Zwolsman (2012)^65^ | To determine the barriers encountered by GPs in the practice of evidence-based medicine (EBM) and to come up with solutions to the barriers identified. | MEDLINE (PubMed), Embase, CINAHL, ERIC, Cochrane Library (inception-February 2011) | 1997-2010 | 22 studies:  •Cross-sectional surveys (11), survey part of RCT (1)  •Qualitative interviews/focus groups/Balint style groups (9)  •Mixed methods (1) |
| Carlsen (2007)^49^ | To explore and synthesise qualitative research on GPs’ attitudes to and experiences with clinical practice guidelines. | PubMed, CINAHL, EMBASE, Social Science Citation Index, and  Science Citation Index (inception-Nov 2006) | 1998-2006 | 12 studies:  Qualitative interviews (5), focus groups (7) |
| Vedel (2011)^55^ | To determine the barriers and facilitators to breast and colorectal cancer screening of older adults, from the perspectives of patients and PCPs. | Medline, Web of Science,  Cochrane Database of Systematic Reviews, EMBASE, CINAHL, PsychInfo, SocINDEX, Sociofile/  Sociological Abstract (2000-August 2008) | 2001-2008 | 21 studies:  Cross-sectional surveys (19), surveys part of trial (1), longitudinal observational study (1) |
| Ju (2018)^50^ | To describe the perspectives of GPs on the  prevention of cardiovascular disease (CVD) across different contexts, to support decisions and implementation of evidence-based strategies for the prevention of CVD and improved healthcare outcomes. | •MEDLINE, Embase, PsycINFO and CINAHL (inception to 15 April 2018)  •ProQuest Dissertation and Thesis database, British Library  Electronic Digital Thesis Online Service, Europe E-theses Portal for doctoral dissertation  •Primary care Journals and Google Scholar | 1994-2017 | 34 studies:  Qualitative interviews (22), focus groups (5), questionnaires (4), mixed (3) |
| Mikat-Stevens (2015)^61^ | To systematically review the literature to identify primary care providers’ perceived barriers against provision of genetics services. | PubMed and ERIC (200 Australia (4), 2012) | 2001-2011 | 38 studies:  •Quantitative surveys (22), methods unclear (1), case-control (1)  •Qualitative interviews/focus groups (12), methods unclear (1)  •Mixed methods (1) |
| Schadewaldt (2013)^63^ | To summarise the existing evidence about the views and experiences of nurse practitioners and medical practitioners with collaborative practice in primary healthcare settings. | Cochrane Library, the  Joanna Briggs Institute Library, PubMed, Medline, CINAHL, Informit, ProQuest (January 1990 to September 2012) | 1990-2012 | 30 articles reporting 27 studies:  •Qualitative interviews/focus groups (12), qualitative part of mixed methods studies (2)  •Quantitative surveys (9), survey part of mixed methods study (1), cross-sectional part of mixed methods study (1)  •Mixed methods evaluation of RCT (1), mixed methods quasi-experimental study (1) |

**Clarification obtained from authors on search strategy.*
