# Supplementary material for: Barriers and facilitators to clinical behaviour change by primary care practitioners: a theory-informed systematic review of reviews using the Theoretical Domains Framework and Behaviour Change Wheel
Source: Syst Rev. 2022 Aug 30;11:180. doi: 10.1186/s13643-022-02030-2 (PMC9429279; doi:10.1186/s13643-022-02030-2)
Supplement: Supplementary file 5 — Additional file 5. Evidence mapping. Mapping of emergent themes to the Theoretical Domains Framework (TDF). Evidence table. [file 13643_2022_2030_MOESM5_ESM.docx]

**Additional File 5**

**Mapping of emergent themes to the Theoretical Domains Framework (TDF)**

| **COM-B** | **TDF domain** | **Theme** | **Subtheme** | **Identified** | **Identified as important** |
| --- | --- | --- | --- | --- | --- |
| **CAPABILITY** | **Knowledge** | **Knowledge, awareness and uncertainty** |  | **18** | **13** |
|  |  | Experience |  | 13 | 4 |
|  |  | Perceived complexity |  | 4 | 2 |
|  | **Skills** | Skills and competence |  | 14 | 9 |
|  |  | Difficult discussions |  | 8 | 2 |
|  | **Behavioural regulation** | Medicolegal risk and legislation |  | 10 | 2 |
|  | **Memory, attention and decision processes** | Decision processes |  | 7 | 3 |
|  |  | Memory |  | 3 | 1 |
| **OPPORTUNITY** | **Environmental context and resources** | **Time, workload and general resources** |  | **19** | **13** |
|  |  | **Guidelines, evidence and decision-making tools** |  | **14** | **5** |
|  |  | **Financial resources and insurance coverage** |  | **12** | **6** |
|  |  | **Education and training** |  | **13** | **5** |
|  |  | Availability of and access to specialist services |  | 10 | 4 |
|  |  | Diagnostic and management options |  | 9 | 3 |
|  |  | Electronic systems and communication |  | 8 | 3 |
|  |  | Workforce and support |  | 7 | 2 |
|  |  | Physical environment |  | 8 | 1 |
|  |  | Processes and pathways |  | 6 | 1 |
|  | **Social influences** | **Patient/carer characteristics** | **Perceived ideas, concerns, expectations and motivation** | **14** | **7** |
|  |  |  | Demographic and socioeconomic factors | 9 | 1 |
|  |  |  | Health status and comorbidity | 5 | 1 |
|  |  |  | Culture and religion | 4 | 0 |
|  |  |  | Fear and ability to cope | 4 | 0 |
|  |  |  | Education and awareness | 4 | 1 |
|  |  |  | Diagnostic group | 2 | 1 |
|  |  | **PCP-patient relationship and patient-centred care** | **PCP-patient relationship and patient-centred care** | **15** | **9** |
|  |  |  | Continuity of care | 5 | 1 |
|  |  | **Collaboration and communication with health professionals** | **Collaboration and communication with health professionals** | **10** | **7** |
|  |  |  | Hierarchy and power | 2 | 1 |
|  |  | **Norms, stigma and attitudes** |  | **11** | **5** |
|  |  | Communication with patients and consultation context |  | 10 | 1 |
| **MOTIVATION** | **Social/professional role and identity** | Roles and responsibilities |  | 15 | 7 |
|  |  | Ideology and values |  | 6 | 2 |
|  |  | Individual ways of practising and heuristic approach |  | 5 | 2 |
|  |  | Ethical concerns and confidentiality |  | 5 | 1 |
|  |  | PCP characteristics |  | 3 | 0 |
|  |  | Retaining patients |  | 3 | 0 |
|  | **Emotion** | Patient/carer emotions |  | 10 | 2 |
|  |  | PCP emotions |  | 12 | 0 |
|  | **Beliefs about consequences** | Consequences of prescribing |  | 6 | 2 |
|  |  | Futility |  | 5 | 2 |
|  |  | Medicalisation |  | 4 | 2 |
|  | **Reinforcement** | Incentives |  | 12 | 3 |
|  |  | Feedback |  | 3 | 0 |
|  | **Beliefs about capabilities** | Confidence in own ability |  | 9 | 5 |
|  |  | Confidence in testing and treatments |  | 9 | 2 |
|  | **Goals** | Goals |  | 3 | 0 |
|  | **Optimism** | Optimism |  | 1 | 1 |
|  |  | Pessimism |  | 4 | 0 |
|  | **Intentions** | Intentions |  | 2 | 0 |

**Most important TDF domains**

**Most important themes/subthemes within the most important TDF domains**

Themes not considered important

**Evidence table**

| **TDF Domain** | **Theme** | **Subtheme** | **First author (year)** | **Evidence** |
| --- | --- | --- | --- | --- |
| **Knowledge** | **Knowledge, awareness and uncertainty** |  | Barley (2011) | Barriers  *In older people, uncertainty among GPs was found as to the effectiveness of antidepressants, drug interactions and side-effects.*  *That GPs and PNs felt that they lacked knowledge and wanted more training was a consistent finding.*  *That GPs and PNs are aware of the relationship between social and mood problems is clear from this review, but they are unsure of its exact nature and of their role in managing it.* |
|  |  |  | De Vleminck (2013) | Barriers  *Knowledge. Medium evidence was found for the reported lack of GP knowledge about ACP as a barrier to involving patients in ACP.* |
|  |  |  | Ju (2018) | Barriers  *Some GPs trusted research evidence and expert opinion to feel secure about their decisions. Guidelines, risk assessment tools and ‘editorials in the (British Medical Journal) BMJ’ were seen to minimise room for human error and were more reliable than their own judgement—‘I’m comfortable to be guided by the experts rather than try and invent too much on what might be dodgy assumptions on my part’.*  *For patients with comorbidities, some GPs considered specialists (e.g. psychiatrists, cardiologists) to have more authority in educating their patients, as they had better knowledge of the patient’s condition and medication.*  Facilitators  *Greater awareness of and adherence to evidence-based guidelines on medications for asymptomatic patients and risk factors may improve consistency of evaluating and managing CVD risk in patients.* |
|  |  |  | Lawrence (2016) | Barriers  ***PCPs recognised that their lack of knowledge could affect doctor–patient communication.***  ***Ambiguity about the nature, frequency and extent of surveillance testing was expressed by the majority of PCPs.***  ***However, lower levels of training or knowledge were reported for areas such as surveillance testing, active treatment or surgery and advising on complementary therapies, leading to a reluctance to fulfil such roles.*** |
|  |  |  | Lucas (2015) | Factors  ***More often, clinicians reported prescribing when uncertainty existed either because of the lack of diagnosis, or uncertainty regarding the social, health, or legal consequences of not prescribing and therefore took a “just in case” approach.*** |
|  |  |  | McDonagh (2018) | Barriers  ***Lack of knowledge about the epidemiology and presentation of chlamydia, benefits of testing, at-risk populations such as young people, how to take specimens, and treatment options were described. Practitioners who were unaware of the public health importance of testing and screening programmes would be less likely to find chlamydia testing a priority.***  Facilitators  *Increasing knowledge, awareness, and education “The support raised my awareness and it gave me different ways of approaching young people…I think it does one good to have one’s awareness raised”.*  *GPs with postgraduate education in STIs were more willing to offer testing to men as well as indicating greater knowledge of the need to offer to both men and women.* |
|  |  |  | Mikat-Stevens (2015) | Barriers  ***PCPs approve of genetic testing for hereditary breast cancer and want to play a central role in management of families, but lack knowledge to effectively do so.*** |
|  |  |  | O’Brien (2016) | Barriers  ***Uncertainty regarding ‘the lack of clarity’ about how other services are structured and governed led to lack of confidence.***  ***Primary care practitioners play a crucial ‘gatekeeper’ role to specialist services for children and young people with mental health problems, yet they face numerous barriers, in particular a lack of time, knowledge, reimbursement, mental health providers, and resources.*** |
|  |  |  | Ogeil (2020) | Barriers  ***These barriers include a lack of physician awareness of the negative physical and mental health impacts associated with insomnia, as well as limited training opportunities to develop expertise with sleep problems.***  ***While education of physicians has been demonstrated to improve sleep knowledge, the present article has identified that many in family practice rate their knowledge as either ‘fair’ or ‘poor’ and that there are gaps in their knowledge when assessing insomnia.*** |
|  |  |  | Schadewaldt (2013) | Barriers  ***The most common barrier to collaboration was the lack of awareness by MPs of the scope of practice of NPs, their level of education and what is inherent to their role.*** |
|  |  |  | Schumann (2012) | Barriers  *Patients seen in primary care settings often reported symptoms that were unclear and could indicate any of several diagnoses, including depression. ‘Every patient comes with a different combination and it’s sort of like solving a puzzle. You have to try and ﬁnd out what are the basic causes or underlying problems’.*  Facilitators  *Knowledge of the whole patient is considered an important factor to help assess the syndromal cues presented by the patients.*  Factors  ***The synthesis revealed that FPs use approaches to diagnose depression that are usually based on their knowledge of the patient’s long-term history, an established patient–doctor relationship and a rule-out algorithm of other diagnoses.*** |
|  |  |  | Sinnott (2013) (2013) | Barriers  ***Challenges in shared decision-making: The patient’s role in decision-making in multimorbidity is limited by difﬁculties in communicating risk beneﬁt and outcomes in a ﬁeld where there is much more uncertainty on these issues.*** |
|  |  |  | Sirdifield (2013) | Barriers  *GPs often made prescribing decisions in the context of uncertainty and in the short timescales and pressures of the consultation.* |
|  |  |  | Tonkin-Crine (2011) | Barriers  ***GPs may feel uncertain about making an ARTI diagnosis, about having access to patients for review or about following guideline advice when they query its relevance to an individual. Many may feel uncertain about potential illness consequences when not prescribing and this may lead to inappropriate prescribing as a way to ensure that patients are protected.***  Facilitators  *Interventions may decrease uncertainty about an ARTI diagnosis and/or management as they offer additional information to inform decisions and/or aspects to increase GP conﬁdence.* |
|  |  |  | Vedel (2011) | Barriers  ***Other reported barriers are linked to the lack of knowledge (7 studies): lack of awareness about cancer screening, lack of skills, lack of training, lack of clear guidelines.***  Facilitators  ***The facilitators to screening most commonly identified by PCPs were linked to physicians' knowledge (10 studies): knowledge/awareness of screening and cancer, skills, training, existing useful guidelines.*** |
|  |  |  | Vogt (2005) | Barriers  ***A substantial proportion of physicians indicated that they felt they did not have the necessary skills or knowledge to discuss smoking with their patients, suggesting the need for more effective training.*** |
|  |  |  | Yeung (2015) (2015) | Barriers  ***Another signiﬁcant barrier was the lack of knowledge, skill, training and awareness around chlamydia testing. Many GPs were not aware of the key risk factors, or the use of urine testing protocols, or the epidemiology of the infection.***  Facilitators  *Increased education/awareness/training Increasing education, awareness and training was acknowledged as a facilitator for testing.* |
|  |  |  | Zwolsman (2012) | Barriers  *Lack of knowledge and skills also influences GPs’ use of EBM.* |
|  | **Experience** |  | Barley (2011) | Barriers  *It is also because of findings that a negative past experience of mental health training was associated with PNs’ current negative attitudes towards engaging with patients’ mental health needs.* |
|  |  |  | Carlsen (2007) | Factors  ***All of the studies report that GPs described a tension between their own experiences and the guideline recommendations, and that GPs saw consultations with real patients as more complicated than their portrayal in the guidelines.*** |
|  |  |  | De Vleminck (2013) | Facilitators  *Experience. Medium evidence was found for the length of their experience as a GP and having a living will themselves as perceived facilitators.*  *Lower evidence supported positive experiences with end-of-life conversations in the past as a facilitator.* |
|  |  |  | Ju (2018) | Facilitators  *Our findings indicate that GPs may prefer to make their own judgement of individual risk factors acquired through experience rather than using absolute risk assessment tools.* |
|  |  |  | Lawrence (2016) | Barriers  *High confidence was reported for non-cancer specific tasks such as pain management and psychosocial support, while confidence was lower for active treatment, surveillance testing and management of long-term effects, suggesting PCPs are willing but feel unprepared to manage certain aspects of cancer specific care.* |
|  |  |  | Schadewaldt (2013) | Facilitators  ***Positive experiences of working collaboratively may be the strongest force to promote and advance collaboration between NPs and MPs.***  *Nurse practitioners, more than MPs, seemed confident in autonomous NP practice, but MPs who worked with NPs showed more trust in the NPs’ capabilities and support for autonomous NP work than MPs who lacked this experience. The reasons for this may be that the MPs’ work experience with the NP increased their confidence in the benefits of collaboration or that MPs who have a positive attitude about collaboration with a NP are more likely to work with one. Consequently NPs rely on the support and willingness of MPs to work with them.* |
|  |  |  | Schumann (2012) | Factors  *Other FPs described that they consider a diagnosis of depression only after all other possibilities have been explored. The type of approach that the FPs used was mostly based on personal and professional experience and the familiarity with the patient.*  Facilitators  *General experience was seen as necessary to acquire experiential knowledge. This special knowledge had been gained over the course of practicing and enabled the FPs—in addition to textbook knowledge—to familiarize themselves with different illness patterns or to recognize verbal and nonverbal cues.* |
|  |  |  | Sinnott (2013) | Factors  *In response to difﬁculties in shared decision-making, GPs employed a range of techniques including prioritisation of the doctor’s or the patient’s agenda, drawing on one’s own personal experience, avoidance of decision-making, or using additional investigations to support a decision.* |
|  |  |  | Sirdifield (2013) | Factors  *Attitudes were also affected by GPs’ personal use of benzodiazepines: “Your own attitude towards and experiences of the product definitely has an effect on prescribing. We, ourselves, take a lot of benzodiazepines”.* |
|  |  |  | Tonkin-Crine (2011) | Barriers  ***GPs’ concern about ARTI management may increase if they have had a previous negative experience with prescribing or withholding antibiotics. Alternatively, a lack of experience may mean that GPs are less able to manage complex ARTI consultations, where there are patient expectations for antibiotics or where management is unclear, as effectively as more-experienced colleagues.***  Factors  *One practitioner explained that his high prescribing was grounded in an experience when he withheld antibiotics and the patient subsequently developed streptococcal septicaemia.* |
|  |  |  | Vogt (2005) | Barriers  *Sixteen per cent of GPs and FPs experienced discussing smoking cessation as unpleasant. This could be because they had experienced negative reactions or lack of enthusiasm from patients.* |
|  |  |  | Yeung (2015) (2015) | Facilitators  *It should be noted that in papers that looked at the association between demographic characteristics of the GP and chlamydia testing, some found that younger or female GPs were more likely to test while others found that GPs with more experience were more likely to test.* |
|  |  |  | Zwolsman (2012) | Factors  ***Personal experiences in life or in clinical practice influence the use of evidence; for instance, deaths in the personal or practice environment due to strokes related to arrhythmias led to a more controlled anticoagulation treatment.*** |
|  | **Perceived complexity** |  | Barley (2011) | Barriers  ***Management of depression is perceived as particularly complex when patients present with social problems. That GPs and PNs are aware of the relationship between social and mood problems is clear from this review, but they are unsure of its exact nature and of their role in managing it.*** |
|  |  |  | Tonkin-Crine (2011) | Barriers  *GPs view ARTI management decisions as complex.*  *GPs’ perceptions of how easy or difﬁcult it is to explain prescribing decisions to patients.* |
|  |  |  | Sinnott (2013) | Barriers  *Delivering patient-centred care was seen as an aid for some but a challenge for others. For instance, some GPs felt that taking a broader view of the patient, incorporating non-medical or psychosocial issues, increased the level of complexity in their management.* |
|  |  |  | Sirdifield (2013) | Barriers  ***Making decisions on whether or not to prescribe was often uncomfortable, demanding and complex within the time and pressure constraints of daily practice.*** |
| **Skills** | **Skills and competence** |  | Barley (2011) | Barriers  *Clinicians struggle to distinguish between ‘normal’ distress and depression requiring treatment.* |
|  |  |  | Carlsen (2007) | Barriers  *Lack of time and technical skills.*  *Other practical constraints, including convenience, lack of skills with new procedures, and lack of resources, were also referred to.* |
|  |  |  | De Vleminck (2013) | Barriers  ***There is stronger evidence that GPs perceive their own lack of skill in dealing with patients ’ vague requests, and their difﬁculties in deﬁning the right moment for initiating discussion, as barriers to engaging in ACP. Medium evidence was found that they perceive their lack of skill in dealing with a patient’s changing preferences and with the emotional impact or discomfort of having ACP discussions as barriers. Lower evidence supported the perceived lack of skill in advising patients on expressing their wishes, and the prognostic uncertainty for chronically ill patients, as barriers.***  Facilitators  *The perceived facilitators for which stronger evidence was found were accumulated skills, the ability to foresee health problems in the future, skills in addressing patient initiation of ACP, cancer patients, patients capable of decision-making, a longstanding patient – GP relationship, and a home setting.* |
|  |  |  | Lawrence (2016) | Barriers  ***PCPs have indicated an interest in increasing their role in cancer patient care; however, PCPs’ limited confidence and ability to perform the various roles pose a challenge.***  ***Between 60 and 70 % of PCPs expressed confidence in their skills for performing follow-up care; however, about half felt unprepared to manage long-term side-effects or conduct surveillance for recurrence.***  ***Improving PCP confidence to deliver cancer care is critical before a model of shared care is recommended.*** |
|  |  |  | Mikat-Stevens (2015) | Barriers  ***Among the several skill-related barriers identified, the most commonly cited (n = 16 citations in 16/38 studies) was a lack of confidence their ability to counsel patients about genetic risk and to manage them. Specifically, providers expressed concerns about feeling unqualified to provide genetic counselling to patients and making the correct management decisions.***  ***Although four major barrier themes (knowledge/skills, systems, ELSI, and evidence) were identified, barriers related to insufficient knowledge and skills were the most frequently cited across the literature.*** |
|  |  |  | McDonagh (2018) | Barriers  ***Lack of training and skills. PCPs reported a lack of appropriate training and skills needed to discuss sexual health, take sexual history, offer a test, respond to a positive test and manage treatment, and conduct partner notification. This led to reduced confidence to offer testing (reflective motivation) and discuss sexual health.*** |
|  |  |  | O’Brien (2016) | Barriers  *Primary care practitioners also clearly identified a lack of confidence in recognising childhood mental health problems and a lack of training in this area, which, given the prevalence of such issues, is resulting in a serious skill gap.* |
|  |  |  | Ogeil (2020) | Barriers  ***These barriers include a lack of physician awareness of the negative physical and mental health impacts associated with insomnia, as well as limited training opportunities to develop expertise with sleep problems.***  ***Whilst being able to detect that a person was having problems with their sleep, physicians reported difficulty in defining the underlying cause and/ or identifying the correct treatment.*** |
|  |  |  | Schadewaldt (2013) | Barriers  *Some of these concerns were also identified as barriers to collaborative practice such as concern about: NP education and competence.*  Facilitators  ***While having complementary skills and similar goals was seen as an asset to collaboration, ideological differences in the practice style could cause difficulties in establishing a collaborative relationship.***  ***Complementary skills and practice ideology (of NPs).*** |
|  |  |  | Schumann (2012) | Facilitators  *Professional qualities and skills were considered crucial in the diagnostic process.* |
|  |  |  | Vedel (2011) | Barriers  *Other reported barriers are linked to the lack of knowledge (7 studies): lack of awareness about cancer screening, lack of skills, lack of training, lack of clear guidelines.*  Facilitators  *The facilitators to screening most commonly identified by PCPs were linked to physicians' knowledge (10 studies): knowledge/awareness of screening and cancer, skills, training, existing useful guidelines.* |
|  |  |  | Vogt (2005) | Barriers  ***A substantial proportion of physicians indicated that they felt they did not have the necessary skills or knowledge to discuss smoking with their patients, suggesting the need for more effective training.*** |
|  |  |  | Yeung (2015) (2015) | Barriers  ***Another signiﬁcant barrier was the lack of knowledge, skill, training and awareness around chlamydia testing.*** |
|  |  |  | Zwolsman (2012) | Barriers  ***Lack of knowledge and skills also influences GPs’ use of EBM. 55.6%feel that training in EBM is required, and that there is a lack of such training. A lack of skills in searching and in accessing resources is a barrier: not knowing where to search, difficulty in finding evidence (or finding it quickly), are among the barriers mentioned by GPs.*** |
|  | **Difficult discussions** |  | De Vleminck (2013) | Barriers  *There is stronger evidence that GPs perceive their own lack of skill in dealing with patients ’ vague requests, and their difﬁculties in deﬁning the right moment for initiating discussion, as barriers to engaging in ACP.*  *Medium evidence was found that they perceive their lack of skill in dealing with a patient ’ s changing preferences and with the emotional impact or discomfort of having ACP discussions as barriers.* |
|  |  |  | Lawrence (2016) | Barriers  *Lack of skill in discussing emotions identified by 13 % of PCPs.*  *PCPs saw talks as difficult if relaying the diagnosis.* |
|  |  |  | Lucas (2015) | Barriers  *They also wanted to educate parents to understand that antibiotics were not necessary [21,29], but found this difﬁcult to achieve.* |
|  |  |  | McDonagh (2018) | Barriers  ***Some, especially older male PCPs, found it difficult to discuss sexual health with patients due to personal discomfort. This was particularly a concern in consultations with male patients and in consultations unrelated to sexual health.*** |
|  |  |  | Mikat-Stevens (2015) | Barriers  *Communicate consequences of having a screening test and results.*  *Difficult to explain false negatives/positives and limitations of screening tests to patients.*  *PCPs were unsure how to address consanguinity with their patients.* |
|  |  |  | Sinnott (2013) | Barriers  *Discussing the risks and outcomes associated with treatment options in a way facilitated that patient involvement was particularly challenging, as was discussing the balance between quantity and quality of life.*  Facilitators  *Enhanced-communication skills were seen as necessary in multimorbidity to facilitate clear and concise discussion with patients on the interplay between their chronic diseases and to help with de-prescribing medications, which if carried out badly could be interpreted as withdrawing care.* |
|  |  |  | Vogt (2005) | Barriers  ***Just over a quarter (22%) of physicians reported lacking confidence in their ability to discuss smoking with their patients, 18% felt such discussions were unpleasant.*** |
|  |  |  | Yeung (2015) (2015) | Barriers  *A lack of sexual health training in medical school was reported and manifested as discomfort in communicating sexual health-related issues with patients.* |
| **Social/professional role and identity** | **Roles and responsibilities** |  | Barley (2011) | Barriers  ***That GPs and PNs are aware of the relationship between social and mood problems is clear from this review, but they are unsure of its exact nature and of their role in managing it.***  ***Studies of late-life depression indicated that GPs and PNs may have conflicting views of their roles. GPs perceived PNs as having a limited role in the identification and management of late-life depression. None of the participants in one study could recall a nurse referring a case to them; the GPs did not refer to nurses as they felt PNs have enough to do. In contrast, PNs saw some GPs as demotivated and unwilling to engage with depressed patients. In another study, PNs felt they were in a better position to deal with depression than GPs as they had more time to explore psychosocial difficulties and operated in a less ‘medical’ context.*** |
|  |  |  | Carlsen (2007) | Barriers  ***In the proscriptive studies the focus is on the dilemmas of combining the role of gatekeeper and the role of patient advocate; such studies refer to GPs’ concerns that rationing may harm the doctor–patient relationship or even lead to litigation.***  Factors  ***An alternative view was provided by three papers that reported that GPs did not see guidelines as providing fixed rules for practice; instead, they felt it was the responsibility of the GP to adapt guidance to suit circumstances.*** |
|  |  |  | De Vleminck (2013) | Barriers  ***Stronger evidence was found for lack of skills to deal with vague requests, difﬁculties with deﬁning the right moment, the attitude that patients should initiate ACP, and fear of depriving them of hope as perceived barriers.***  *There is medium evidence that a conviction that it is their job to cure people whereas other healthcare professionals should initiate ACP prevents GPs engaging in ACP.*  Facilitators  *The attitude that GPs should initiate ACP was perceived as a facilitator for which stronger evidence was found.* |
|  |  |  | Ju (2018) | Barriers  ***Some GPs believed their core role, as a physician, was to ‘offer the tablets’ and prescribe medicines, whereas counselling patients to make lifestyle changes was a secondary focus.***  ***Some GPs defined their role as an ‘influencer’ in their patients’ self-motivation and management. They could only provide information but believed it was ultimately the patients’ duty to make lifestyle changes or take their medication. Enforcing medications and behavioural change on patients was deemed unethical and not within their professional purview, and seen as ‘presumptuous to make such strong demands’.*** |
|  |  |  | Lawrence (2016) | Barriers  ***They demarcated their role to focus more on the patient’s quality-of-life, dependent on their personal relationships, familiarity with their social environments and knowledge of the patients past medical history. They saw themselves as a resource for patients, more concerned with symptom management and the psychological dimensions of cancer care compared to a disease or treatment focus.***  ***Similarly, some PCPs expressed frustration at their lack of involvement during treatment, though others agreed that treatment was best administered in a hospital setting and need for their input was minimal.*** |
|  |  |  | Mikat-Stevens (2015) | Barriers  *PCPs also reported being unclear about their role in providing genetics services (n = 15 cited in 15/38 studies). In some instances, PCPs felt that it was not their role to deliver genetics services or they simply were unclear about their role. Additional studies described PCPs’ perceptions that “genetics is for specialists” and that the management of genetic conditions requires complex high-level knowledge available only from a specialist* |
|  |  |  | McDonagh (2018) | Barriers  *Some believed responsibility for partner notification should lie with sexual health clinics, and any increase in testing should be accompanied by an increase in staffing.* |
|  |  |  | O’Brien (2016) | Barriers  *Uncertainty as to what is expected of practitioners.* |
|  |  |  | Ogeil (2020) | Factors  *From the practitioner’s viewpoint, they are often the first point of call for any health or medical complaint and are in a position to provide the relevant support and resources.* |
|  |  |  | Schadewaldt (2013) | Barriers  ***The most common barrier to collaboration was the lack of awareness by MPs of the scope of practice of NPs, their level of education and what is inherent to their role.***  ***Medical practitioners rarely saw NPs as autonomous health professionals.***  ***Some of these concerns were also identified as barriers to collaborative practice such as concern about: NPs’ limited scope of practice for patients with multiple comorbidities, ultimate liability for NP care.***  Facilitators  ***Clarity of NP role & scope of practice.***  ***High level of NP autonomy.***  ***Official recognition of the NP role, including the legal protection of the professional title ‘nurse practitioner’.*** |
|  |  |  | Sinnott (2013) | Barriers  *In some studies, GPs had a broad sense of responsibility towards overseeing and screening patients’ medications; others were unsure about their role in screening prescriptions and felt that a clear line of responsibility was required.* |
|  |  |  | Sirdifield (2013) | Barriers  *Some GPs felt a sense of responsibility for past (which they now considered) poor prescribing practices, whilst others thought that this negative perception was overstated. There was tension between wanting to help patients, and feeling responsible for minimising BZD use.* |
|  |  |  | Tonkin-Crine (2011) | Factors  ***Discussion and comparison with peers may be very influential because of a GP’s motivation to fit with professional norms.*** |
|  |  |  | Vedel (2011) | Facilitators  *Other facilitators were linked to physicians' attitudes (8 studies): agreement with current screening policies or guidelines, perceived responsibility for cancer screening.* |
|  |  |  | Vogt (2005) | Factors  *Only a small minority of GPs and FPs thought that dis- cussing smoking was intruding upon patients' privacy, that it was not their professional duty, and that it was not appropriate. These findings suggest that, although GPs and FPs have concerns about time and efficacy, they generally accept that intervening with their smoking patients is an appropriate part of their role.* |
|  | **Ideology and values** |  | Barley (2011) | Factors  Professionals’ understanding of depression Two contrasting understandings were identified: depression as a normal response to life events and a biomedical model of depression. |
|  |  |  | Mikat-Stevens (2015) | Barriers  *Personal/professional value conflicts (Negative impact of management option is in conflict with their value system)* |
|  |  |  | Schadewaldt (2013) | Barriers  ***While having complementary skills and similar goals was seen as an asset to collaboration, ideological differences in the practice style could cause difficulties in establishing a collaborative relationship.***  Facilitators  ***Complementary skills and practice ideology (of NPs).*** |
|  |  |  | Schumann (2012) | Barriers  **Even though FPs were aware of the relevance of social and contextual factors inﬂuencing their patients’ lives, an unresolved inconsistency was present in their description of depression as a medical disorder and the understanding of depression as related to the conditions of their patients’ lives.** |
|  |  |  | Sinnott (2013) | Barriers  It was suggested that specialists did not ‘consider the wider harms and beneﬁts of organ-speciﬁc intervention’, thereby adding to the problems of multimorbidity, in contrast to GPs who had a ‘holistic’ view of the patient; ‘The cardiologists, you know, don’t mind if they bleed to death’. |
|  |  |  | Tonkin-Crine (2011) | Factors  To help patients, some GPs may believe that they must offer something ‘tangible’ (e.g. an antibiotic), whereas others feel that reassurance may prove equally appropriate to counteract patients’ concerns. |
|  | **Individual ways of practising and heuristic approach** |  | Barley (2011) | Barriers  *Clinicians struggle to distinguish between ‘normal’ distress and depression requiring treatment. Some reported using subjective processes: “I have my own kind of mental ways in finding out if people are depressed”.* |
|  |  |  | Ju (2018) | Factors  *Some GPs considered the patient’s family history and background when determining prevention strategies. They advocated the use of ‘human judgement,’ which incorporated ‘emotional, political and logistical’ considerations rather than accepting risk scores unconditionally. Others were unwilling to use risk scores to estimate pretreatment risk due to ambiguity of current guidelines regarding unique patient circumstances.* |
|  |  |  | Schumann (2012) | Facilitators  ***Professional qualities and skills were considered crucial in the diagnostic process. Most of the FPs in the included studies stated that they had adopted their own routine and style of questioning. In addition to experience and self-awareness, intuition and ‘gut feeling’ were considered helpful: ‘... the process of diagnosis is easier if you have your antenna up...gut feeling is better than any tool...’*** |
|  |  |  | Sirdifield (2013) | Factors  ***Overall, GPs were ambivalent towards prescribing benzodiazepines because of the issues described above, ranging from those who rarely prescribed, to those who did not see a problem with prescribing benzodiazepines. For most GPs, located in the middle of this continuum, these were complex decisions leading to conflicting pressures about whether or not to prescribe: “These descriptions were discussed in relation to two imperatives in tension with one another: the moral obligation to ensure a programme of humane withdrawal; and the strict need to restrict access to a wider population. This tension is managed within the daily working constraints of GPs”. These pressures led GPs to adopt a variety of management strategies from minimising benzodiazepine use to using tacit or explicit rules (heuristics) to justify prescribing. Complexity and conflict resulted in inconsistency between GPs in the strategies employed and how these were applied.*** |
|  |  |  | Zwolsman (2012) | Factors  *GPs rely on so-called mindlines, which are ‘collectively reinforced, internalised, tacit guidelines’ that can be modified when integrating external information.* |
|  | **Ethical concerns and confidentiality** |  | Ju (2018) | Barriers  *Enforcing medications and behavioural change on patients was deemed unethical and not within their professional purview, and seen as ‘presumptuous to make such strong demands’.*  Factors  *Some articulated a professional and ethical duty, to prescribe medications for the prevention of CVD and subsequently minimise the risk of future CVD events that could be preventable, and to avoid taking any responsibility for risking the patients’ lives.* |
|  |  |  | McDonagh (2018) | Barriers  *PCPs expressed concern about privacy and confidentiality, particularly in rural areas where they will likely know their patients socially. They were also reluctant to raise testing if a parent was present in the consultation or if the patient’s family was known to staff; which is supported by research with patients. Patient cultural and religious factors could also act as a barrier to testing.* |
|  |  |  | Mikat-Stevens (2015) | Barriers  ***The concern over lack of confidentiality of genetic information, specifically the risk of harm resulting from a breach of privacy and disclosure of information, was cited as a barrier on 10 occasions. Examples of disclosure concerns included revelations of nonpaternity, as well as the implications of uncovering genetic risk for family members of the primary patient.***  *Several issues related to prenatal ethics arose, including the perception that prenatal genetic testing dampens the natural excitement of pregnancy or could medicalize pregnancy and a fear that prenatal and preconception genetic testing would motivate individuals to want to create a “perfect child,” which might create additional disparities in society regarding individuals with genetic conditions or disabilities.* |
|  |  |  | O’Brien (2016) | Barriers  *Confidentiality limitations are a barrier.* |
|  |  |  | Yeung (2015) (2015) | Barriers  *Finally, legal concerns about conﬁdentiality and privacy also hindered a GP’s ability to carry out testing, particularly around partner notiﬁcation.* |
|  | **GP characteristics** |  | De Vleminck (2013) | Facilitators  *Socio-demographic characteristics of GPs. There was medium evidence that the GP being younger was signiﬁcantly and positively associated with the proportion of patients with whom they discussed end of-life decisions.* |
|  |  |  | McDonagh (2018) | Barriers  *Older male colleagues were not comfortable with sexual health work.*  *Judgemental attitudes of healthcare professionals.*  Facilitators  *Characteristic of doctor or nurse: non-judgemental.* |
|  |  |  | Yeung (2015) (2015) | Facilitators  *It should be noted that in papers that looked at the association between demographic characteristics of the GP and chlamydia testing, some found that younger or female GPs were more likely to test while others found that GPs with more experience were more likely to test. This association favours young, female GPs, but indicates that other GPs can be targeted to increase their chlamydia testing.* |
|  | **Retaining patients** |  | Carlsen (2007) | Factors  *Proscriptive guidelines may entail rationing and denial of patients' requests, thereby jeopardising the doctor–patient relationship. This dilemma has been noted and debated; studies reporting this dilemma note that such rationing is both unpleasant and in conflict with the ideals of a patient-centred medicine and the economic incentives of competition for patients.* |
|  |  |  | Sirdifield (2013) | Factors  *Moreover, even when a GP does not believe that benzodiazepines will be an effective solution, they may still decide to prescribe due to a belief that there is a lack of valid alternatives for that patient, or a fear that any attempt to change a patient’s (perceived) preference of drugs over alternatives would be time-consuming or lead to the patient seeking another doctor.* |
|  |  |  | Tonkin-Crine (2011) | Factors  *GPs may also feel that delivering patient satisfaction is crucial when they perceive a risk of losing patients to other doctors.* |
| **Beliefs about capabilities** | **Confidence in own ability** |  | Barley (2011) | Barriers  *A lack of confidence among some clinicians in their ability to manage this condition.*  ***Among some clinicians, ambivalent attitudes to working with depressed people, a lack of confidence, the use of a limited number of management options and a belief that a diagnosis of depression is stigmatising complicate the management of depression.*** |
|  |  |  | Lawrence (2016) | Barriers  ***PCPs have indicated an interest in increasing their role in cancer patient care; however, PCPs’ limited confidence and ability to perform the various roles pose a challenge.***  ***It appears that PCP confidence levels fluctuate along the cancer care continuum and according to the type of care needed. High confidence was reported for non-cancer specific tasks such as pain management and psychosocial support, while confidence was lower for active treatment, surveillance testing and management of long-term effects, suggesting PCPs are willing but feel unprepared to manage certain aspects of cancer specific care.*** |
|  |  |  | McDonagh (2018) | Barriers  ***PCPs reported a lack of appropriate training and skills needed to discuss sexual health, take sexual history, offer a test, respond to a positive test and manage treatment, and conduct partner notification. This led to reduced confidence to offer testing (reflective motivation) and discuss sexual health.*** |
|  |  |  | Mikat-Stevens (2015) | Barriers  *Among the several skill-related barriers identified, the most commonly cited (n = 16 citations in 16/38 studies) was a lack of confidence their ability to counsel patients about genetic risk and to manage them. Specifically, providers expressed concerns about feeling unqualified to provide genetic counselling to patients and making the correct management decisions.* |
|  |  |  | O’Brien (2016) | Barriers  *Primary care practitioners also clearly identified a lack of confidence in recognising childhood mental health problems and a lack of training in this area, which, given the prevalence of such issues, is resulting in a serious skill gap.* |
|  |  |  | Ogeil (2020) | Barriers  *Whilst being able to detect that a person was having problems with their sleep, physicians reported difficulty in defining the underlying cause and/or identifying the correct treatment.* |
|  |  |  | Schadewaldt (2013) | Facilitators  ***To make collaboration work, NPs and MPs have to be confident in the competence of the collaborating partner.*** |
|  |  |  | Schumann (2012) | Facilitators  *Using the term ‘skill confidence’, the FPs described that with time they improved their skills in examining patients, becoming more cognizant of disease presentations, and were comfortable in their professional task.19 Being ‘self-aware’ and ‘confident’ of their skills was seen as important to expedite the process of finding an answer and allowed the FPs to present the diagnosis of depression more confidently.* |
|  |  |  | Vogt (2005) | Barriers  ***No confidence in ability. Statements relating to the degree to which GPs and FPs feel able to discuss smoking with their patients.*** |
|  | **Confidence in testing and treatments** |  | Barley (2011) | Barriers  *GPs may be less likely to refer older patients for psychological therapy, either because they ‘forget’ about it or assume it will not work in this population.* |
|  |  |  | Lawrence (2016) | Barriers  *It appears that PCP confidence levels fluctuate along the cancer care continuum and according to the type of care needed. High confidence was reported for non-cancer specific tasks such as pain management and psychosocial support, while confidence was lower for active treatment, surveillance testing and management of long-term effects, suggesting PCPs are willing but feel unprepared to manage certain aspects of cancer specific care.* |
|  |  |  | McDonagh (2018) | Barriers  *Attitudes to Department of Health screening programme proposals: did not believe the Department of Health had ‘any idea of what really goes on in general practice’ or the ‘extreme pressures GPs were under.* |
|  |  |  | Mikat-Stevens (2015) | Barriers  *Furthermore, PCPs reported that limitations of current genetic screening tests deterred them from utilizing them in practice (n = 10 cited in 7/38 studies). Specific limitations of genetic tests were inaccurate or unambiguous results, a high rate of false positives (in cancer risk testing), and concern over the validity of genetic testing.* |
|  |  |  | Schumann (2012) | Facilitators  *Instead, the FPs mentioned that ‘once a level of suspicion has been established with familiar patients, they could raise the possibility of a mental health diagnosis by asking the few screening questions they have found to be useful rather than going through the ICD10/DSM list of symptoms’. Others found it helpful to use simple checklists/handouts or questionnaires to facilitate the diagnosing of depression. FPs admitted that they used them occasionally rather than routinely, primarily to help patients accept the diagnosis when the FPs anticipated or encountered resistance to the diagnosis.* |
|  |  |  | Sirdifield (2013) | Barriers  *GPs perceived a lack of alternative treatments, depending on their knowledge of alternatives and their views about validity or effectiveness of non-pharmacological options for particular patients.*  *Negative attitudes towards benzodiazepines were based on their perceived risks.*  Facilitators  *The ‘fast acting’ and effective nature of benzodiazepines for some GPs made them preferable to other forms of treatment.* |
|  |  |  | Vedel (2011) | Barriers  ***From the PCPs' point of view, the most often identified physician-related barriers were linked to perception of screening tests (9 studies): absence of belief in the usefulness of cancer screening for older adults and complexity or potential risk of the test.*** |
|  |  |  | Vogt (2005) | Barriers  ***Not effective. Statements referring to physicians' expectations about whether discussing smoking would result in smoking cessation.*** |
|  |  |  | Zwolsman (2012) | Barriers  *Attitude towards EBM: Limited treatment options and clinical freedom, EBM not helpful, EBM will not benefit practice.* |
| **Optimism** | **Optimism** |  | Schadewaldt (2013) | Facilitators  ***Positive attitude towards collaboration.***  ***Nurse practitioners, more than MPs, seemed confident in autonomous NP practice, but MPs who worked with NPs showed more trust in the NPs’ capabilities and support for autonomous NP work than MPs who lacked this experience. The environmental reasons for this may be that the MPs’ work experience with the NP increased their confidence in the benefits of collaboration or that MPs who have a positive attitude about collaboration with a NP are more likely to work with one. Consequently NPs rely on the support and willingness of MPs to work with them.*** |
|  | **Pessimism** |  | Barley (2011) | Barriers  *Negative attitudes included unfavourable views of depressed people themselves e.g. ‘burdens’, ‘not particularly attractive’, ‘people who bore you’, pessimism concerning outcomes, feelings of the work being unrewarding and lack of confidence in their management skills especially, but not exclusively, in PNs.* |
|  |  |  | Ju (2018) | Barriers  *When patients were seen to lack motivation and had ‘no intention of doing anything’, some GPs perceived that their efforts to encourage the patient’s uptake of prevention strategies were a ‘waste of time’. In failing to motivate patients, GPs questioned their ability to prevent CVD in their patients, being ‘[un]convinced*  *that we do as much good as we like to think we do’.* |
|  |  |  | Sirdifield (2013) | Barriers  *Scepticism regarding nonpharmacological approaches to the treatment of conditions such as anxiety and insomnia was expressed by the physicians interviewed. They identified common mild alternatives (e.g. warm milk, not watching violent movies before bed) and considered them to be ineffective for elderly people with chronic problems and thought that psychotherapeutic approaches were “doomed to failure”. Thus, the decision to prescribe medication was often seen as the most effective way to help the patient.* |
|  |  |  | Yeung (2015) (2015) | Barriers  *Negative attitudes towards chlamydia from GPs and senior personnel precipitated a lack of enthusiasm in the rest of the staff.* |
| **Beliefs about consequences** | **Consequences of prescribing** |  | Ju (2018) | Factors  *In patients with comorbidities (eg, diabetes, mental illness), some GPs chose to delay prescribing strategies for CVD prevention to minimise the stress in patients of having to contend with multiple treatments.*  *They believed that giving young patients or patients who were not at high risk a lifetime prescription of medicine for preventive purposes should be avoided by encouraging lifestyle changes instead, to prevent a dependence on medications when it was not absolutely necessary.* |
|  |  |  | Lucas (2015) | Barriers  ***Concerns regarding adverse effects and antibiotic resistance discouraged clinicians from prescribing, as did concerns regarding over-prescription in general. Where clinicians did not feel there was pressure from parents to give a prescription, this enabled them to avoid prescribing antibiotics.*** |
|  |  |  | Sinnott (2013) | Factors  *The impact of treatment burden was an important consideration given the greater costs and risk of adverse drug events associated with the use of multiple medications.* |
|  |  |  | Sirdifield (2013) | Factors  *GPs varied in their estimation of the balance of adverse drug effects, including risk of addiction/abuse, against potential benefits for older patients: “In the end, physicians believed that the advantages of continuing benzodiazepines in the elderly outweighed the problems”.* |
|  |  |  | Tonkin-Crine (2011) | Factors  *Many may feel uncertain about potential illness consequences when not prescribing and this may lead to inappropriate prescribing as a way to ensure that patients are protected.*  *Prescribing may also be affected by whether GPs feel resistance is important, if their own prescribing contributes to this and how well they know recent research evidence. Some may mistakenly believe that their prescribing matches recommendations, when it does not.* |
|  |  |  | Zwolsman (2012) | Barriers  ***A main barrier is the applicability of evidence in general practice. The difference between primary care patients and the patients in the research population of secondary care is mentioned as a reason for this, which relates to the fact that research from clinical trials cannot be generalised to patients in general practice. As a result, GPs fear possible harm or side effects.*** |
|  | **Futility** |  | Ju (2018) | Factors  *Disappointment with futility of advice. When patients were seen to lack motivation and had ‘no intention of doing anything’, some GPs perceived that their efforts to encourage the patient’s uptake of prevention strategies were a ‘waste of time’. In failing to motivate patients, GPs questioned their ability to prevent CVD in their patients, being ‘[un]convinced that we do as much good as we like to think we do’.* |
|  |  |  | Mikat-Stevens (2015) | Barriers  *Furthermore, the perception that genetics was less prevalent or significant than other issues was identified as a barrier in eight studies. This included the notion that genetics was not perceived to have an impact on primary-care practice.*  *The most commonly cited barrier was a perceived lack of therapeutic interventions available after the identification of increased risk (n = 11 cited in 11/38 studies)24,27,28,36,39 and the notion that patient management would not change as a result of genetic assessment.* |
|  |  |  | Schumann (2012) | Barriers  *FPs are reluctant to use diagnostic labels prematurely because a speciﬁc diagnosis has few consequences for treatment or prognosis especially in minor and subthreshold depression.* |
|  |  |  | Vogt (2005) | Barriers  ***Not effective. Statements referring to physicians' expectations about whether discussing smoking would result in smoking cessation.*** |
|  |  |  | Vedel (2011) | Barriers  *The PCPs' lack of belief in the value of screening was identified as the main barrier influencing the decision to proceed.*  Facilitators  ***Other major facilitators were linked to perception of cancer screening tests (8 studies): belief in screening usefulness and simplicity and safety of the test.*** |
|  | **Medicalisation** |  | Barley (2011) | Barriers  ***Clinicians holding a ‘normalising’ understanding of depression found it difficult to distinguish between distress and depression and worried about medicalising social problems.***  ***Among some clinicians, ambivalent attitudes to working with depressed people, a lack of confidence, the use of a limited number of management options and a belief that a diagnosis of depression is stigmatising complicate the management of depression.*** |
|  |  |  | De Vleminck (2013) | Barriers  *Most patients and professionals agree that talking about ACP should take place around the time of diagnosis of a life-threatening illness, but fear of depriving patients of hope is a barrier preventing GPs from initiating ACP for which stronger evidence was found.* |
|  |  |  | Ju (2018) | Barriers  ***Some GPs were cautious and critical of ‘medicalizing an unhealthy lifestyle’ as this encouraged patients to continue with their harmful habits (eg, sedentary lifestyle, poor diet, smoking) and ‘forget about their lipid-lowering diet’.***  *Regardless of the patient’s level of risk for CVD, some GPs urged to avoid instilling unnecessary anxiety in patients, as ‘fear becomes a major problem’ and in turn elevates their risk further.* |
|  |  |  | Mikat-Stevens (2015) | Barriers  *Several issues related to prenatal ethics arose, including the perception that prenatal genetic testing dampens the natural excitement of pregnancy or could medicalize pregnancy and a fear that prenatal and preconception genetic testing would motivate individuals to want to create a “perfect child,” which might create additional disparities in society regarding individuals with genetic conditions or disabilities.* |
| **Reinforcement** | **Incentives** |  | De Vleminck (2013) | Facilitators  *There is medium evidence for the time available, and the chances of reimbursement, being facilitators.* |
|  |  |  | Ju (2018) | Barriers  *Some GPs in studies conducted in the UK and New Zealand were careful not to exceed their budget for drug prescriptions, and they were conscious of the limitations of funding available for their practice, which contended with external pressures (from pharmaceutical companies, health advertising) to offer drug treatment.* |
|  |  |  | Lawrence (2016) | Barriers  ***Lack of remuneration and inadequate funding.*** |
|  |  |  | Lucas (2015) | Facilitators  *US based clinicians said incentives & free samples from pharmaceutical industry encouraged prescription.* |
|  |  |  | McDonagh (2018) | Barriers  *Targets set too high- testing targets perceived to be unachievable can result in a practice disengaging from testing, and realistic targets need to be set, reflecting the area (e.g., rural, urban) in which a practice is located.*  Facilitators  ***Some PCPs interviewed suggested that having an incentive programme would help testing become a priority and other PCPs indicated that they would increase testing if offered incentive payments for each test performed. This was consistent with a drop in testing when previously offered practice incentives were removed.*** |
|  |  |  | O’Brien (2016) | Barriers  *Difficulties gaining insurance reimbursement for mental health diagnoses.*  Facilitators  *Increased reimbursement possible facilitator that could increase ‘behavioural health’ diagnoses.* |
|  |  |  | Schadewaldt (2013) | Barriers  *Nurse practitioners and MPs strongly perceived that economic constraints had a negative impact on collaborative practice. The lack of financial support for the NP role often made employment of a NP not financially viable for a practice setting. There was a perception that the health care system did not sufficiently reimburse NP services.* |
|  |  |  | Sirdifield (2013) | Facilitators  *The use of heuristics together with contextual limitations in terms of short consultation times, remuneration for treatment in some countries et cetera may lead to long-term prescribing contrary to clinical guidelines.* |
|  |  |  | Tonkin-Crine (2011) | Facilitators  *GPs welcome interventions that beneﬁt their practice either by decreasing workload or offering ﬁnancial gain.* |
|  |  |  | Vedel (2011) | Barriers  *Other frequently reported barriers were linked to the type of medical practices (8 studies): lack of routine general examination, lack of incentive fees and solo or rural practice.* |
|  |  |  | Yeung (2015) (2015) | Facilitators  ***Financial incentives were mentioned by GPs as a way to increase testing but questions remain about its effectiveness.*** |
|  |  |  | Zwolsman (2012) | Barriers  *A lack of investment by health authorities is particularly described in two survey studies. Some GPs consider EBM not to be cost effective for themselves as practitioners, and feel they require additional financial resources for the facilities needed when using EBM. To them, seeing patients is more cost effective than spending time in the field of EBM, since, in their opinion, time per patient increases when EBM is used. For GPs, there is no financial gain in using EBM, because time spent on EBM is not paid for.*  *Lack of investment/incentives.* |
|  | **Feedback** |  | McDonagh (2018) | Facilitators  *Regular feedback helped personally motivate PCPs and facilitate the embedding of chlamydia testing into general practice.* |
|  |  |  | O’Brien (2016) | Facilitators  *Desire for increased communication, information, and feedback on referrals.* |
|  |  |  | Tonkin-Crine (2011) | Facilitators  *Personal or local feedback allows an opportunity for GPs to reﬂect on their own prescribing and potential effects, and offers motivation to change.* |
| **Goals** |  |  | Ju (2018) | Facilitators  *When developing a strategy for preventing CVD, some GPs perceived that compromise was necessary in encouraging patients to cooperate. An explicit discussion and consideration of the patient’s goals and priorities was seen to encourage patients to ‘work with the doctor, not against the doctor’ which built trust. Some GPs coproduced a strategy with the patient that was feasible for the patient’s own situation.* |
|  |  |  | Schadewaldt (2013) | Facilitators  *While having complementary skills and similar goals was seen as an asset to collaboration, ideological differences in the practice style could cause difficulties in establishing a collaborative relationship.* |
|  |  |  | Sinnott (2013) | Facilitators  *Delivering patient-centred care This domain emerged as an intuitive and over-riding goal of GPs in all studies, and interventions in multimorbidity must help GPs deliver on this aspiration.* |
| **Memory, attention and decision processes** | **Decision processes** |  | Barley (2011) | Facilitators  *Some clinicians’ encouraged patients to understand depression as biochemical even when they themselves did not hold this view. Their aims in doing so were to: ’clarify the experience of depression, remove blame and stigma and to provide a way forward to use antidepressants’.* |
|  |  |  | Ju (2018) | Barriers  *Our findings indicate that GPs may prefer to make their own judgement of individual risk factors acquired through experience rather than using absolute risk assessment tools.* |
|  |  |  | Lucas (2015) | Factors  ***Across these studies clinicians described limited occasions when they were certain about the prescription decision (either to prescribe or not to prescribe). More often, clinicians reported prescribing when uncertainty existed either because of the lack of diagnosis, or uncertainty regarding the social, health, or legal consequences of not prescribing and therefore took a “just in case” approach.*** |
|  |  |  | Schumann (2012) | Factors  ***Depending on their individual attitudes, FPs used different approaches to diagnose depression. Throughout 5 of the 13 studies, FPs mentioned a ‘ruling out physical symptoms’ and ‘uncovering symptoms’ strategy.*** |
|  |  |  | Sinnott (2013) | Factors  *GPs used modiﬁed approaches to guidelines, involving, for example, the estimation of risk associated with particular diseases/treatments. However, some felt that this modiﬁcation was in conﬂict with ‘best practice’ and felt guilt at not implementing guidelines fully.* |
|  |  |  | Sirdifield (2013) | Factors  *GPs often managed the tension between minimising prescribing and their responsibility to help patients on a case-by-case basis. They needed to justify giving or withholding benzodiazepines, expressed in the literature through the concept of the ‘deserving patient’.*  ***GPs felt a desire and responsibility to help their patients and decisions about what form this should take (i.e. a prescription or a non-pharmacological alternative) was based on competing and sometimes contradictory factors.*** |
|  |  |  | Tonkin-Crine (2011) | Factors  *One ﬁnal theme, distinct from those above, was related to GPs’ satisfaction with their prescribing decisions. GPs appear more satisﬁed when all factors inﬂuencing their choice are in agreement. For example, when guidelines recommend not giving an antibiotic to a patient who wants one, a GP may not feel satisﬁed about a decision either way. Alternatively, a GP may choose to prioritize a patient’s wishes over guideline advice, or vice versa, and feel satisﬁed after having made a choice.* |
|  | **Memory** |  | Barley (2011) | Barriers  *GPs may be less likely to refer older patients for psychological therapy, either because they ‘forget’ about it or assume it will not work in this population.* |
|  |  |  | McDonagh (2018) | Barriers  ***Forgetfulness In some studies, PCPs only remembered to test when patients attended for other related health issues (e.g., contraception) or revealed high-risk behaviours. Other PCPs remembered at the start of a trial or screening programme but forgot over time and lack of a formal recall/reminder system to help staff remember was a barrier.*** |
|  |  |  | Yeung (2015) (2015) | Barriers  *The act of offering a test to the patient was easy to forget, particularly as time increased between the reminder and offer of a test, and was attributed in one study investigating opportunistic testing with low uptake.*  Facilitators  *Facilitators identiﬁed at the GP level included remembering to test/normalisation of testing, education/awareness/training and incentives.* |
| **Environmental context and resources** | **Time, workload and general resources** |  | Barley (2011) | Barriers  *Listening requires time; a lack of time was reported in several studies but was refuted by one.* |
|  |  |  | Carlsen (2007) | Barriers  ***In most of the studies, GPs referred to a lack of time t******o read and assess the guidelines, follow the recommendations, and negotiate with patients, leading Langley and colleagues to refer to GPs’ ‘white rabbit persona’. Other practical constraints, including convenience, lack of skills with new procedures, and lack of resources, were also referred to.***  *Some GPs perceived preventive procedures (blood tests, routine checks) to be a healthcare burden when the whole population was screened regardless of risk levels or immediate illnesses. This placed them under increasing pressure due to a greater demand for general screening. They were mindful of the resources and nurse time as well as their own time spent screening for risks for primary prevention in low-risk patients, as this detracted from resources available for patients who were ‘actually ill’.* |
|  |  |  | De Vleminck (2013) | Barriers  *The limited resources available in primary care were perceived as a barrier.*  *Limited resources available to honour patients ’ or families ’ expectations.*  Facilitators  *There is medium evidence for the time available, and the chances of reimbursement, being facilitators.* |
|  |  |  | Ju (2018) | Barriers  *Some GPs were enthusiastic about a team-based approach to prevention involving trained practice nurses and lifestyle advisors due to time constraints in their own consultations.*  *They were mindful of the resources and nurse time as well as their own time spent screening for risks for primary prevention in low-risk patients, as this detracted from resources available for patients who were ‘actually ill’.* |
|  |  |  | Lawrence (2016) | Barriers  ***Lack of time identified as barrier.***  *Lack of general resources identified as barrier.* |
|  |  |  | Lucas (2015) | Barriers  ***Clinicians needed a consultation that was quickly completed and where both parents and clinician were satisﬁed with the outcome. They also wanted to educate parents to understand that antibiotics were not necessary, but found this difﬁcult to achieve. The pressure to keep consultations short meant some gave in order to bring consultations to a rapid conclusion.***  ***Requirement to end consultation quickly.*** |
|  |  |  | McDonagh (2018) | Barriers  ***PCPs reported that consultation length was insufficient to allow testing, in addition to discussing the primary consultation reason and other priority issues. Testing requires time to discuss sexual health,***  ***gain permission, and raise partner notification.***  *There were concerns about funding and remuneration for the expansion of PN roles, increases in workload, and time constraints within consultations.*  *Both groups experienced the allocated time for consultations as a competing pressure, and PCPs struggled to reconcile the need to discuss the relevance of testing to young people given the workload this would create, whatever the model for test offer.*  Facilitators  *Additional time in consultation.*  *Self-taken and non-invasive sampling is more acceptable to patients and reduces workload for PCPs, thereby facilitating testing.* |
|  |  |  | Mikat-Stevens (2015) | Barriers  ***The second most frequently cited systems barriers related to time (n = 21 cited in 18/38 studies). This included time needed to collect detailed FHs as part of the primary-care visit and having insufficient time to explain the results of genetic tests to patients. In the case of genetic medicine in prenatal care, the time to obtain results was reported as being too long to inform any treatment decisions.*** |
|  |  |  | O’Brien (2016) | Barriers  ***Reimbursement, a lack of insurance coverage, time restrictions, and a lack of providers and resources posed significant barriers to primary care practitioners’ management of child and adolescent mental health problems.***  Facilitators  *Collaboration with other professionals and increased providers and resources.* |
|  |  |  | Ogeil (2020) | Barriers  ***The present analysis demonstrates that the primary care environment itself makes it difficult for general practitioners to assess sleep. Clinicians are often ‘time poor’ during consultations, may be presented with a lengthy list of complaints, and/or be reluctant to address sleep complaints, especially if they are rated as less important than other symptoms.*** |
|  |  |  | Schadewaldt (2013) | Barriers  *Regular meetings & time to collaborate.*  *A high level of NP autonomy was a crucial component to collaboration, because limitations in the NP’s autonomy; in particular their inability to prescribe or order diagnostic tests was found to increase the MPs workload and consequently negatively influence collaborative practice.* |
|  |  |  | Schumann (2012) | Barriers  ***Most of the FPs considered the process of diagnosing depression as time consuming and needing multiple contacts.***  ***Some FPs mentioned that the assumption of depression would affect the whole schedule and that the knowledge that diagnosing depression needs time could have an impact on the willingness to diagnose this mental disorder.*** |
|  |  |  | Sinnott (2013) | Barriers  *Insufﬁcient consultation time led to amended or suboptimal approaches in many cases. It was suggested that weighting consultation lengths to the complexity of multimorbidity would facilitate more effective management.* |
|  |  |  | Sirdifield (2013) | Barriers  **Time limited consultations (a barrier to alternative interventions), or the prospect of unhappy patients leaving for other practices, influenced whether or not GPs initiated withdrawal.**  **Making decisions on whether or not to prescribe was often uncomfortable, demanding and complex within the time and pressure constraints of daily practice.** |
|  |  |  | Tonkin-Crine (2011) | Barriers  *It is primarily lack of time that leads the physician to lower his/her threshold of tolerance, a prescription may in such cases be the quickest option.*  Facilitators  *GPs welcome interventions that beneﬁt their practice either by decreasing workload or offering ﬁnancial gain.* |
|  |  |  | Vedel (2011) | Barriers  ***Other barriers were the lack of resources within the practice (7 studies): lack of human resources or equipment, lack of information system, no reception of the test kits by patients.***  ***Moreover, the lack of external testing resources has been identified as a major barrier.***  ***PCPs' lack of time has also been reported as a barrier to cancer screening; busy physicians may not have enough time to apply all preventive recommendations.***  Facilitators  ***Another facilitator was the accessibility of screening test (7 studies): health care coverage for test, external resources.***  *Another facilitator was the presence of resources within the practice (3 studies): presence of facilities to perform the test and use of information systems.* |
|  |  |  | Vogt (2005) | Barriers  ***The most common negative belief or attitude was that discussing smoking was too time-consuming.***  Facilitators  *Increasing the amount of time may increase the frequency with which smoking is discussed, but it is clear that time is not the only predictor of whether smoking is discussed.* |
|  |  |  | Yeung (2015) (2015) | Barriers  ***Lack of time and a heavy workload were the most cited barriers to chlamydia testing and addressing sexual health-related concerns. More than half of the GPs identiﬁed time as an issue. These were linked with administrative issues related to the testing of chlamydia and management of a diagnosis. A standard consultation did not allow sufﬁcient time to introduce testing, particularly in a patient with limited knowledge, or in an unrelated consultation.***  *Inadequate supplies of promotional materials were also recognised as barriers to testing. Few informational leaﬂets were provided in waiting rooms, or they were out-of-date or not replenished.* |
|  |  |  | Zwolsman (2012) | Barriers  ***The barrier relating to the busy workload in general practice is expressed in the time that is available to GPs. Time for using the concept of EBM is described in many studies as a barrier. A shortage of time during consultations does not allow GPs to search for, or access, evidence, and they do not have time to reflect on their clinical practice. Time to search for and appraise articles is specifically mentioned in a qualitative study as an important barrier: GPs in this study consider time a more important barrier than lack of skills.***  ***Access is the barrier most mentioned. It can be subdivided into lacking resources, no access to evidence, and computer- or internet-related problems, and therefore mainly entails technical difficulties.*** |
|  | **Guidelines, evidence and decision-making tools** |  | Barley (2011) | Barriers  *That GPs and PNs are aware of the relationship between social and mood problems is clear from this review, but they are unsure of its exact nature and of their role in managing it. This uncertainty may be exacerbated by a lack of attention in guidelines concerning the influence of social problems on response to treatment.* |
|  |  |  | Carlsen (2007) | Barriers  ***Questioning the guidelines This theme occurred in all studies and contained a number of linked sub-themes. Studies indicated that some GPs were sceptical about the evidence base for guidelines, for example, they argued that population-based trials were not necessarily applicable to individual patients. GPs also pointed out that the use of narrow inclusion criteria could weaken the applicability of the evidence from trials.***  ***All of the studies report that GPs described a tension between their own experiences and the guideline recommendations, and that GPs saw consultations with real patients as more complicated than their portrayal in the guidelines. Guidelines were experienced as not flexible enough to take into account the complexity of individual circumstances, such as multiple diagnoses, painful side-effects, and patient preference.***  ***GPs expressed uncertainty about the evidence base in the face of changes over time and controversies. They also suggested that there could be a conflict between the aims of the guidelines and the motivations of GPs; the former relating to cost containment, the latter relating to patient care.***  ***Some studies also referred to the guideline format as an important determinant of GPs’ attitudes. There was some consensus that guidelines needed to be short and simple and include patient leaflets.*** |
|  |  |  | Ju (2018) | Barriers  *Some GPs considered the patient’s family history and background when determining prevention strategies. They advocated the use of ‘human judgement,’ which incorporated ‘emotional, political and logistical’ considerations rather than accepting risk scores unconditionally. Others were unwilling to use risk scores to estimate pretreatment risk due to ambiguity of current guidelines regarding unique patient circumstances.*  Facilitators  *Some GPs trusted research evidence and expert opinion to feel secure about their decisions. Guidelines, risk assessment tools and ‘editorials in the BMJ were seen to minimise room for human error and were more reliable than their own judgement.* |
|  |  |  | Lawrence (2016) | Barriers  ***PCPs commonly reported that primary-care guidelines were not well defined or consistent for cancer survivors, and that there was a need for timely, detailed and regular transfer of information.***  ***inadequate resources including cancer-specific guidelines and opportunities for mental health or multi-disciplinary referrals.*** |
|  |  |  | McDonagh (2018) | Barriers  *Many of the barriers faced by practice staff, such as lack of knowledge and discomfort in discussing testing with patients, relate to lack of guidance, for example, clarity on when and how to test asymptomatic patients.*  Facilitators  *In one Australian study, over 90% of GPs indicated that they would be likely to increase testing if national testing guidelines were introduced and enforced. In some cases, increasing awareness of existing guidelines could facilitate testing.* |
|  |  |  | Mikat-Stevens (2015) | Barriers  *The second most frequently cited evidence barrier reported was the lack of guidelines and insufficient evidence to support risk assessment (n = 10 cited in 10/38 studies).* |
|  |  |  | O’Brien (2016) | Barriers  *Absence of a ‘gold standard’ for dealing with children’s mental health problems, specifically pinpointing ‘unhelpful’.*  *Desire for clearer referral criteria — Child and Adolescent Mental Health Services criteria were described as a ‘mystery’.* |
|  |  |  | Schumann (2012) | Barriers  *The synthesis revealed that a large number of FPs express doubts about the validity of the diagnostic concept of depressive disorders used in the DSM/ICD and practice guidelines, especially for primary care. They seem to perceive that the current standard of diagnostic criteria deﬁnes depressive disorders too much on the basis of symptom counts.* |
|  |  |  | Sinnott (2013) | Barriers  ***There was concern among GPs about clinical guidelines, which are ‘generally written for sole conditions’ and do not account for ‘the unique circumstances of each patient’. Most GPs felt that guidelines were less useful in multimorbidity and that they actually added to the complexity in some cases: ‘no one can tell you the added beneﬁt of an additional agent for blood pressure if you are already on ten’.***  ***The inadequacy of guidelines and evidence-based medicine: Guidelines offer GPs less support in the management of multimorbid patients and may in fact cause additional problems when they try to adhere to them.*** |
|  |  |  | Sirdifield (2013) | Factors  *GPs perceived the context for benzodiazepine prescribing decisions had changed over time because of changing: norms of practice, evidence, guidance (national and local), introduction of new drugs (e.g. selective serotonin reuptake inhibitors) and services, legal regulatory frameworks and the societal attitudes for treatment of conditions including anxiety, depression and insomnia.* |
|  |  |  | Tonkin-Crine (2011) | Barriers  *GPs’ uncertainty about ARTI guidelines changes in guidelines or recommendations may cause uncertainty about ARTI management for GPs.*  Factors  ***Perceptions of external pressure to reduce prescribing Although guidelines can be viewed as helpful, some GPs may not always trust information and may disregard it when making decisions.*** |
|  |  |  | Vedel (2011) | Barriers  *Other reported barriers are linked to the lack of knowledge (7 studies): lack of awareness about cancer screening, lack of skills, lack of training, lack of clear guidelines.*  Facilitators  *The facilitators to screening most commonly identified by PCPs were linked to physicians' knowledge (10 studies): knowledge/awareness of screening and cancer, skills, training, existing useful guidelines.* |
|  |  |  | Yeung (2015) (2015) | Facilitators  *The normalisation of testing may prove to be the most important facilitator of chlamydia testing. It is the cumulative product of many minor changes, which may be assisted from external sources, such as national guidelines or government programs.* |
|  |  |  | Zwolsman (2012) | Barriers  ***Most of the 22 articles describe barriers that are related to the evidence itself. Some say that general practice lacks sound evidence, especially for the many problems faced by GPs. In one study sample, 34% of the surveyed GPs felt that there is a lack of evidence. Other studies found that the available evidence is perceived to be of inadequate quality. On the other hand, too much available evidence is also experienced as a problem. Furthermore, GPs say that the available evidence is contradictory, not up to date, and liable to time delays. Time delays mainly appear between the publication of research and eventual adjustment of practice.***  ***A main barrier is the applicability of evidence in general practice. The difference between primary care patients and the patients in the research population of secondary care is mentioned as a reason for this, which relates to the fact that research from clinical trials cannot be generalised to patients in general practice. As a result, GPs fear possible harm or side effects.***  ***The accessibility of literature written in English is described as a problem for non-English GPs, as is the understanding of the English in which articles are written and the lack of evidence published in the GP’s own language.*** |
|  | **Financial resources and insurance coverage** |  | Carlsen (2007) | Barriers  *Some GPs in studies conducted in the UK and New Zealand were careful not to exceed their budget for drug prescriptions, and they were conscious of the limitations of funding available for their practice, which contended with external pressures (from pharmaceutical companies, health advertising) to offer drug treatment.*  Factors  *In making decisions about prescribing medication therapy, they considered the economic impact on their local practice (particularly in the UK) and broader healthcare costs, and specifically in terms of prioritising resources for patients with more urgent illnesses than to those who were asymptomatic with risk factors.* |
|  |  |  | Ju (2018) | Barriers  ***Some GPs especially in low socioeconomic regions like Guatemala were mindful of the economic burden of long-term medication on patients and thus prescribed medications only for patients at high risk as determined by their cholesterol or blood pressure.*** |
|  |  |  | Lawrence (2016) | Barriers  ***Insufficient budget or increased cost identified by 79–83 % of PCPs.***  ***Poor remuneration identified as barrier.*** |
|  |  |  | McDonagh (2018) | Barriers  *Limited funding for testing meant staff were less likely to offer tests in comparison to paid for public health interventions.*  Facilitators  *Some PCPs interviewed suggested that having an incentive programme would help testing become a priority and other PCPs indicated that they would increase testing if offered incentive payments for each test performed. This was consistent with a drop in testing when previously offered practice incentives were removed.* |
|  |  |  | Mikat-Stevens (2015) | Barriers  *Cost to both patients and providers was expressed as a barrier to integration of genetics services into primary care. This included a barrier in regard to the lack of insurance coverage for genetic tests (n = 6 citations), as well as the cost to the patient (n = 10 citations).* |
|  |  |  | O’Brien (2016) | Barriers  ***Reimbursement, a lack of insurance coverage, time restrictions, and a lack of providers and resources posed significant barriers to primary care practitioners’ management of child and adolescent mental health problems.***  Facilitators  *Increased reimbursement possible facilitator that could increase ‘behavioural health’ diagnoses.* |
|  |  |  | Schadewaldt (2013) | Barriers  ***Nurse practitioners and MPs strongly perceived that economic constraints had a negative impact on collaborative practice. The lack of financial support for the NP role often made employment of a NP not financially viable for a practice setting. There was a perception that the health care system did not sufficiently reimburse NP services.***  Facilitators  ***Financial support for NP role.*** |
|  |  |  | Schumann (2012) | Barriers  *Barriers resulting from the health system or society encompassed fear of stigmatization and (premature) labelling, future insurability, reimbursement, and lack of time and resources.*  *The societal stigma and fear of premature labelling and a possible impact on future insurability and reimbursement led clinicians to rule out other possible medical causes ﬁrst before considering the possibility of a mental illness.* |
|  |  |  | Tonkin-Crine (2011) | Factors  *GPs’ perception of potential ﬁnancial beneﬁts that can be gained from prescribing antibiotics may affect the prescribing decision.*  Facilitators  *GPs welcome interventions that beneﬁt their practice either by decreasing workload or offering ﬁnancial gain. Examples may include fewer reconsultations as a result of improved communication skills or the provision of ﬁnancial incentives.* |
|  |  |  | Vedel (2011) | Barriers  ***PCPs and older adults reported the lack of health care coverage for test as a major barrier to cancer screening for older adults.***  ***Other frequently reported barriers were linked to the type of medical practices (8 studies): lack of routine general examination, lack of incentive fees and solo or rural practice.*** |
|  |  |  | Yeung (2015) (2015) | Barriers  *Several logistical issues were also presented as barriers to chlamydia testing. Inadequate funding, resources and services for testing contributed to an inability to test, including difﬁculties in referring a patient for further counselling or not having sufﬁcient or appropriate printed resources.* |
|  |  |  | Zwolsman (2012) | Barriers  ***A lack of investment by health authorities is particularly described in two survey studies. Some GPs consider EBM not to be cost effective for themselves as practitioners, and feel they require additional financial resources for the facilities needed when using EBM.***  Facilitators  *The relatively uncommon finding of this review is that GPs feel a need for incentives for working in an evidence-based manner, because the infrastructure that is needed for EBM requires financial input, practising EBM requires time that is not reimbursed.* |
|  | **Education and training** |  | Barley (2011) | Barriers  *It is also because of findings that a negative past experience of mental health training was associated with PNs’ current negative attitudes towards engaging with patients’ mental health needs.*  Facilitator  *GPs’ diagnoses were more accurate if they felt confident treating depression and more recently trained nurses believed a higher proportion of their patients to be depressed.*  *A consistent recommendation was that training should involve consideration of professionals’ views and attitudes towards depression as these impact on clinical decision making.* |
|  |  |  | Lawrence (2016) | Barriers  *However, lower levels of training or knowledge were reported for areas such as surveillance testing, active treatment or surgery and advising on complementary therapies, leading to a reluctance to fulfil such roles.*  *Commonly endorsed barriers to PCP delivery of cancer care included lack of expertise or formal training.* |
|  |  |  | McDonagh (2018) | Barriers  ***Lack of training and skills: PCPs reported a lack of appropriate training and skills needed to discuss sexual health, take sexual history, offer a test, respond to a positive test and manage treatment, and conduct partner notification. This led to reduced confidence to offer testing (reflective motivation) and discuss sexual health.***  *Many PCPs felt they did not have the necessary support for partner notification and expressed uncertainty about how it worked, indicating a need for skills-based training.*  Facilitators  *PCPs were willing to conduct testing if trained and GPs with training in STIs were more likely to offer testing.*  *GPs with postgraduate education in STIs were more willing to offer testing to men as well as indicating greater knowledge of the need to offer to both men and women.*  *Skills-based training and increasing psychological knowledge could facilitate testing by increasing confidence in offering tests.* |
|  |  |  | Mikat-Stevens (2015) | Barriers  *Need genetic education (predictive genetic testing).* |
|  |  |  | O’Brien (2016) | Barriers  *Lack of training must be addressed as a high priority.*  *Lack of emphasis on mental health in medical training.* |
|  |  |  | Ogeil (2020) | Barriers  ***We found from the clinician perspective that there are barriers related to: Knowledge or an awareness that insomnia is a significant issue in many cases, and/or a lack of training to identify insomnia as distinct from other sleep disorders.***  Facilitators  *Greatest influence on changing practice style regarding sleep were journal articles followed by continuing education, followed by discussion with specialists.* |
|  |  |  | Schadewaldt (2013) | Barriers  *Concern about: NP education and competence.*  *Some MPs stated that they considered the difference of education between NPs and MPs as a barrier to acceptance of NPs as equal partners.* |
|  |  |  | Sirdifield (2013) | Barriers  *GPs also feel uncertain how to deal with psychosocial problems, as a result of insufficient training.* |
|  |  |  | Tonkin-Crine (2011) | Facilitators  ***Interventions may educate GPs about appropriate prescribing GPs value additional education if they perceive that it comes from a knowledgeable and trustworthy source.*** |
|  |  |  | Vedel (2011) | Barriers  *Other reported barriers are linked to the lack of knowledge (7 studies): lack of awareness about cancer screening, lack of skills, lack of training, lack of clear guidelines.* |
|  |  |  | Vogt (2005) | Facilitators  *Training physicians can result in more positive beliefs and attitudes towards discussing smoking cessation with patients, increase self-efficacy to discuss smoking cessation as well as increase frequencies of discussing smoking cessation.* |
|  |  |  | Yeung (2015) (2015) | Barriers  ***Another signiﬁcant barrier was the lack of knowledge, skill, training and awareness around chlamydia testing.***  ***Investigating sexual health was not taught well in medical school, and many GPs were unaware of the epidemiology, presentation or testing procedures for chlamydia.***  Facilitators  *Education was instrumental to helping GPs identify the need for testing, and feel confident in offering a test or taking a sexual history, although this effect is not sustained.* |
|  |  |  | Zwolsman (2012) | Barriers  ***Lack of knowledge and skills also influences GPs’ use of EBM. 55.6%feel that training in EBM is required, and that there is a lack of such training.*** |
|  | **Availability of and access to specialist services** |  | Barley (2011) | Barriers  *Specialist services Secondary care psychiatry or psychology, voluntary services and social care services were considered good quality, but provision and/or access to them was commonly considered inadequate. Lack of access to external services was seen as more of an obstacle to providing effective treatment of depression than personal knowledge or skill.* |
|  |  |  | Lawrence (2016) | Barriers  *Inadequate access to mental health referrals for providing survivorship care identified by 46 % of PCPs.*  Facilitators  *PCPs considered the advantages of primary-cared based follow-up to include: pre-existing relationships with patients (82 %), accessibility (69 %) and lower costs (66 %)*. |
|  |  |  | Mikat-Stevens (2015) | Barriers  ***The largest category of systems barriers was lack of access to genetics services (n = 23 citations in 17/38 studies). This encompassed specific barriers related to lack of access to genetics services in general, and a specific lack of access to genetic counselors or geneticists.*** |
|  |  |  | O’Brien (2016) | Barriers  ***A lack of providers of specialist services was the most highly endorsed barrier overall.***  Facilitators  *Other barriers that were specific to particular stages included a lack of confidence in identification and diagnosis, along with long waiting times when referring children to specialist services (a reduction in which was the most highly endorsed facilitator overall).* |
|  |  |  | Schumann (2012) | Barriers  *Even if the patient were to accept the diagnosis, the FPs believed that time and resources available for dealing with depression were limited. Patients and FPs were confronted with long waiting lists for specialized mental health care.* |
|  |  |  | Sinnott (2013) | Barriers  ***Disorganisation and fragmentation of healthcare: The involvement of multiple specialists and the emphasis on single disease care is antagonistic to the ‘holistic’ goals of GPs.*** |
|  |  |  | Sirdifield (2013) | Factors  *GPs perceived the context for benzodiazepine prescribing decisions had changed over time because of changing: norms of practice, evidence, guidance (national and local), introduction of new drugs (e.g. selective serotonin reuptake inhibitors) and services, legal regulatory frameworks and the societal attitudes for treatment of conditions including anxiety, depression and insomnia.* |
|  |  |  | Vedel (2011) | Barriers  ***The most often barrier identified by both PCPs and patients was the lack of accessibility to screening tests (16 studies).***  Facilitators  ***Another facilitator was the accessibility of screening test (7 studies): health care coverage for test, external resources.*** |
|  |  |  | Yeung (2015) (2015) | Barriers  *Inadequate funding, resources and services for testing contributed to an inability to test, including difﬁculties in referring a patient for further counselling or not having sufﬁcient or appropriate printed resources.* |
|  |  |  | Zwolsman (2012) | Barriers  *Lack of help in interpretation (of evidence) by experts/specialists.* |
|  | **Diagnostic and management options** |  | Barley (2011) | Barriers  ***However, concern about stigmatisation may be constructed to hide a reluctance to explore depression with patients arising from a desire to avoid feelings of powerlessness when management options seem limited.***  ***Professionals may be reluctant to diagnose depression if they feel they have nothing to offer the patient.***  ***Among some clinicians, ambivalent attitudes to working with depressed people, a lack of confidence, the use of a limited number of management options and a belief that a diagnosis of depression is stigmatising complicate the management of depression.*** |
|  |  |  | Ju (2018) | Barriers  *Some GPs perceived preventive procedures (blood tests, routine checks) to be a healthcare burden when the whole population was screened regardless of risk levels or immediate illnesses.* |
|  |  |  | McDonagh (2018) | Barriers  *Mode of testing “I think the whole procedure of taking swabs is a bit of a turn-off really”.*  Facilitators  *Mode of testing. Self-taken and non-invasive sampling is more acceptable to patients and reduces workload for PCPs, thereby facilitating testing.* |
|  |  |  | Mikat-Stevens (2015) | Barriers  *The most commonly cited barrier was a perceived lack of therapeutic interventions available after the identification of increased risk (n = 11 cited in 11/38 studies) and the notion that patient management would not change as a result of genetic assessment.*  *Furthermore, PCPs reported that limitations of current genetic screening tests deterred them from utilizing them in practice (n = 10 cited in 7/38 studies). Specific limitations of genetic tests were inaccurate or unambiguous results, a high rate of false positives (in cancer risk testing), and concern over the validity of genetic testing.* |
|  |  |  | Schumann (2012) | Barriers  *FPs stated that there is no clear diagnostic test such as a blood test to conﬁrm the diagnosis as in other somatic diseases. ‘Depression is messy as it is unlike other medical conditions for which there are objective tests and measurable treatment outcomes’.*  Facilitators  *Instead, the FPs mentioned that ‘once a level of suspicion has been established with familiar patients, they could raise the possibility of a mental health diagnosis by asking the few screening questions they have found to be useful rather than going through the ICD10/DSM list of symptoms’. Others found it helpful to use simple checklists/handouts or questionnaires to facilitate the diagnosing of depression.* |
|  |  |  | Sirdifield (2013) | Barriers  ***Benzodiazepines were viewed as safe or unsafe and effective or ineffective depending on the professional or personal experience of the GP. Negative attitudes towards benzodiazepines were based on their perceived risks.***  ***GPs perceived a lack of alternative treatments, depending on their knowledge of alternatives and their*** ***views about validity or effectiveness of non-pharmacological options for particular patients.*** |
|  |  |  | Vedel (2011) | Barriers  ***From the PCPs' point of view, the most often identified physician-related barriers were linked to perception of screening tests (9 studies): absence of belief in the usefulness of cancer screening for older adults and complexity or potential risk of the test.***  ***The barriers to screening identified most often by both patients and PCPs were perception of screening test (23 studies): embarrassment, discomfort or fear of the test, lack of belief in test usefulness for older patients, difficulties to comply with test instructions.***  Facilitators  ***Other major facilitators were linked to perception of cancer screening tests (8 studies): belief in screening usefulness and simplicity and safety of the test.*** |
|  |  |  | Vogt (2005) | Barriers  *Not effective. Statements referring to physicians' expectations about whether discussing smoking would result in smoking cessation.* |
|  |  |  | Zwolsman (2012) | Barriers  *Attitude towards EBM:*  *Limited treatment options and clinical freedom.*  *EBM not helpful.*  *EBM will not benefit practice.*  *Fear of side-effects or harm.* |
|  | **Electronic systems and communication** |  | Lawrence (2016) | Facilitators  ***PCPs suggested improving post-treatment follow-up through a shared data management system, such as the broad integration of electronic medical records and standard communication procedures between PCPs and specialists.*** |
|  |  |  | McDonagh (2018) | Barriers  *Absence of systems to record test offers.*  *A lack of systematic approaches to call and recall for testing made it difficult to audit testing offers and uptake.*  Facilitators  ***Prompts and reminders. Computer prompts/reminders facilitate testing but rely on practices putting systems in place and recognising the risk of prompt fatigue.***  ***System to record offers. The introduction of a system which records testing offers and uptake would facilitate testing, and also prevent multiple offers, which some PCPs feared would lead to offence or irritation.*** |
|  |  |  | Mikat-Stevens (2015) | Barriers  *In addition, PCPs reported having insufficient tools to assess cancer risk and that it was difficult to enter FH information into the electronic health record.* |
|  |  |  | Schadewaldt (2013) | Facilitators  *In addition to face-to-face communication, two studies identified the use of technologies such as messaging systems as beneficial for regular communication.*  *Regular meetings & time to collaborate.* |
|  |  |  | Sinnott (2013) | Facilitators  *Enhanced use of information technology may support more seamless multimorbidity care, by allowing bidirectional communication and local integration between care providers.*  *It was suggested that weighting consultation lengths to the complexity of multimorbidity would facilitate more effective management.* |
|  |  |  | Vedel (2011) | Barriers  *Other barriers were the lack of resources within the practice (7 studies): lack of human resources or equipment, lack of information system, no reception of the test kits by patients.*  Facilitators  *Another facilitator was the presence of resources within the practice (3 studies): presence of facilities to perform the test and use of information systems.* |
|  |  |  | Yeung (2015) (2015) | Facilitators  *Having adequate resources and a recording system to manage patients was identiﬁed as a facilitator.*  *Using technology was suggested as beneﬁcial, such as recording systems to track testing history, or computer prompts as a reminder to offer a test, although this may be vulnerable to fatigue.* |
|  |  |  | Zwolsman (2012) | Barriers  ***It can be subdivided into lacking resources, no access to evidence, and computer- or internet-related problems, and therefore mainly entails technical difficulties.*** |
|  | **Physical environment** |  | De Vleminck (2013) | Facilitators  ***GPs also considered it advantageous if talking about ACP could take place in the home setting.*** |
|  |  |  | Ju (2018) | Factors  *Some GPs who practised in 14 low socioeconomic areas believed that advising lifestyle changes, particularly in terms of diet, were futile as they believed that patients had limited access to healthy food in their local area.* |
|  |  |  | McDonagh (2018) | Facilitators  *Perception that practice population is at low risk, due to age and rural status*. |
|  |  |  | Mikat-Stevens (2015) | Barriers  *PCPs reported an inability to obtain a consultation with clinicians in four studies, noting that the location of the nearest genetics center was too inconvenient for patients to be able to access.*  *Iredale et al. found that rural location played a major role in access issues because of isolation, lack of social networks, poor transport, decreased accessibility, and lack of referral patterns to secondary and tertiary facilities.* |
|  |  |  | O’Brien (2016) | Barriers  *Distance to resources was a barrier for rural practitioners.* |
|  |  |  | Schadewaldt (2013) | Facilitators  *Working in close physical proximity.* |
|  |  |  | Vedel (2011) | Barriers  *Other frequently reported barriers were linked to the type of medical practices (8 studies): lack of routine general examination, lack of incentive fees and solo or rural practice.* |
|  |  |  | Zwolsman (2012) | Barriers  *Rural location.* |
|  | **Workforce and support** |  | McDonagh (2018) | Barriers  *Practice social norms. Working in a practice where chlamydia testing or screening was not the norm and lack of support from colleagues could discourage PCPs.* |
|  |  |  | O’Brien (2016) | Barriers  *Lack of providers and resources with practitioners sometimes becoming the ‘“de facto” mental health provider’ as there ‘simply wasn’t anyone else available’.*  Facilitators  *Collaboration with other professionals and increased providers and resources.* |
|  |  |  | Ogeil (2020) | Barriers  *Practitioners perceived an overreliance on pharmacotherapy and inadequate support to direct patients to alternate pathways.* |
|  |  |  | Sinnott (2013) | Barriers  *Fragmented care resulted from ‘the involvement of several medical specialists, who each emphasize the importance of ‘their’ guideline’ and ‘poor communication from specialists and hospitals to the family physician’ which meant that ‘coordination and overview on medication were hard to maintain’.* |
|  |  |  | Tonkin-Crine (2011) | Factors  *GPs’ perception of their health system and the amount of support offered to them to help change their prescribing behaviour.* |
|  |  |  | Yeung (2015) (2015) | Barriers  ***Insufﬁcient support from practice staff was acknowledged as a barrier to testing overall, and to dedicated chlamydia testing campaigns.***  Facilitators  ***Using a whole-team approach to testing reduced pressures on GPs and empowered other staff members to take on a leading role.*** |
|  |  |  | Zwolsman (2012) | *Barriers*  ***Furthermore, a lack of managerial or institutional support is mentioned as a barrier.*** |
|  | **Processes and pathways** |  | Ju (2018) | Facilitators  *Some GPs used visual prompts to demonstrate to their patients the direct improvements in health and decrease of risk scores, which could be achieved through changes to lifestyle. They believed this approach encouraged patients to make active changes by giving them ‘something positive to cling to’.* |
|  |  |  | Lawrence (2016) | Facilitators  *Provision of patient-specific standard guidelines notated by oncologists or meetings aimed at sharing information and defining roles were also endorsed.* |
|  |  |  | McDonagh (2018) | Barriers  *Both groups emphasised the potential of the chlamydia testing policies (e.g., testing based on patient behaviour; women-only testing) to imply judgements about sexual behaviour and identity, particularly through women-only testing or when sexual history was asked.*  Facilitators  *Having support and pathways for partner notification may encourage more PCPs to offer testing.*  *Offering testing as part of other consultations (e.g. new patients’ health checks, travel vaccination consultations) was considered an enabler to test new patients in the target population and those with who may rarely visit a GP. This approach could also help normalise testing (social opportunity).*  *Blanket testing policies in which all young people are offered a test, which could also reduce automatic motivation barriers for patients (fear of judgement, embarrassment, and shame) and staff should also make this policy clear when offering tests to patients or sending reminder letters.*  *Promotion and discussion of testing at staff practice meetings*  *Fostering a culture of shared learning by talking with staff about difficulties, team huddles prior to clinics, and regular reminders. A flexible approach to testing is also important; practices should adopt a testing policy that suits their patients, practice layout, staffing, and opening times.* |
|  |  |  | Vedel (2011) | Barriers  ***Other frequently reported barriers were linked to the type of medical practices (8 studies): lack of routine general examination, lack of incentive fees and solo or rural practice.*** |
|  |  |  | Yeung (2015) (2015) | Barriers  *A lack of formal practice protocol reduced the ability of nursing staff to carry out chlamydia testing and sexual health related consults.* |
|  |  |  | Zwolsman (2012) | Barriers  *Furthermore, a lack of managerial or institutional support is mentioned as a barrier.* |
| **Social influences** | **Patient/carer characteristics** | **Perceived ideas, concerns, expectations and motivation** | Barley (2011) | *For patients it may be a ‘way out’ of social problems or a way of avoiding work; hence GPs felt many patients seek medicalisation of their problems.*  *Prescribing may be influenced by perceptions of patients’ attitudes to antidepressants.* |
|  |  |  | De Vleminck (2013) | Barriers  *Medium evidence supports that a patient’s denial of his/her terminal illness makes talking about preferences for end-of-life care very difﬁcult.*  *Perceived patient-related obstacles can hinder GPs in initiating ACP. The GP holding the following beliefs is perceived as a barrier and supported by lower evidence: patients lack knowledge of ACP, patients have a fear of upsetting their families, and patients are reluctant to think about future health care problems.* |
|  |  |  | Ju (2018) | Barriers  ***Despite behavioural change being a highly cost-effective prevention strategy, patient motivation and adherence to lifestyle advice is a barrier to preventive care.*** |
|  |  |  | Lawrence (2016) | Barriers  *The major barriers to assuming responsibility at an earlier stage were: patient preference for specialist-led care (65 %), personal knowledge and skills (52 %) and workload pressure (36 %).*  *Patient preference or expectation for specialist-led care identified by 65–72 % of PCPs.* |
|  |  |  | Lucas (2015) | Barriers  ***Perceived pressure from parents was reported as the principle reason to prescribe in several studies, although this did not necessarily imply a stated expectations or desire. This perceived pressure could also result from parental anxiety, fear of litigation, and concern for the consequences for the doctor – patient relationship (including re-consultation).***  *Pressure to give antibiotics to allow return to work or day care.* |
|  |  |  | McDonagh (2018) | Barriers  ***PCP perceptions included believing that patients were at low risk and that chlamydia was not a high priority for patients, particularly in rural areas and areas of high deprivation.***  *Some GPs expressed reluctance to bring up chlamydia or even sexual health during new patient health checks, as they believed it could hinder the development of the doctor-patient relationship (social opportunity) and felt patients would not want information about chlamydia on their health record.*  *Some also believed that patients prefer to access sexual health services from speciality clinics, and if a patient wanted a test, they would request one.* |
|  |  |  | Mikat-Stevens (2015) | Barriers  *Patient not interested in referral or in genetic test.* |
|  |  |  | Ogeil (2020) | Barriers  *In addition, a recent survey of those in family practice and community pharmacists reported that practitioners perceive an overreliance on pharmacotherapy amongst insomnia patients.*  *Barriers to knowledge identified: limited training, lack of resources, patient expectation to receive a pill, consultation time constraint.*  *Patients often have a reliance or expectation of a ‘quick fix’.* |
|  |  |  | Schumann (2012) | Barriers  *They are frequently faced with distressed patients who are concerned about job security, fearful of the label of depression and sceptical of antidepressants.*  *The vague syndromal character of depressive disorders and the individual behaviour and expectations of the patient (whether they mask their symptoms, accept or reject a diagnosis and how they react to a diagnosis) are considered the most powerful barriers.* |
|  |  |  | Sinnott (2013) | Barriers  *This burden was compounded by certain patient characteristics such as cognitive or memory problems, poor social supports and ﬁnances and low levels of motivation which were likely to affect the patient’s ability to understand and adhere to treatment.*  *The importance of eliciting patient’s preferences was widely acknowledged, but GPs had difﬁculties doing this in practice.* |
|  |  |  | Sirdifield (2013) | Factors  ***Prescribing was influenced by how doctors perceived patients’ expectations, motivation and ability to cope.***  *Expectations were sometimes assumed rather than directly discussed.*  *Some patients were felt to be better able or motivated to cope without benzodiazepines or engage with alternative treatments than others.* |
|  |  |  | Tonkin-Crine (2011) | Factors  ***GPs’ perception of patient expectations and experiences of patient demand for antibiotics.***  ***GPs may also feel that delivering patient satisfaction is crucial when they perceive a risk of losing patients to other doctors.*** |
|  |  |  | Vedel (2011) | Barriers  ***Other barriers were linked to patients' attitudes (19 studies): lack of patient motivation to undergo screening, fear of finding cancer, refusal of the test, cultural barriers and fatalism.*** |
|  |  |  | Zwolsman (2012) | Barriers  ***Barriers related to the patient’s preferences. Patient-related barriers limit the use of EBM by GPs. Patients’ preferences, expectations, and beliefs have a major influence on EBM use, according to GPs.*** |
|  |  | **Demographic and socioeconomic factors** | Barley (2011) | Barriers  *High levels of comorbidity within older people may, however, complicate depression diagnosis and lead to delay in treatment.*  *Case-finding tools were not used in older people, despite diagnosis in this group considered especially difficult. Older people were perceived reluctant to accept a diagnosis of depression or to talk about their mood as it would ‘waste’ the doctor’s time. However, such perceptions may be justification for clinicians’ reluctance to make a diagnosis when they feel they have nothing to offer the patient.*  *GPs may be less likely to refer older patients for psychological therapy, either because they ‘forget’ about it or assume it will not work in this population.*  Factors  *Data around gender differences were conflicting. Clinicians were aware of a greater risk of suicide in men, but, where some found men less likely than women to raise psychosocial problems, others reported no differences.* |
|  |  |  | Carlsen (2007) | Barriers  *Convenience, lack of skills with new procedures, and lack of resources, were also referred to: ‘The reason why a lot of time is required was that in elderly patients it was usually more difficult to explain the therapy, and that older patients were less mobile so you had to visit them at home.’* |
|  |  |  | Ju (2018) | Barriers  *Some GPs believed that patients who had established long-term lifestyle patterns in life (particularly patients who were obese and elderly) were unlikely to alter their habits (eg, smoking, diet), and so did not encourage lifestyle changes. They concluded that ‘medications are the only hope’ for patients who they believed were unable to adopt preventive behaviours.*  *Some GPs who practised in low socioeconomic areas believed that advising lifestyle changes, particularly in*  *terms of diet, were futile as they believed that patients had limited access to healthy food in their local area.* |
|  |  |  | McDonagh (2018) | Barriers  ***Gender-related beliefs included a perception that young men did not attend general practice often and that women preferred to see female general practitioners (GPs) for testing which could discourage male practitioners from offering tests.***  ***PCP perceptions included believing that patients were at low risk and that chlamydia was not a high priority for patients, particularly in rural areas and areas of high deprivation.*** |
|  |  |  | O’Brien (2016) | Barriers  *Relating to young people highlighted as difficult.* |
|  |  |  | Sinnott (2013) | Barriers  *This burden was compounded by certain patient characteristics such as cognitive or memory problems, poor social supports and ﬁnances and low levels of motivation which were likely to affect the patient’s ability to understand and adhere to treatment.*  Factors  *Multimorbid patients that GPs felt required particular assistance are those with cognitive impairment, mental health issues or low social support, and accordingly may require nuanced interventions to support their care.* |
|  |  |  | Sirdifield (2013) | Barriers  *Particular patient attributes, including old age, multiple conditions, and being perceived as a ‘deserving patient’ also increased the pressure to prescribe and gave a rationale to do so.*  *GPs often felt greater (or perceived greater public) sympathy but also felt a lack of alternatives for older compared with younger patients: “Physicians thought their older adult patients would resist or be unable to pursue mental health referrals for multiple reasons ranging from stigma to financial and transportation difficulties”.* |
|  |  |  | Tonkin-Crine (2011) | Factors  *GPs’ prescribing decisions may be inﬂuenced by the patient’s work, social life or socio-economic background in addition to their illness.* |
|  |  |  | Vedel (2011) | Barriers  *Physicians also considered age of an older patient as a reason not to screen.* |
|  |  | **Health status and comorbidity** | Barley (2011) | Barriers  *High levels of comorbidity within older people may, however, complicate depression diagnosis and lead to delay in treatment.* |
|  |  |  | Ju (2018) | Barriers  *Some GPs believed that patients who had established long-term lifestyle patterns in life (particularly patients who were obese and elderly) were unlikely to alter their habits (eg, smoking, diet), and so did not encourage lifestyle changes.* |
|  |  |  | Sinnott (2013) | Barriers  *This burden was compounded by certain patient characteristics such as cognitive or memory problems, poor social supports and ﬁnances and low levels of motivation which were likely to affect the patient’s ability to understand and adhere to treatment.*  Factors  *Multimorbid patients that GPs felt required particular assistance are those with cognitive impairment, mental health issues or low social support, and accordingly may require nuanced interventions to support their care.* |
|  |  |  | Sirdifield (2013) | Barriers  *Particular patient attributes, including old age, multiple conditions, and being perceived as a ‘deserving patient’ also increased the pressure to prescribe and gave a rationale to do so.* |
|  |  |  | Vedel (2011) | Facilitators  ***The patient's characteristics was the most frequently identified facilitator for screening, as reported by both PCPs and patients (15 studies): presence of risk factors and good health status.*** |
|  |  | **Culture and religion** | McDonagh (2018) | Barriers  *Patient cultural and religious factors could also act as a barrier to testing.* |
|  |  |  | Mikat-Stevens (2015) | Barriers  *PCPs reported that their patients’ accuracy when providing FH information was a barrier as were language and culture barriers when discussing FH and genetics with patients.* |
|  |  |  | Vedel (2011) | Barriers  *Other barriers were linked to patients' attitudes (19 studies): lack of patient motivation to undergo screening, fear of finding cancer, refusal of the test, cultural barriers and fatalism.* |
|  |  |  | Yeung (2015) (2015) | Barriers  *The practicalities of offering a test were magniﬁed in patients with comprehension issues or cultural barriers.* |
|  |  | **Fear and ability to cope** | Sinnott (2013) | Barriers  *GPs reported that many patients actively participate in decision-making, can prioritise and are ‘good with trial and error’. However, for certain patients making choices could be a ‘source of distress’ and contributed to them becoming ‘over the top anxious about their conditions’.* |
|  |  |  | Sirdifield (2013) | Barriers  *Some patients were felt to be better able or motivated to cope without benzodiazepines or engage with alternative treatments than others.* |
|  |  |  | Tonkin-Crine (2011) | Factors  *GPs’ prescribing decisions may be inﬂuenced by a patient’s fear or concerns about their illness.* |
|  |  |  | Vedel (2011) | Barriers  *Other barriers were linked to patients' attitudes (19 studies): lack of patient motivation to undergo screening, fear of finding cancer, refusal of the test, cultural barriers and fatalism.* |
|  |  | **Education and awareness** | McDonagh (2018) | Barriers  *Lack of patient (and public) education, knowledge, and awareness. This was reported as a barrier by PCPs.* |
|  |  |  | O’Brien (2016) | *Increased parental awareness of mental health problems was endorsed as a facilitator.* |
|  |  |  | Vedel (2011) | Facilitators  ***Another frequently reported facilitator was the older adult's awareness/knowledge of prevention, screening and cancer (7 studies).*** |
|  |  |  | Yeung (2015) (2015) | *Facilitators were also identified – financial incentives, patient education or awareness about testing, and computer prompts/reminders to test*. |
|  |  | **Diagnostic group** | Barley (2011) | Barriers  ***Negative attitudes included unfavourable views of depressed people themselves e.g. ‘burdens’, ‘not particularly attractive’, ‘people who bore you’, pessimism concerning outcomes, feelings of the work being unrewarding.***  ***Among some clinicians, ambivalent attitudes to working with depressed people, a lack of confidence, the use of a limited number of management options and a belief that a diagnosis of depression is stigmatising complicate the management of depression.*** |
|  |  |  | De Vleminck (2013) | Facilitators  *According to GPs, cancer patients are more involved in the process of ACP than non-cancer patients. As they often have a more predictable disease course, deﬁning the right moment to initiate ACP might be easier.* |
|  | **GP-patient relationship and patient-centred care** | **GP-patient relationship and patient-centred care** | Barley (2011) | Facilitators  *Management strategies An individualised approach based on a wide range of management options was favoured.* |
|  |  |  | Carlsen (2007) | Barriers  ***Preserving the doctor–patient relationship In some of the studies, fear of jeopardising the relationship with the patient was mentioned by GPs as a reason for non-adherence.*** |
|  |  |  | De Vleminck (2013) | Barriers  *Fear of depriving a patient of hope or damaging the GP–patient relationship were cited as factors that keep GPs from engaging in the process of ACP, for which respectively stronger and medium evidence was found.*  Facilitators  ***Stronger evidence supported a longstanding patient–GP relationship as a perceived facilitator for ACP.*** |
|  |  |  | Ju (2018) | Factors  ***Providers considered preventive strategies in the context of tensions between respecting patient autonomy and being too intrusive and paternalistic in recommending behavioural change.***  Facilitators  ***Negotiating patient acceptance When developing a strategy for preventing CVD, some GPs perceived that compromise was necessary in encouraging patients to cooperate. An explicit discussion and consideration of the patient’s goals and priorities was seen to encourage patients to ‘work with the doctor, not against the doctor’ which built trust. Some GPs coproduced a strategy with the patient that was feasible for the patient’s own situation.*** |
|  |  |  | Lawrence (2016) | Barriers  ***Lack of patient trust.***  *Concern for the consequences for the doctor – patient relationship (including re-consultation).* |
|  |  |  | Lucas (2015) | Barriers  *Perceived pressure from parents was reported as the principle reason to prescribe in several studies, although this did not necessarily imply a stated expectations or desire. This perceived pressure could also result from parental anxiety, fear of litigation, and concern for the consequences for the doctor – patient relationship (including re-consultation).*  Facilitators  *Parents sought a thorough medical assessment from a doctor they trusted, and were satisﬁed with treatment decisions which differed from their expectations where this was the case.* |
|  |  |  | McDonagh (2018) | Barriers  ***Some PCPs were unwilling to introduce sexual health during new patient checks in case it affected the doctor-patient relationship.*** |
|  |  |  | O’Brien (2016) | Barriers  *Difficulties ‘establishing a rapport.*  Facilitators  *A longstanding relationship with the family strengthened the [practitioner’s] commitment’ and provided the advantage of contextual knowledge.* |
|  |  |  | Schumann (2012) | Barriers  *When clinicians were not familiar with the patient, they acknowledged that the patient was less likely to share personal information, making diagnosis even more difﬁcult.*  Facilitators  ***Furthermore, establishing a rapport with their patients and a trusting physician–patient relationship was essential for the diagnostic process. A relationship that has developed over the years was usually believed to help reveal symptoms of depression.*** |
|  |  |  | Sinnott (2013) | Facilitators  *In most studies, the longitudinal nature of the patient– GP relationship was seen as a ‘major facilitator’ and ‘elementary component’ of patient-centred care in multimorbidity.*  Barriers  ***Challenges in delivering patient-centred care: Patient centredness is an over-riding principal for GPs in multimorbidity but trying to achieve this increases the complexity of care in some cases, and can lead the GP into additional conflict with specialist services or evidence-based medicine.***  ***Challenges in shared decision-making: The patient’s role in decision-making in multimorbidity is limited by difficulties in communicating risk benefit and outcomes in a field where there is much more uncertainty on these issues.***  *Within the speciﬁc context of deprivation, longitudinal care was ‘potentially transformative’ by providing ‘time to build relationships with patients’ but it was also a source of problems, by creating dependence and increased demands by patients for consultations.* |
|  |  |  | Sirdifield (2013) | Barriers  ***Concepts such as patient-centred practice and perceptions of patients’ expectations of benzodiazepine prescribing competed with GPs rationing role, autonomy, attitude to benzodiazepines, and wish to maintain good doctor-patient relationships – sometimes through giving patients their desired ‘quick-fix’.***  *Time limited consultations (a barrier to alternative interventions), or the prospect of unhappy patients leaving for other practices, influenced whether or not GPs initiated withdrawal.*  Facilitators  *A GP knowing a patient well and/or empathising with their situation, increased the likelihood of breaking previous ‘rules’ about what constituted a ‘deserving patient’: “The following case shows that in some contexts the normal aversion to offering the drugs to patients with a drink problem noted earlier can be overridden by some GPs sympathetic to the personal plight of some patients…”* |
|  |  |  | Tonkin-Crine (2011) | Factors  *GPs’ opinion of the importance of patient satisfaction and the perception of whether patients will go elsewhere for treatment.*  Facilitators  *Lastly, delayed prescribing may provide patient reassurance, by giving the patient control over their treatment.*  ***Interventions may encourage more patient-centred care, allowing GPs to understand patient wishes and offer reassurance without recourse to antibiotics; GPs report a more holistic approach when assessing a patient’s needs.*** |
|  |  |  | Vedel (2011) | Facilitators  *Other facilitators are patients' attitudes (7 studies): western culture, motivation to undergo the test, patient's request of the test, trust in provider; and patients' perceptions of screening tests.* |
|  |  |  | Yeung (2015) (2015) | Factors  *GPs feared offending their patients by offering testing. They worried that patients might perceive them as being discriminatory by judging them as likely to be infected. Sensitivity for both sides was increased when religious and cultural norms differed from one another, particularly if the patient was of non-heteronormative orientation. Moreover, when a patient is known to a GP socially, a GP may not ask to prevent an uncomfortable situation.* |
|  |  |  | Zwolsman (2012) | Barriers  *Relationship between GP and patient.* |
|  |  | **Continuity of care** | Carlsen (2007) | Facilitators  ***However, a longlasting and trusting doctor–patient relationship could be judged by the GP as strong enough to endure a rationing decision. Continuity of care could thus enhance guideline adherence.*** |
|  |  |  | Lawrence (2016) | Barriers  *One study reported that PCPs were prepared to accept exclusive responsibility for routine follow-up approximately two to four years from the completion of active treatment; however, another reported that less than half of participants were willing to provide such care five years post treatment.*  Facilitators  *PCPs considered the advantages of primary-cared based follow-up to include: pre-existing relationships with patients (82 %), accessibility (69 %) and lower costs (66 %)*. |
|  |  |  | Lucas (2015) | Factors  *Where clinicians felt uncertain of the consequences of not prescribing they would also sometimes prescribe. This occurred both where diagnosis was unclear, and also where they were uncertain that parents could manage the illness themselves, particularly where there was no continuity of care so doctor and parent did not know each other.* |
|  |  |  | Schumann (2012) | Facilitators  *A relationship that has developed over the years was usually believed to help reveal symptoms of depression. When clinicians were not familiar with the patient, they acknowledged that the patient was less likely to share personal information, making diagnosis even more difﬁcult.* |
|  |  |  | Sinnott (2013) | Facilitators  *In most studies, the longitudinal nature of the patient– GP relationship was seen as a ‘major facilitator’ and ‘elementary component’ of patient-centred care in multimorbidity.*  *Three subtypes of continuity of care have been previously described; of these, both informational and management continuity were seen here as necessary for patient safety and cohesive management. However, it was relational continuity that appeared to most facilitate care in multimorbidity, by allowing GPs to foster trust, anticipate preferences and empower their patients over time.* |
|  | **Collaboration and communication with other health professionals** | **Collaboration and communication with other health professionals** | De Vleminck (2013) | Barriers  *There is lower evidence that lack of collaboration with secondary care is perceived as an impediment to the process of ACP.*  Facilitators  *Consultation with other healthcare professionals and hospital policy supporting or requiring the use of ADs was considered as a facilitator, supported by medium evidence.* |
|  |  |  | Ju (2018) | Facilitators  ***‘Working together’ with specialists meant reinforcing, to the patient, the specialist’s advice and GPs believed that this would strengthen cohesive care for the patient.*** |
|  |  |  | Lawrence (2016) | Barriers  ***PCPs reported that they rarely or inconsistently received sufficient correspondence from oncologists. Though oncologists’ letters contained details on investigation and examination findings, items such as treatment/management plans, future management and expectations, goals and procedures of follow-up, and psychosocial concerns were less commonly mentioned.***  Facilitators  ***PCPs strongly endorsed improved communication with oncologists so that they could provide improved care for their patients.*** |
|  |  |  | McDonagh (2018) | Barriers  *Working in a practice where chlamydia testing or screening was not the norm and lack of support from colleagues could discourage PCPs.*  Facilitators  ***The involvement of PNs was viewed positively by both GPs and PNs.*** |
|  |  |  | O’Brien (2016) | Barriers  ***Lack of communication led to a disconnect between primary and secondary care and ‘contributed to primary care practitioners’ perceptions of poor effectiveness of therapy’.***  Facilitators  ***Collaboration with other professionals and increased providers and resources.***  ***Desire for increased communication, information, and feedback on referrals.*** |
|  |  |  | Ogeil (2020) | Facilitators  *Greatest influence on changing practice style regarding sleep were journal articles followed by continuing education, followed by discussion with specialists.* |
|  |  |  | Schadewaldt (2013) | Facilitators  ***Knowing the NP/MP & good working relationship.*** |
|  |  |  | Sinnott (2013) | Barriers  ***Fragmented care resulted from ‘the involvement of several medical specialists, who each emphasize the importance of ‘their’ guideline’ and ‘poor communication from specialists and hospitals to the family physician’ which meant that ‘coordination and overview on medication were hard to maintain’.***  ***This problem is compounded by poor co-ordination and communication within the health service, leaving GPs feeling excluded from their patients’ care and with a sense of uncertainty regarding their role.***  Facilitators  *Despite these reservations, the input of specialists was desired. A ‘balance of equals’ was called for, that would allow GPs and specialists to discuss complex patients and improve the awareness of complexity in multimorbidity among specialists. This would help all doctors involved ‘to speak with one voice. Different stories provoke distrust’.* |
|  |  |  | Tonkin-Crine (2011) | Factors  **Discussion with colleagues and knowledge of how others’ practice may inﬂuence GPs’ prescribing decisions.**  **In contrast, GPs appear to regard the opinions and actions of peers highly. Discussion and comparison with peers may be very influential because of a GP’s motivation to fit with professional norms.**  Facilitators  *Personal or local feedback allows an opportunity for GPs to reﬂect on their own prescribing and potential effects, and offers motivation to change.* |
|  |  |  | Zwolsman (2012) | Factors  *The opinion of primarily colleagues about EBM is considered important in the use of EBM because the attitude of these colleagues influences the practice of EBM.* |
|  |  | **Hierarchy and power** | McDonagh (2018) | Barriers  *GP negative attitudes towards PNs -role conflict and handing over power*. |
|  |  |  | Schadewaldt (2013) | Barriers  ***NPs often perceived a hierarchical relationship with the MP that was described as a power struggle for NPs and experienced by NPs when the MP decided over the range of tasks to be undertaken by the NP.***  ***While the reciprocity of referrals and consultations as well as the absence of hierarchical structures were considered to foster collaboration, NPs and MPs also reported control issues as a barrier to collaborative practice.***  Facilitators  ***Reciprocity (including the absence of hierarchy & control)*** |
|  | **Communication with patients and consultation context** |  | Barley (2011) | Barriers  *However, some clinicians considered their patients unable to open up [32,39] or reported an inability to empathise with a patient’s chosen lifestyle.*  *Interactional difficulties with depressed people*.  *Cues to depression were found to arise slowly, with patients often raising the issue when preparing to leave.* |
|  |  |  | De Vleminck (2013) | Barriers  *Difﬁculties with deﬁning the right moment.*  *Experiencing difﬁculties with advising patients in expressing their wishes.* |
|  |  |  | McDonagh (2018) | Barriers  ***Some, especially older male PCPs found it difficult to discuss sexual health with patients due to personal discomfort. This was particularly a concern in consultations with male patients and in consultations unrelated to sexual health.*** |
|  |  |  | Mikat-Stevens (2015) | Barriers  *Patients are unable to provide accurate family history information.*  *Not enough confidence in accuracy of information being provided.* |
|  |  |  | O’Brien (2016) | Barriers  *Children’s inability to express themselves well.*  *Difficulties ‘establishing a rapport, finding the right words and tone to use and ‘dealing with silence’ with younger patients.* |
|  |  |  | Schumann (2012) | Barriers  *Throughout all studies, the symptoms of depression were described as ‘vague’ or ‘subjective’ and as frequently masked by physical symptoms. Patients seen in primary care settings often reported symptoms that were unclear and could indicate any of several diagnoses, including depression.* |
|  |  |  | Sinnott (2013) | Facilitators  *Enhanced-communication skills were seen as necessary in multimorbidity to facilitate clear and concise discussion with patients on the interplay between their chronic diseases and to help with de-prescribing medications, which if carried out badly could be interpreted as withdrawing care.* |
|  |  |  | Tonkin-Crine (2011) | Factors  *GPs’ perceptions of how easy or difﬁcult it is to explain prescribing decisions to patients.*  Facilitators  *Communication skills allow GPs to offer individualized explanations to patients, which address their personal concerns and probably raise satisfaction.* |
|  |  |  | Yeung (2015) (2015) | Facilitators  *One overarching concept became evident in this review; because the primary interaction is between the GP and patient, communication is a key stone in the foundation of chlamydia testing. Without either participant in the GP–patient relationship communicating the need for testing, chlamydia testing will never be on the agenda.* |
|  |  |  | Zwolsman (2012) | Barriers  *Information retrieval by patient.* |
|  | **Norms, stigma and attitudes** |  | Barley (2011) | Barriers  ***Stigma was considered more important for some ethnic groups (Caribbean and South Asian). Stigma in these communities was seen as a barrier to addressing psychosocial aspects of the illness and to beginning treatment.***  ***Attitudes were diverse. Negative attitudes included unfavourable views of depressed people themselves e.g. ‘burdens’, ‘not particularly attractive’, ‘people who bore you’, pessimism concerning outcomes, feelings of the work being unrewarding.*** |
|  |  |  | Ju (2018) | Barriers  *In earlier studies, GPs expressed more hesitation about prescribing medications, when this was not yet common practice nor widely recommended for primary prevention.* |
|  |  |  | McDonagh (2018) | Barriers  *Cultural norms within a practice were discussed in two studies and an environment where testing is not a high priority was seen as a deterrent.*  Facilitators  ***Normalising chlamydia testing for patients, and at service level was raised as a way of destigmatising chlamydia infection and facilitating testing. Services in which testing was part of everyday practice (e.g., new patient checks, travel vaccinations, or young people’s clinics) reported high levels of testing.*** |
|  |  |  | Mikat-Stevens (2015) | Barriers  *Language and culture challenges when discussing family history and genetics.*  ***The second most common ELSI barrier (n = 15 citations in 15/38 studies) was the fear of social or insurance discrimination for their patients as a result of their genetic information. Freedman et al. found that more than 80% of 1,251 US PCPs surveyed thought that patients with positive genetic testing results were at risk for insurance discrimination.*** |
|  |  |  | O’Brien (2016) | Barriers  *Reluctance of society to see eating disorders as a serious disease was ‘a severe hindrance’.*  *Stigma and negative consequences of labelling.* |
|  |  |  | Schumann (2012) | Barriers  *Barriers resulting from the health system or society encompassed fear of stigmatization and (premature) labelling, future insurability, reimbursement, and lack of time and resources.* |
|  |  |  | Sirdifield (2013) | Barriers  *GPs often felt greater (or perceived greater public) sympathy but also felt a lack of alternatives for older compared with younger patients: “Physicians thought their older adult patients would resist or be unable to pursue mental health referrals for multiple reasons ranging from stigma to financial and transportation difficulties”.*  *Some alternatives were seen as less ‘valid’ due to inaccessibility, stigma and costs for patients.*  Factors  *GPs perceived the context for benzodiazepine prescribing decisions had changed over time because of changing: norms of practice, evidence, guidance (national and local), introduction of new drugs (e.g. selective serotonin reuptake inhibitors) and services, legal regulatory frameworks and the societal attitudes for treatment of conditions including anxiety, depression and insomnia.* |
|  |  |  | Tonkin-Crine (2011) | Factors  ***In contrast, GPs appear to regard the opinions and actions of peers highly. Discussion and comparison with peers may be very influential because of a GP’s motivation to fit with professional norms.*** |
|  |  |  | Vedel (2011) | Barriers  *Other barriers were linked to patients' attitudes (19 studies): lack of patient motivation to undergo screening, fear of finding cancer refusal of the test, cultural barriers and fatalism.*  Facilitators  *Other facilitators are patients' attitudes (7 studies): western culture, motivation to undergo the test, patient's request of the test, trust in provider; and patients' perceptions of screening tests (4 studies): belief in test usefulness and comfort with test.* |
|  |  |  | Yeung (2015) (2015) | Barriers  ***GPs feared offending their patients by offering testing. They worried that patients might perceive them as being discriminatory by judging them as likely to be infected.***  ***Difficulty in discussing sexual health with patients who are non-heterosexual, related to GP attitudes and awareness towards same-sex relationships, due to lack of knowledge around same-sex lifestyles, practices and terminology.***  Facilitators  ***The normalisation of testing may prove to be the most important facilitator of chlamydia testing.***  ***Electronic alerts have been shown to increase testing rates (Walker et al. 2010), and with the use of non-heteronormative terminology, these are theorised to also reduce the stigma around testing by reducing judgment towards the patient to normalise testing.*** |
|  |  |  | Zwolsman (2012) | Barriers  *Personal and organisational inertia.* |
| **Emotion** | **PCP emotions** |  | Barley (2011) | Factors  *For the GP, giving a diagnosis of depression allows them to follow a pre-determined treatment plan and to avoid feelings of powerlessness.* |
|  |  |  | Carlsen (2007) | Barriers  *Defensive practice included the emotional burden of missing a diagnosis, and fear of litigation. This was particularly the case when guidelines supported rationing. Although there was some sense that guideline adherence could protect the GP in a possible legal process, defensive practice seemed to be a more common strategy than following guidelines.*  *GPs’ desire to respond to patients’ needs and requests sometimes conflicted with the guideline recommendations, and empathy for patients that are suffering or anxious was mentioned as a factor that overruled guidelines in decision making.* |
|  |  |  | De Vleminck (2013) | Barriers  *Medium evidence was found that they perceive their lack of skill in dealing with a patient’s changing preferences and with the emotional impact or discomfort of having ACP discussions as barriers.* |
|  |  |  | Ju (2018) | Factors  *In a study on the prevention of type 2 diabetes, 38 GPs questioned their role and obligation in preventive care, where some expressed frustration at the societal pressure placed on them to screen patients for health risks despite the lack of funding and resources.* |
|  |  |  | Lawrence (2016) | Barriers  *PCPs expressed frustration with delays in information transfer and a lack of coordination of care.* |
|  |  |  | McDonagh (2018) | Barriers  *Discomfort with raising the issue of testing.* |
|  |  |  | Schumann (2012) | Barriers  *‘If clinicians felt rushed and were catching up when they encountered such a patient, they were unlikely to*  *open the door’. This means they actually do not have the time, they needed to let patients divulge their problem. Some studies reported that FPs described the process as a draining and somewhat frustrating experience.* |
|  |  |  | Sinnott (2013) | Factors  *GPs used modiﬁed approaches to guidelines, involving, for example, the estimation of risk associated with particular diseases/treatments. However, some felt that this modiﬁcation was in conﬂict with ‘best practice’ and felt guilt at not implementing guidelines fully.* |
|  |  |  | Sirdifield (2013) | Factors  *Thus, whereas doctors may, in general, be more conservative in their decisions for patients than they would be for themselves, strong feelings of empathy for the patient may lead doctors to be more likely to offer a prescription.* |
|  |  |  | Tonkin-Crine (2011) | Factors  *GPs’ emotional and physical state at the time of consultation may affect the prescribing decision (e.g. stress, tiredness).* |
|  |  |  | Vogt (2005) | Factors  *Expressions of positive or negative emotional experiences of discussing smoking cessation.* |
|  |  |  | Yeung (2015) (2015) | Barriers  *Proportion of GPs feeling uncomfortable in taking a sexual history.*  *Proportion citing embarrassment as a barrier.* |
|  | **Patient/carer emotions** |  | Carlsen (2007) | Factors  *GPs’ desire to respond to patients’ needs and requests sometimes conflicted with the guideline recommendations, and empathy for patients that are suffering or anxious was mentioned as a factor that overruled guidelines in decision making*. |
|  |  |  | Ju (2018) | Barriers  *Regardless of the patient’s level of risk for CVD, some GPs urged to avoid instilling unnecessary anxiety in patients, as ‘fear becomes a major problem’ and in turn elevates their risk further.* |
|  |  |  | Lucas (2015) | Factors  *Perceived pressure from parents was reported as the principle reason to prescribe in several studies, although this did not necessarily imply a stated expectations or desire. This perceived pressure could also result from parental anxiety, fear of litigation, and concern for the consequences for the doctor – patient relationship (including re-consultation).* |
|  |  |  | McDonagh (2018) | Barriers  *Testing policies which are based on sexual behaviour had the potential to cause offence to patients, made PCPs feel uncomfortable, and were felt to evoke embarrassment and shame for the patient.* |
|  |  |  | Mikat-Stevens (2015) | Barriers  ***Genetic evaluation will cause patient anxiety. Genetic testing will cause patients anxiety.***  **High false positive rate might cause patients unnecessary anxiety.**  **The two most significant barriers related to the concern for patient anxiety and a fear of health insurance or social discrimination.** |
|  |  |  | O’Brien (2016) | Barriers  *Reluctance to broach the issue [of mental health] for fear of provoking ‘defensiveness and anxiety’ in the young person.* |
|  |  |  | Sinnott (2013) | Barriers  *GPs reported that many patients actively participate in decision-making, can prioritise and are ‘good with trial and error’. However, for certain patients making choices could be a ‘source of distress’ and contributed to them becoming ‘over the top anxious about their conditions’.* |
|  |  |  | Tonkin-Crine (2011) | Factors  *GPs’ prescribing decisions may be inﬂuenced by a patient’s fear or concerns about their illness.* |
|  |  |  | Vedel (2011) | Barriers  ***The barriers to screening identified most often by both patients and PCPs were perception of screening test (23 studies): embarrassment, discomfort or fear of the test, lack of belief in test usefulness for older patients, difficulties to comply with test instructions.*** |
|  |  |  | Yeung (2015) (2015) | Barriers  *Proportion of GPs that perceived the patient to be more embarrassed were less likely to take a sexual history than GPs who perceived the patient to not be embarrassed at all.* |
| **Behavioural regulation** | **Medicolegal risk and legislation** |  | Carlsen (2007) | Factors  ***Defensive practice included the emotional burden of missing a diagnosis, and fear of litigation. This was particularly the case when guidelines supported rationing. Although there was some sense that guideline adherence could protect the GP in a possible legal process, defensive practice seemed to be a more common strategy than following guidelines.*** |
|  |  |  | De Vleminck (2013) | Facilitators  *GPs reported that legislation supporting the use of ADs as well as protecting GPs who follow them would encourage them to offer ADs to patients.* |
|  |  |  | Lawrence (2016) | Barriers  *Increased medicolegal risk.* |
|  |  |  | Lucas (2015) | Barriers  *This perceived pressure could also result from parental anxiety, fear of litigation, and concern for the consequences for the doctor – patient relationship (including re-consultation).* |
|  |  |  | Mikat-Stevens (2015) | Barriers  *The concern over provider liability.* |
|  |  |  | Schadewaldt (2013) | Barriers  ***The fourth common obstacle to work in collaborative practice with a NP was the concern of MPs about legal responsibility. Most considered themselves liable for the care provided by the NP.*** |
|  |  |  | Sirdifield (2013) | Factors  *GPs perceived the context for benzodiazepine prescribing decisions had changed over time because of changing: norms of practice, evidence, guidance (national and local), introduction of new drugs (e.g. selective serotonin reuptake inhibitors) and services, legal regulatory frameworks and the societal attitudes for treatment of conditions including anxiety, depression and insomnia.* |
|  |  |  | Vedel (2011) | Barriers  *Some barriers are linked to physician's attitudes (9 studies): priority given to presenting problem, worries about malpractice in performing an invasive test and the risk of legal action, lack of agreement with current guidelines.* |
|  |  |  | Yeung (2015) (2015) | Barriers  *Finally, legal concerns about conﬁdentiality and privacy also hindered a GP’s ability to carry out testing, particularly around partner notiﬁcation.* |
|  |  |  | Zwolsman (2012) | Barriers  *Fear of punishment or litigation.* |
| **Intentions** |  |  | Carlsen (2007) | Barriers  *Interestingly, two studies showed that guidelines could be marshalled as a negotiating tactic when refusing patients’ requests.* |
|  |  |  | Ju (2018) | Barriers  *Harnessing the power of fear When managing patients at high risk of CVD, some GPs felt that scaring patients into action was necessary and warranted. They believed that an emphasis on the consequences of disregarding and being non-adherent to prevention strategies motivated patients to accept their advice, telling their patients ‘if you don’t want that kind of scenario you do what I tell you’.* |

Evidence table mapping quotes to Theoretical Domains Framework (TDF) domain, theme and subtheme. Important or salient quotes are indicated in bold.
